# Necroptosis is a key contributor to impaired cardiac repair following myocardial infarction

**DOI:** 10.1038/s41419-026-08979-5

**Published:** 2026-06-15

**Authors:** Razoan Al Rimon, Yingxi Li, Ilamaran Meganathan, Faqi Wang, Allan G. Murray, Gavin Y. Oudit, Slava Epelman, Xavier Clemente-Casares, Zamaneh Kassiri

**Affiliations:** 1 Department of Physiology, Faculty of Medicine and Dentistry, University of Alberta, Edmonton, AB Canada; 2 Department of Medicine, Faculty of Medicine and Dentistry, University of Alberta, Edmonton, AB Canada; 3 Department of Immunology, University of Toronto, Ted Rogers Centre for Heart Failure Research, Peter Munk Cardiac Centre, Toronto, ON Canada; 4 Department of Medical Microbiology and Immunology, Faculty of Medicine and Dentistry, University of Alberta, Edmonton, AB Canada

**Keywords:** Myocardial infarction, Necroptosis

## Abstract

Myocardial infarction (MI) is a leading cause of morbidity and death worldwide. Endothelial cells (ECs) contribute to post-MI remodeling through angiogenesis, inflammation, and endothelial-to-mesenchymal transition (EndMT). ADAM17, a membrane-bound protease, is upregulated in ischemic heart disease, but its role in endothelial function post-MI is unknown. We investigated whether loss of endothelial ADAM17 could improve post-MI recovery using male and female mice with inducible endothelial-specific ADAM17 knockdown (*Adam17*^f/f^/*Cdhr5-*Cre^ERT2^; *Adam17*^EC-KD^). Surprisingly, male *Adam17*^EC-KD^ mice exhibited compromised post-MI survival (42% death due to LV rupture vs. 13%), and progressive decline in cardiac function compared to controls (*Adam17*^f/f^-MI). Post-MI rupture was less drastic but detected in female *Adam17*^EC-KD^-MI mice. *Adam17*^EC-KD^ hearts exhibited increased neutrophil infiltration, NETosis, and cytotoxic CD8^+^ T-cell accumulation post-MI; however, depletion of these immune cells did not improve post-MI survival. Single-nuclei RNA-seq analyses identified suppression of pro-angiogenic and EndMT markers, and emergence of an EC subpopulation enriched for necroptotic markers. Decreased vascularization was confirmed in the infarcted myocardium with reduced coronary density (CD31 staining; 3-D micro-CT) and pVEGFR2 signaling. Suppressed EndMT in *Adam17*^EC-KD^ mice was linked to reduced collagen crosslinking, decreased activation of the SMAD pathway (pSMAD2/3), decreased expression of lysyl oxidase and Fibronectin in infarcted myocardium. In EC-fibroblast co-cultures in vitro, endothelial *Adam17* knockdown suppressed tubular formation in hypoxic conditions and reduced EndMT. Conditioned media from hypoxic EC^*Ad17*-KD^ suppressed fibroblast activation. Increased necroptosis in vivo (*Adam17*^EC-KD^-MI), and in vitro (EC^*Ad17*-KD^±hypoxia), was associated with increased TNFR1-RIPK3-RIPK1-MLKL signaling due to stabilization of TNFR1 in the absence of its ADAM17-mediated shedding. The critical role of necroptosis in impaired post-MI recovery was confirmed as inhibition of necroptosis (necrostatin-1) markedly improved post-MI survival and coronary vascularization in *Adam17*^EC-KD^-MI hearts. This study demonstrates that ADAM17 regulates post-MI endothelial functions, necroptosis, vascularization, and EndMT, with necroptosis as a critical factor in post-MI adverse myocardial remodeling and survival.

**Figure:**
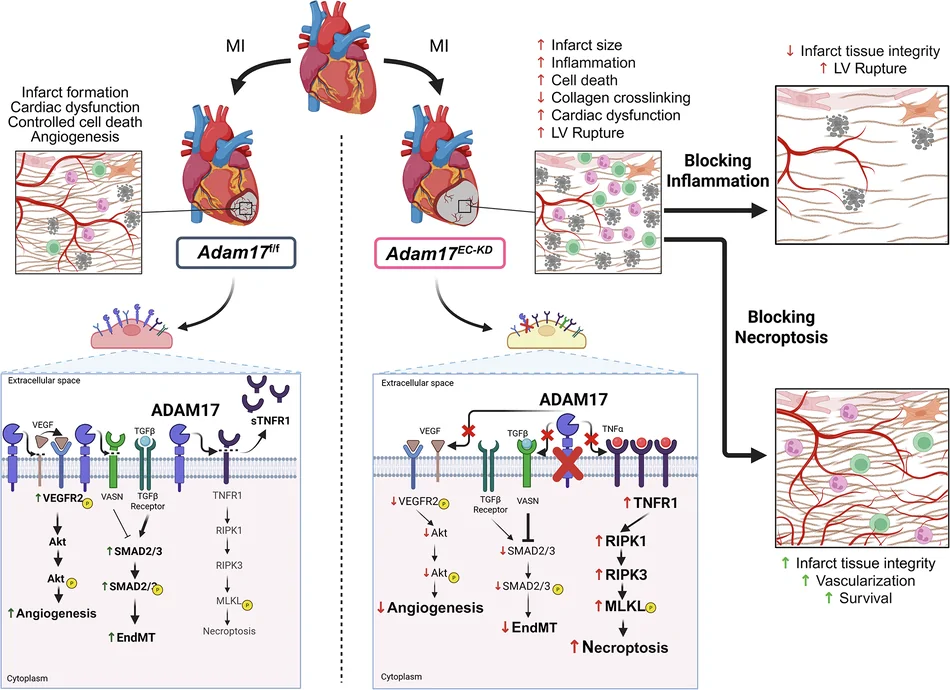

## Introduction

Myocardial infarction (MI) remains one of the most common causes of morbidity and mortality worldwide. It is caused by the disruption of blood flow to the heart muscle, causing ischemic damage, loss of cardiomyocytes, and pathological structural and functional changes leading to heart failure. Post-MI remodeling of the myocardium consists of an intricate series of overlapping cellular events. An acute inflammatory phase is initiated in response to loss of cardiomyocytes and the extracellular matrix (ECM), followed by a reparative phase when inflammation is resolved, activated fibroblasts proliferate, and neovascularization begins, and infarct (scar) tissue develops and matures [1–3]. Optimal infarct formation requires the timely orchestration of the inflammatory and the reparative processes, whereas a deviation in this process can lead to infarct rupture or expansion, and eventually heart failure.

Endothelial cells (ECs) are responsible for vascular homeostasis in health, and neovascularization post-MI. Hypoxia in the ischemic myocardium can stimulate EC proliferation and neovascularization for perfusion recovery [4–6]. ECs also control tissue inflammation post-MI by increasing expression of adhesion molecules, VCAM1, ICAM1, and CXCR3, which mediate recruitment of immune cells into the damaged myocardium [7, 8]. In addition, endothelial-to-mesenchymal transition (EndMT) enables the ECs to acquire mesenchymal features with fibroblast-like properties contributing to ECM remodeling [9, 10]. These events can also be influenced by the survival of ECs as they are exposed to extreme stress post-MI, given the ischemic and inflammatory microenvironment in the affected myocardium [11]. Thus, the function and fate of ECs can critically influence the post-MI myocardial recovery.

A number of ADAMs (a disintegrin and metalloproteinase) are expressed in the heart and have been linked to cardiac diseases [12–14]. ADAM17 is a sheddase with a pivotal role in physiological and pathological conditions owing to its broad range of substrates, including cytokines, growth factors, and adhesion molecules [15–19]. Increased ADAM17 activity has been associated with adverse ventricular remodeling and poor clinical outcomes in post-MI patients [20–22]. Loss of ADAM17 in cardiomyocytes led to infarct expansion and compromised cardiac function [18]. Studies in various tissues have reported that ADAM17 can regulate EC proliferation and migration [23, 24], influence inflammatory cell recruitment via shedding of adhesion molecules [16, 25, 26], and mediate EndMT in gastric carcinoma cells [27], and impact the pro-survival/death pathways [28, 29]. However, the specific role of ADAM17 in determining EC fate and contribution to post-MI tissue repair remains unexplored.

Given the diverse functions of ADAM17, we investigated how endothelial ADAM17 would impact post-MI outcomes. We found that ADAM17 loss severely exacerbated post-MI events by promoting LV rupture through reduced angiogenesis, impaired infarct tissue formation, heightened necroptosis, and increased inflammation. Among these events, blocking inflammatory cell recruitment did not improve post-MI LV rupture, whereas pharmacological inhibition of necroptosis (Nec-1) prevented LV rupture and improved vascular density in the infarcted myocardium. This highlights necroptosis as an important contributor to scar instability and adverse post-MI remodeling.

## Methods

Detailed experimental protocols, reagent lists, and antibody information are provided in Supplementary File 1.

This study was conducted in accordance with the guidelines and regulations approved by the University of Alberta. All animal experiments were performed according to the guidelines outlined in Animal Research: Reporting of in vivo Experiments (ARRIVE), Canadian Council of Animal Care (CCAC), and the NIH Guide for the Care and Use of Laboratory Animals. All studies were approved by the University of Alberta Animal Care and Use Committee (ACUC, AUP 396).

### Mouse strains and endothelial-specific *Adam17* knockdown

*Adam17*^flox/flox^ mice (*Adam17*^f/f^) [30] were cross-bred with *Cdh5-Cre*^*ERT2*^ mice [31] to generate endothelial-specific *Adam17* knockdown (*Adam17*^EC-KD^) and littermate *Adam17*^f/f^ control mice. Mice expressing only Cre-recombinase with intact *Adam17* levels (*Cdh5*-*Cre*^*ERT2*^) and littermate WT mice were also used as controls. *Adam17* knockdown was induced by Tamoxifen injection (100 mg/kg/day, i.p., 5 days) in 10–12-week-old mice, and confirmed at DNA and protein levels (Fig. S2A and B).

### Myocardial infarction surgery and functional analysis

Myocardial infarction was induced by permanent ligation of the left anterior descending (LAD) artery as before [15, 32]. Sham-operated animals served as controls. Cardiac function was assessed by high-resolution echocardiography (Vevo 3100, VisualSonics) [15, 33]. All animal procedures.

### Endothelial cell culture and co-culture with cardiac fibroblasts

Human primary coronary endothelial cells (ECs) were purchased from ATCC (PCS-100-020) and cultured as before [34]. *Adam17*-knockdown was induced by siRNA (EC^*Ad17*-KD^) and confirmed (Fig. S2C). ECs were exposed to normoxia (21% O₂) or hypoxia (1% O₂). EndMT was initiated by exposure to ischemic/profibrotic conditions (hypoxia ± 1 ng/mL TGFβ1) or control (normoxia ± TGFβ1)[35, 36]. Necroptosis was inhibited in vitro by necrostatin-1 (Nec-1; 25 μM).

Primary adult mouse cardiac fibroblasts were isolated, activated to myofibroblasts (MyoFBs) with TGFβ1 (1 ng/mL, 24 h), and co-cultured with ECs (EC^WT^ or EC^*Ad17*-KD^), in control or ischemic/profibrotic conditions (hypoxia + TGFβ1) as before [15, 32, 37]. In a different set of experiments, conditioned media from WT or *Adam17*-knockdown ECs (±normoxia or hypoxia) were added to primary cardiac fibroblasts (cFBs) to assess the paracrine effects of hypoxic EC^*Ad17*-KD^ on FB activation.

### Histological and protein analyses, and collagen cross-linking

Formalin-fixed hearts were paraffin-embedded for Masson’s Trichrome staining. OCT-cryopreserved hearts were used for immunofluorescence staining. Western blots were performed on flash-frozen heart tissues from sham or MI (separated into infarct, peri-infarct, and non-infarct), or cell lysates and conditioned media from cultured ECs. Protein band intensity was quantified with ImageQuant TL software and normalized to the corresponding total protein (Pierce™ stain kit). The hydroxyproline assay was used to quantify soluble and insoluble collagen [15, 38].

### Micro CT-scan for 3D coronary imaging, and in vivo heart cell death assay

Microvascular architecture was visualized by Microfil MV-122 perfusion followed by high-resolution micro-CT (MILabs), and vascular volume was quantified using 3D Slicer as before [32]. Cell death in the heart post-MI was assessed by propidium iodide injection in mice 30 min prior to euthanasia (1 mg/mouse, i.v.).

### Single-nucleus RNA sequencing (snRNA-seq)

Nuclei were isolated from sham, infarct, and peri-infarct LV tissue at day 3 post-MI (*n* = 3 mice per condition, pooled). Libraries were prepared with 10x Genomics v3.1 and sequenced on Illumina NovaSeq X (~400 M reads/sample). Raw data were processed with Cell Ranger [39], ambient RNA removed with CellBender [40], and downstream analyses performed in Seurat (v5). Low-quality nuclei (<500 genes, >20,000 genes, or >5% mitochondrial reads) were excluded. Batch correction was performed with harmony. Cell types were annotated based on canonical markers (e.g., CMs: *Myh7*, *Ryr2*; ECs: *Pecam1*, *Cdh5*) [41–43]. Endothelial subclustering identified EC_1-to-EC_4 subpopulations. Functional pathways were identified through differential gene expression analysis using Seurat FindMarkers (adjusted *p* < 0.05), followed by Gene Ontology enrichment using clusterProfiler [44]. Ligand–receptor analyses were performed with NicheNet (v2.0.5) [45]. Publicly available human MI snRNAseq data [41] were used for cross-species comparison.

### Immune cell depletion and necroptosis inhibition in vivo

In vivo depletion of neutrophils was achieved by i.p. injection of anti-Ly6G antibody, and CD8⁺ T cell depletion by anti-CD8α antibody. Depletion was validated by flow cytometry on blood (CD45, Ly6G, CD3, CD8) (Fig. S2D and E). Necroptosis was inhibited in vivo by i.p. injection of necrostatin-1 (nec-1).

### Statistical analysis

Statistical analyses were performed with GraphPad Prism and SPSS. Normality was tested (Shapiro–Wilk test), and comparison between two groups was done by an unpaired two-tailed *t*-test. Comparison among multiple groups was done using two-way ANOVA followed by Bonferroni or Tukey post-hoc test. Survival was assessed by Kaplan–Meier with the log-rank test. Averaged data are presented as mean ± SEM. Statistical significance was considered at *p* < 0.05.

### Ethics approval

All animal procedures were conducted in accordance with institutional guidelines and the Canadian Council on Animal Care (CCAC) and were approved by the University of Alberta Animal Care and Use Committee (ACUC) under Animal Use Protocols #396. Animal studies adhered to the ARRIVE (Animal Research: Reporting of in vivo Experiments) guidelines.

## Results

### Loss of endothelial ADAM17 compromises survival and exacerbates cardiac dysfunction Post-MI

**Fig. 1:**
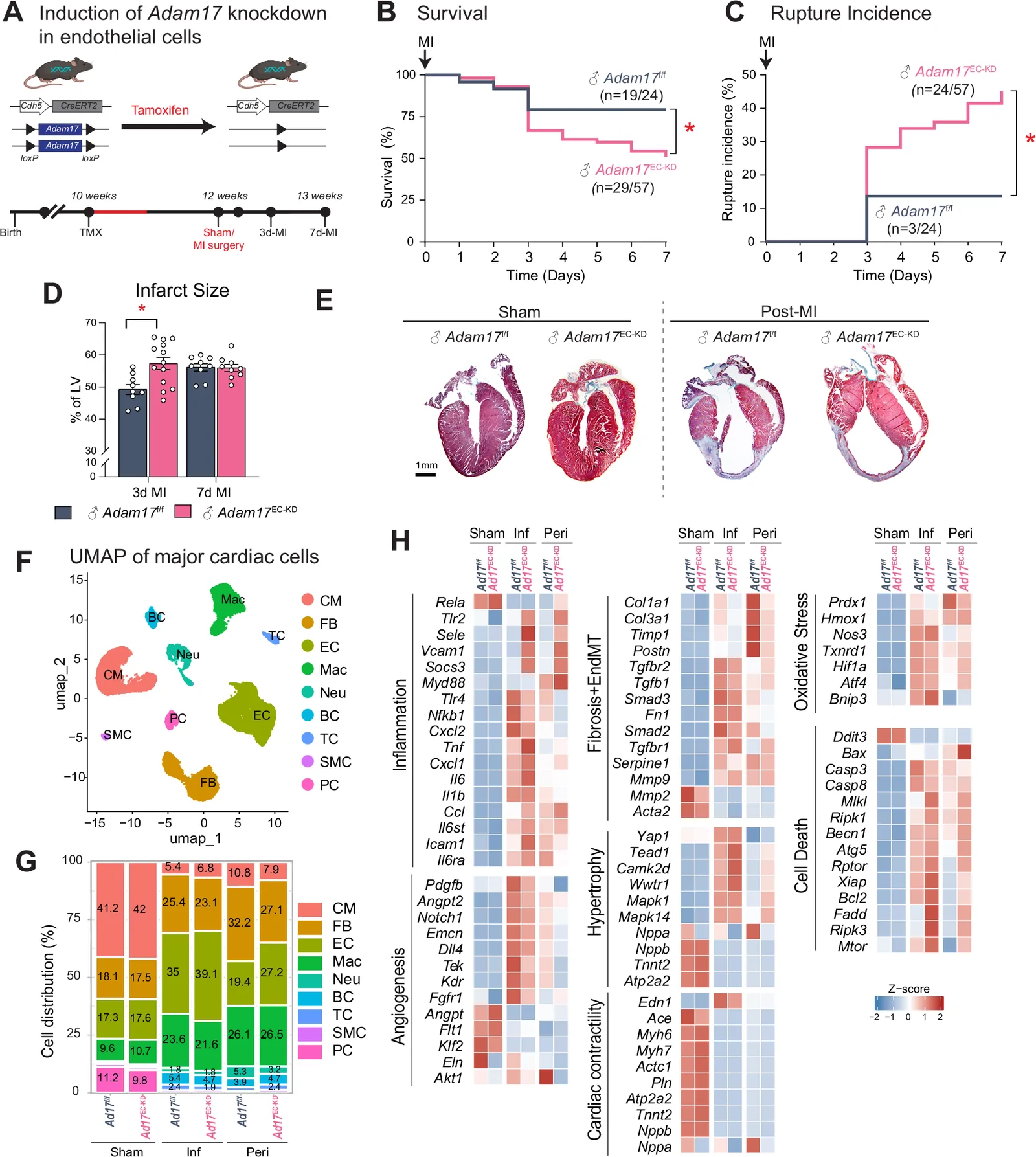
Endothelial ADAM17 knockdown worsens post-MI survival due to increased LV rupture. **A** Schematic of tamoxifen (TMX)-induced ADAM17 knockdown *in Adam17*^f/f^/*Cdh5*-*Cre*^*ERT2*^ (*Adam17*^EC-KD^) mice. Mice received Tamoxifen (i.p., 5 days) at 10 weeks of age, followed by LAD-ligation (or sham) at 12 weeks of age. Kaplan–Meier analysis for post-MI survival (**B**) and LV rupture (**C**) in *Adam17*^f/f^ and *Adam17*^EC-KD^ mice post-MI (Log-rank test). **D** Infarct size measurements at 3 and 7 days post-MI (*n* = 7–12/group). **E** Representative Masson’s trichrome-stained heart sections post-sham or MI from each genotype. UMAP plot (**F**) and cell distribution (**G**) of major cardiac cell populations identified by snRNA-seq from sham, peri-infarct, and infarct regions of *Adam17*^f/f^ and *Adam17*^EC-KD^ hearts. CM cardiomyocyte, FB fibroblast, EC endothelial cell, Mac macrophages, Neu neutrophils, BC B cells, TC cytotoxic T cells, SMC smooth muscle cells, PC pericytes. **H** Heatmap of representative genes for the indicated cellular functions (*z*-score) across sham, infarct, and peri-infarct myocardium. Genes are grouped by the indicated functional categories. Ad17 = *Adam17*, Inf = infarct, Peri = Peri-infarct. Averaged data are presented as mean ± SEM. Statistical analysis was performed using two-way ANOVA with a Bonferroni post hoc test. *p < 0.05 vs. *Adam17*^f/f^.

Following MI, ADAM17 expression increased in the infarct and peri-infarcted myocardium as early 1 day post-MI, with a significant increase in the ECs in the infarct and peri-infarct regions that persists until day 7 post-MI (Fig. S1). ADAM17 knockdown in ECs was confirmed in *Adam17*^EC-KD^ mice at DNA (Fig. S2A) and protein levels (Fig. S2B). Following MI, male *Adam17*^EC-KD^ mice exhibited significantly compromised survival (51% versus 79% in *Adam17*^f/f^ mice) (Fig. 1A, B), which was primarily due to LV rupture that occurred between 3 and 7 days post-MI (Fig. 1C). Echocardiography revealed a significantly greater LV dilation and systolic dysfunction in male *Adam17*^EC-KD^ mice compared to *Adam17*^f/f^ mice, as early as 3 days post-MI, which persisted to day 7 post-MI (Table S1), in addition to the significantly larger infarct size in these mice at 3 days post-MI, but not at day 7 post-MI, which could reflect the higher rupture incidence in the mice with larger infarct size (Fig. 1D, E). TMX-treated *Cdh5*-Cre^ERT2^ mice with wild-type *Adam17* allele expression showed comparable survival and cardiac dysfunction post-MI compared to the littermate WT mice (Fig. S3A, Table S2). Female *Adam17*^EC-KD^ mice exhibited comparable post-MI survival and severity of LV dilation and dysfunction compared to female *Adam17*^f/f^ mice (Fig. S3B, Table S3).

snRNAseq was performed in male *Adam17*^f/f^ and *Adam17*^EC-KD^ mice to detect the myocardial cell populations. UMAP analysis of 52,543 nuclei identified cardiomyocytes (CMs), FBs, ECs, macrophages (Macs), neutrophils (Neu), B cells (BCs), cytotoxic T cells (TCs), smooth muscle cells (SMCs), and pericytes (PCs), classified based on their canonical markers (Fig. 1F and S4A, B). This analysis showed a similar distribution of different cell types (CMs, FBs, ECs, and inflammatory cells) between the two genotypes in the post-sham or post-MI groups. In sham hearts, cardiomyocytes dominated, with ECs, FBs, and Macrophages comprising smaller fractions, whereas post-MI, cardiomyocyte proportions declined sharply, and ECs, FBs, Macrophages, and neutrophils were predominant populations in the infarct and peri-infarct tissue (Fig. 1F, G). Heatmap analysis of molecular markers for key post-MI events, identified by unsupervised GSEA, showed increased markers for inflammation, and decreased markers for ECM remodeling, EndMT, and angiogenesis in post-MI *Adam17*^EC-KD^, while markers for oxidative stress, contractile molecules, and cardiac hypertrophy remained comparable between the genotypes (Fig. 1H).

### Despite the heightened early pro-inflammatory response, inflammatory cell depletion did not improve post-MI survival in *Adam17*^EC-KD^ mice

**Fig. 2:**
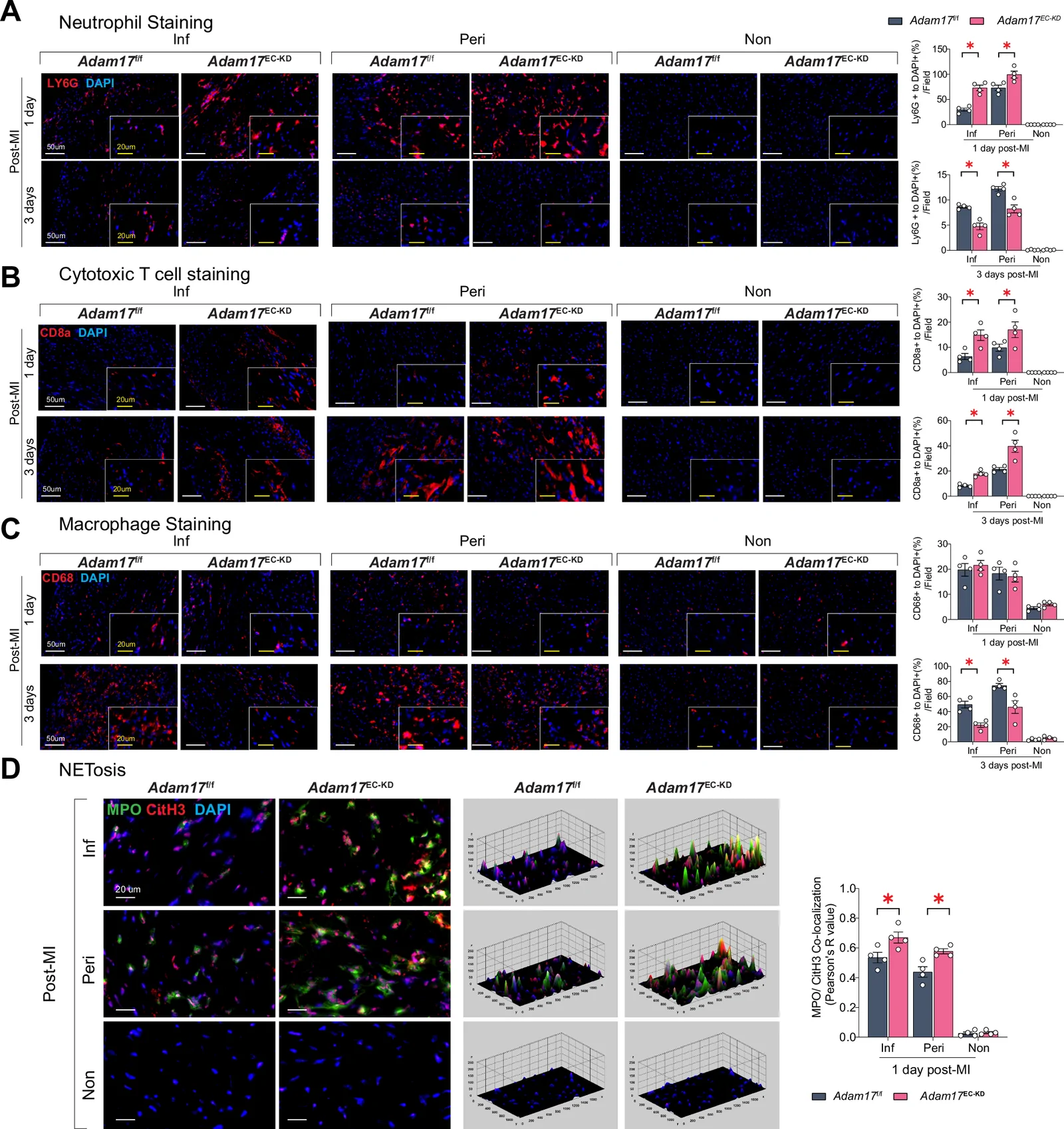
Endothelial ADAM17 deficiency modulates immune cell recruitment and enhances NETosis following MI. Representative immunofluorescence staining and quantification of neutrophils (LY6G, red) (**A**), cytotoxic T cells (CD8a, red) (**B**), and macrophages (CD68, red) (**C**) in the infarct (Inf), peri-infarct (Peri), and non-infarct (non) regions of *Adam17*^f/f^ and *Adam17*^EC-KD^ hearts at post-MI day 1 (top row) and day 3 (bottom row). Nuclei counterstained by DAPI (blue). **D** Representative images of Neutrophil Extracellular Traps (NETs) by co-staining for myeloperoxidase (MPO, green) and citrullinated histone H3 (CitH3, red), at 1 day post-MI. Corresponding surface intensity plots and quantification of MPO/CitH3 colocalization are shown for each genotype. Quantification represents averaged values from 8 to 10 sections/heart, and 4 hearts/group/genotype. Averaged data are presented as mean ± SEM. Statistical analysis was performed using two-way ANOVA with a Bonferroni post hoc test. *p < 0.05 vs. *Adam17*^f/f^.

Inflammation is an early cellular response following MI [46]. Immunofluorescence staining for neutrophils revealed significantly greater infiltration of neutrophils in the infarct and peri-infarct regions of *Adam17*^EC-KD^ at day 1, which subsided by day 3 post-MI (Fig. 2A), whereas the cytotoxic CD8^+^ T-cell population showed a persistent increase compared to *Adam17*^f/f^-MI mice (Fig. 2B). Macrophage abundance was comparable between genotypes at 1 day post-MI, but was significantly reduced on day 3 post-MI in *Adam17*^EC-KD^ compared to *Adam17*^f/f^ mice (Fig. 2C). Comparable abundance of CD4^+^ T cells was observed between genotypes (Fig. S5). The early spike in neutrophil infiltration was associated with enhanced NETosis (neutrophil extracellular trap formation), which has been linked to severe cardiac damage and LV rupture [47], as evident by increased co-localization of myeloperoxidase (MPO) and citrullinated histone H3 (CitH3) in *Adam17*^EC-KD^ mice compared to *Adam17*^*f/f*^ mice (Fig. 2D).

**Fig. 3:**
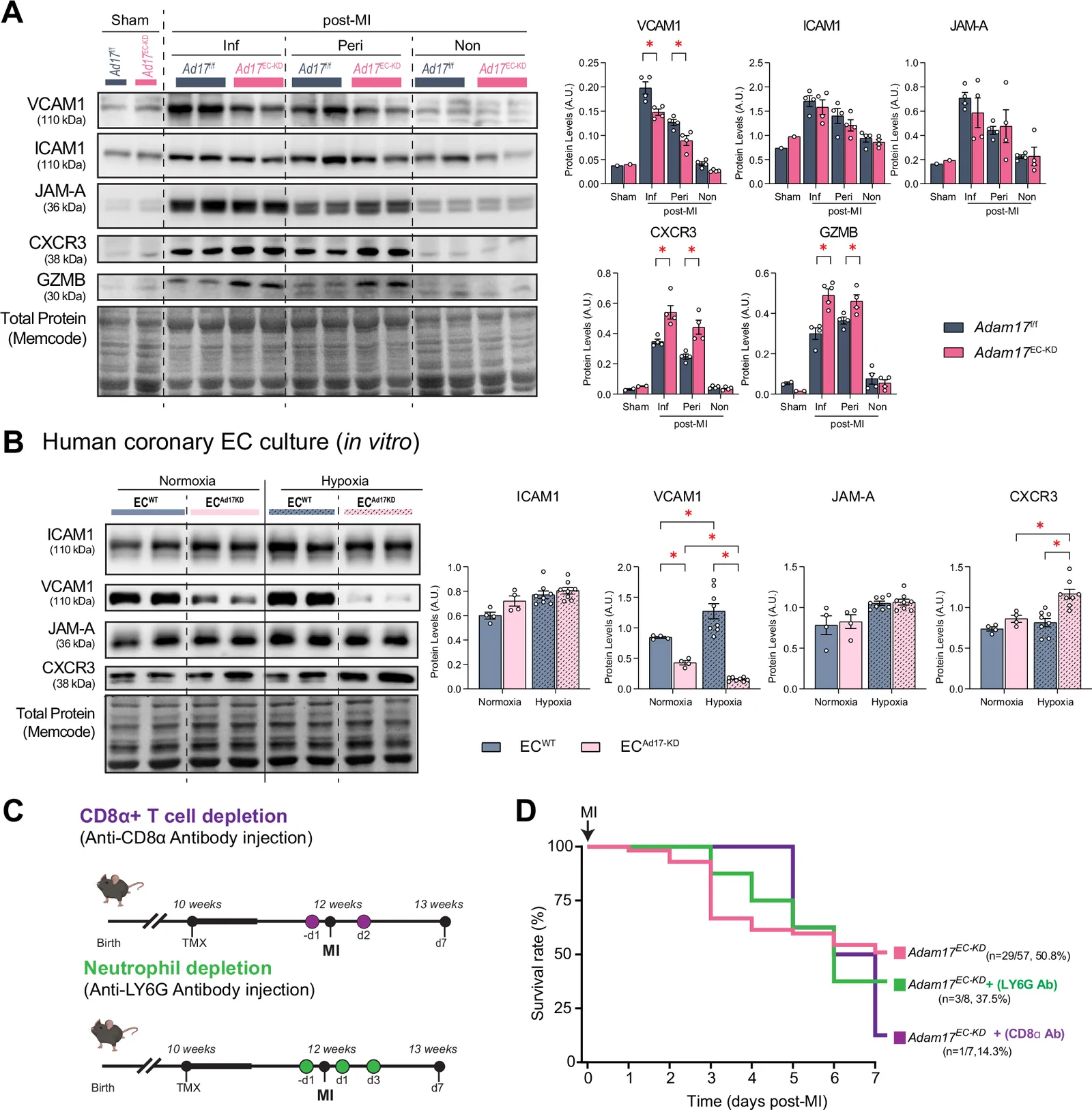
Loss of ADAM17 compromises endothelial cell adhesion, but immune cell depletion does not reduce Post-MI LV rupture incidence. **A** Representative immunoblot and protein quantification of adhesion molecules (ICAM1, VCAM1, JAM-A, CXCR3) and cytotoxic protein granzyme B (GZMB) in infarct (Inf), peri-infarct (Peri), and non-infarct (Non) regions at 3 days post-MI and sham heart tissue in *Adam17*^f/f^ and *Adam17*^EC-KD^ mice. *n* = 4–6 hearts/group/genotype. **B** Representative immunoblot and protein quantification of adhesion molecules (ICAM1, VCAM1, JAM-A, CXCR3) in human coronary endothelial cells (EC), with intact Adam17 levels (scrambled siRNA, EC^WT^) or siRNA-mediated *Adam17* knockdown (EC^*Ad17*-KD^), under normoxia (21% O₂) or hypoxia (1% O₂) conditions (*n* = 3 independent EC cultures, total of 6 wells/group). **C** Schematic illustration of antibody administration timeline for depletion of neutrophils (anti-LY6G) or cytotoxic CD8+ T cells (anti-CD8a) in *Adam17*^EC-KD^ mice post-MI. The colored circles show the days when the antibodies were injected. **D** Kaplan–Meier post-MI survival curves for *Adam17*^EC-KD^ mice following neutrophil (green) or cytotoxic T-cell depletion (purple) compared to no treatment control (pink). Averaged data are presented as mean ± SEM. AU = Arbitrary Units. Statistical analyses were performed using two-way ANOVA with the Bonferroni post-hoc test. *p < 0.05.

Analysis of key immune recruitment molecules showed a significant reduction in VCAM1, but not ICAM1 or JAM-A, while CXCR3, a chemokine receptor that mediates T-cell recruitment and trafficking, was upregulated in the infarct and peri-infarct regions of *Adam17*^EC-KD^ compared to *Adam17*^f/f^ hearts (Fig. 3A). Granzyme B (GZMB), a cytotoxic effector secreted by CD8^+^ T-cells that contributes to adverse remodeling following MI [48], was also significantly increased in *Adam17*^EC-KD^ hearts (Fig. 3A). Using in vitro EC culture, in normoxic and hypoxic conditions to simulate the post-MI conditions, we confirmed that consistent with our in vivo findings, VCAM1 expression was reduced, while CXCR3 expression was significantly increased in EC^*Ad17*-KD^ compared to EC^WT^, but ICAM-1 and JAM-A remained comparable among groups (Fig. 3B).

To determine the causal impact of early and significant increases in infiltration of neutrophils and CD8^+^ T-cells on the increased post-MI LV rupture incidence in *Adam17*^EC-KD^ mice, we selectively depleted neutrophils or CD8^+^ T cells, using specific antibodies, post-MI (Fig. 3C). However, neither neutrophil depletion nor CD8^+^ T-cell depletion improved the post-MI survival in *Adam17*^EC-KD^ mice (Fig. 3D). These data negate the causal role of neutrophil- or CD8^+^ T-cell-driven cytotoxicity in the higher rate of post-MI LV rupture in *Adam17*^EC-KD^ mice.

### Reduced vascular density and impaired angiogenic signaling in mice with endothelial *Adam17* knockdown

**Fig. 4:**
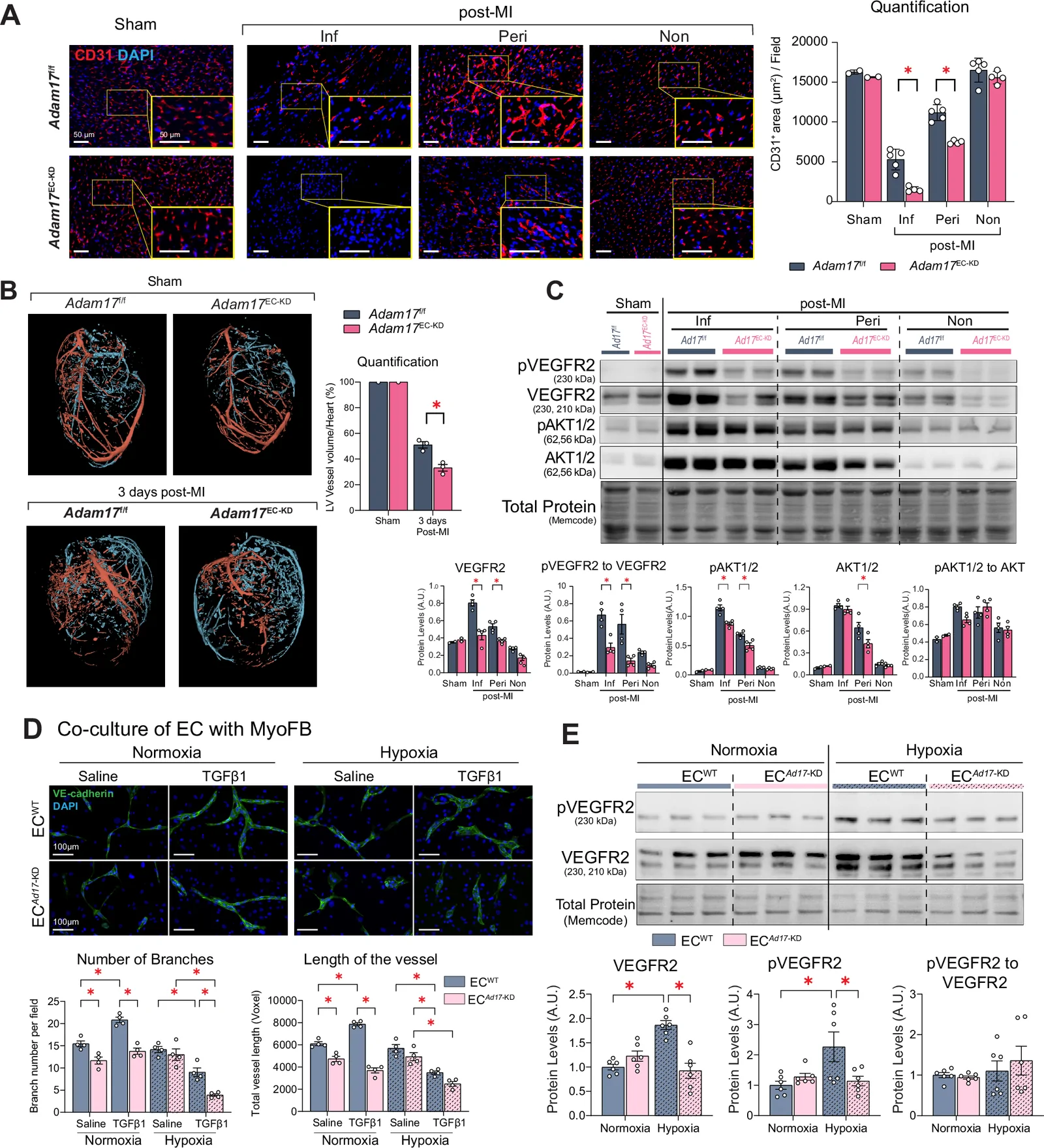
Endothelial ADAM17 deficiency impairs endothelial cell number and VEGFR2 signaling after MI. **A** Representative CD31 immunofluorescence (red) and DAPI nuclear staining (blue) in sham, regions of *Adam17*^f/f^ and *Adam17*^EC-KD^ hearts at 3 days post-MI or Sham, and corresponding quantification (*n* = 3–6 hearts/group; 8–10 sections per heart). **B** Representative micro-CT reconstructions of coronary vasculature and averaged quantification of vessel volume relative to LV volume in sham and post-MI day 3 or Sham hearts (Red=coronaries in anterior LV wall, blue=other coronaries). **C** Representative immunoblot and quantification for phosphorylated and total levels of VEGFR and AKT1/2 post-MI/ sham in indicated groups genotype (*n* = 4–6/group). **D** Co-culture of human coronary endothelial cells with intact Adam17 (scrambled siRNA, EC^WT^) or siRNA-mediated Adam17 knockdown (EC^*Ad17*-KD^) with wildtype cardiac myofibroblasts (intact *Adam17* expression) under normoxia or hypoxia (±TGF-β to simulate the pro-fibrotic and hypoxic infarct environment). Number of branches and total length of tubular structures formed by ECs were quantified (*n* = 4 independent experiments, 3 wells/experiment). **E** Representative immunoblot and averaged quantification of phospho- and total VEGFR2 in EC^WT^ and EC^*Ad17*-KD^ (±hypoxia) (*n* = 3–4/group). Inf = infarct, Peri = peri-infarct, non = non-infarct. AU = Arbitrary Units. Data are presented as mean ± SEM. Statistical analysis by two-way ANOVA with Bonferroni post hoc test. *p < 0.05 in pairwise comparison.

Coronary vascularization is critical in re-establishing perfusion following MI [11, 49]. We found that the endothelial cell population, assessed from CD31-positive staining, was reduced in the infarct and peri-infarct region of *Adam17*^EC-KD^ at day 3 post-MI (Fig. 4A), and in the infarct region at day 7 post-MI (Fig. S6A) compared to *Adam17*^f/f^-MI mice. Consistently, 3-dimensional visualization of functional coronary arteries by micro-CT scan demonstrated markedly suppressed vascular network in the infarct region of *Adam17*^EC-KD^ compared to *Adam17*^f/f^ hearts (Fig. 4B). We next examined the key molecules involved in angiogenic signaling. *Adam17*^EC-KD^ hearts showed a marked reduction in phosphorylated and total VEGFR2 levels, and phospho-to-total AKT1/2 ratio in the infarct and peri-infarct regions (Fig. 4C). However, NOTCH1 and its cleaved products (NEXT and NICD) remained comparable between genotypes (Fig. S6B). Mice that survived to day 7 post-MI showed no differences in these signaling pathways between genotypes (Fig. S6C), indicating that the early impaired angiogenic signaling in *Adam17*^EC-KD^ mice could underlie the higher rate of post-MI LV rupture in these mice.

To determine the direct impact of *Adam17* knockdown on EC function and vascularization potential, we used an in vitro co-culture system of coronary ECs, with intact or knockdown ADAM17 (EC^WT^ or EC^*Ad17*-KD^) and primary cardiac myofibroblasts (normal ADAM17 levels), in hypoxia + TGFβ1 to simulate the hypoxic and pro-fibrotic environment in the infarct tissue. EC^*Ad17*-KD^ showed significantly impaired formation of vessel-like structures and reduced branching, particularly in hypoxic profibrotic conditions (Fig. 4D), along with suppressed activation of VEGFR2 in EC^*Ad17*-KD^ compared to controls (Fig. 4E).

### snRNAseq revealed endothelial ADAM17 deficiency promotes anti-angiogenic, proinflammatory, and anti-fibrotic responses

snRNAseq analysis of other ADAMs that are expressed in the heart or have been linked to heart disease (*Adam8, Adam9, Adam10, Adam12, Adam15, Adam19*) confirmed the loss of *Adam17* in the ECs, but not in other cell types, in *Adam17*^EC-KD^ hearts, while transcription of other *Adam*s was comparable between genotypes (Fig. S4C), indicating that loss of endothelial *Adam17* did not trigger compensatory upregulation of other ADAMs in the heart cells.

**Fig. 5:**
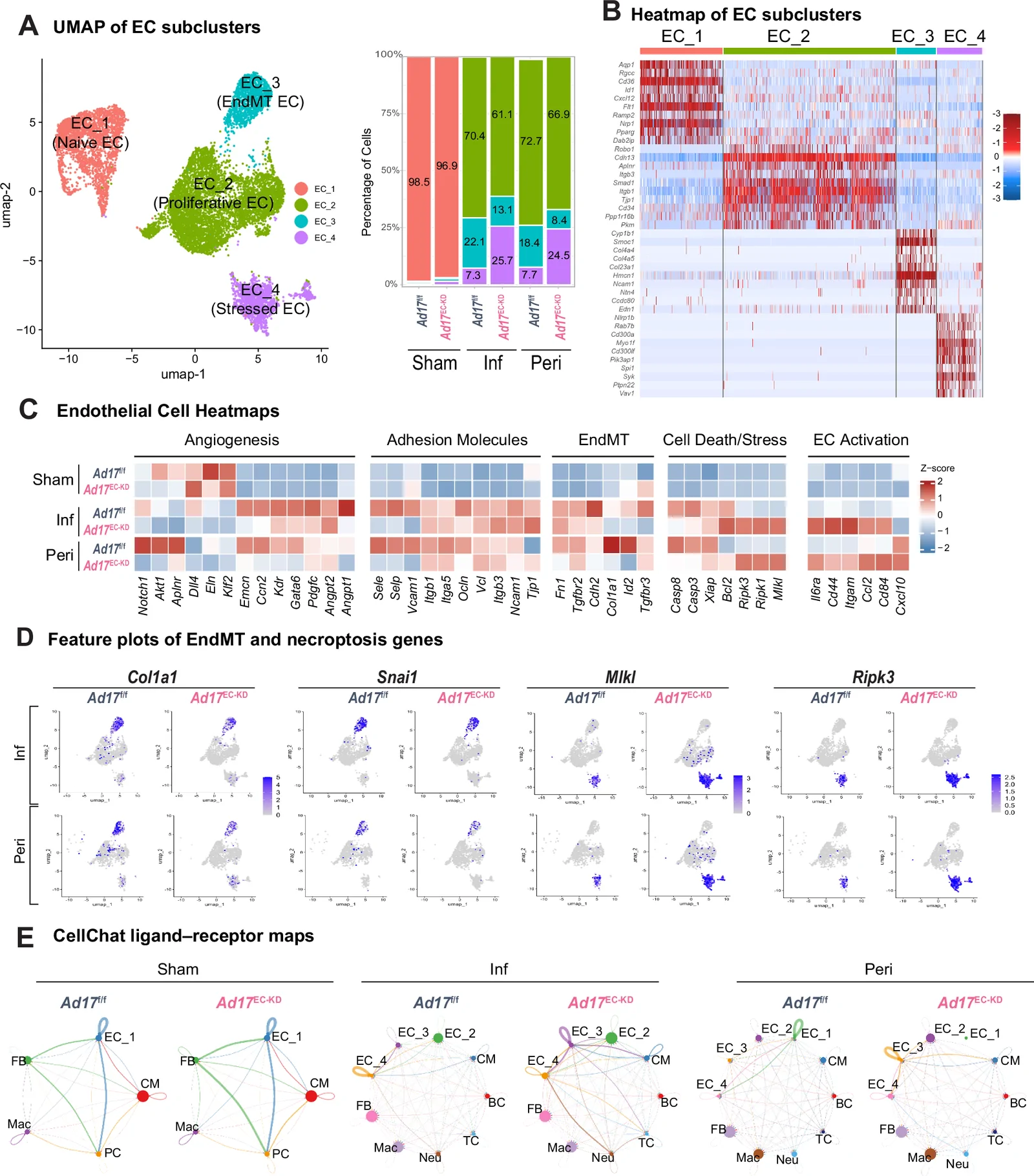
Single-nucleus RNA sequencing reveals impaired angiogenesis, reduced EndMT, and enhanced endothelial cell death signatures in *Adam17*^EC-KD^ hearts post-MI. **A** UMAP plot of endothelial subclusters (EC_1: naïve, EC_2: proliferative/angiogenic, EC_3: EndMT, EC_4: stressed) with proportional distribution across conditions. **B** Heatmap of differentially expressed genes defining each EC subcluster. **C** Heatmaps of genes in ECs corresponding to angiogenesis, adhesion signatures, EndMT, and (early) cell death/stress signatures in indicated groups of either genotype. **D** Feature plots of representative EndMT (*Col1α1*, *Snai1*) and necroptosis-related (*Mlkl*, *Ripk3*) genes in peri-infarct and infarct ECs. **E** CellChat analysis of endothelial cell subclusters (EC_1 to EC_4) with major cardiac cells (CM cardiomyocyte, FB fibroblast, Mac macrophage, Neu neutrophil, BC B cell, TC cytotoxic T cell, and PC pericyte). Ad17 = *Adam17*, Inf = infarct, peri = peri-infarct.

Unbiased subclustering of ECs (*n* = 12,925) identified four transcriptionally distinct subsets (EC_1, EC_2, EC_3 and EC_4) (Fig. 5A). EC_1 subcluster with homeostatic signatures, EC_2 subcluster enriched for angiogenic proteins (*Kdr, Notch1, Angpt2, Angpt1, Dll4*), EC_3 associated with cardiac development and EndMT-like features (*Fn1, Col1a1, Id2, Col1a2, Col5a1*), and EC_4 defined by expression of necroptosis- and inflammation-related genes (*Mlkl, Ripk1, Ripk3, Tnfrsf1a, Il6ra, Cd44, Ccl2*) (Fig. 5B, S7A, B). In *Adam17*^EC-KD^-MI samples, the proportions of EC_2 and EC_3 subclusters were reduced in both the infarct (EC_2: 61.1% vs. 70.4%; EC_3: 13.1% vs. 22.1%) and peri-infarct (EC_2: 66.9% vs. 72.7%; EC_3: 8.4% vs. 18.4%) regions compared with *Adam17*^f/f^ controls, while the EC_4 population was markedly expanded (infarct: 25.7% vs. 7.3%; peri-infarct: 24.5% vs. 7.7%) (Fig. 5A), highlighting a shift from angiogenic and reparative programs toward stressed and inflammatory states.

Differential gene expression and Gene Ontology pathway analysis of ECs in *Adam17*^EC-KD^-MI compared to *Adam17*^f/f^-MI hearts revealed marked suppression of angiogenesis-related pathways, including EC proliferation, differentiation, and migration (Fig. 5C and S7C, D). Key regulators of angiogenic signaling (*Notch1*, *Kdr/Vegfr2*) and EndMT (*Fn1*, *Col1a1*, *Tgfbr3*) were significantly downregulated (Fig. 5C, D), whereas pathways associated with cell death, including granzyme-mediated apoptosis, DNA damage response, and necroptosis, were upregulated, with elevated expression of *Bcl2*, *Ripk1*, *Ripk3*, and *Mlkl* (Fig. 5C, D and S7C, D) in *Adam17*^EC-KD^-MI hearts. CellChat network analysis revealed distinct ligand–receptor communication patterns between EC subclusters and other cell types, such as increased interactions with inflammatory populations (neutrophils, cytotoxic T-cells) in *Adam17*^EC-KD^ compared to *Adam17*^f/f^ (Fig. 5E). These results indicate that loss of endothelial ADAM17 not only impacts the function of ECs but also shifts ECs towards maladaptive immune interactions that could further destabilize the infarct environment.

### Endothelial ADAM17 deficiency impairs scar stabilization through defective EndMT

**Fig. 6:**
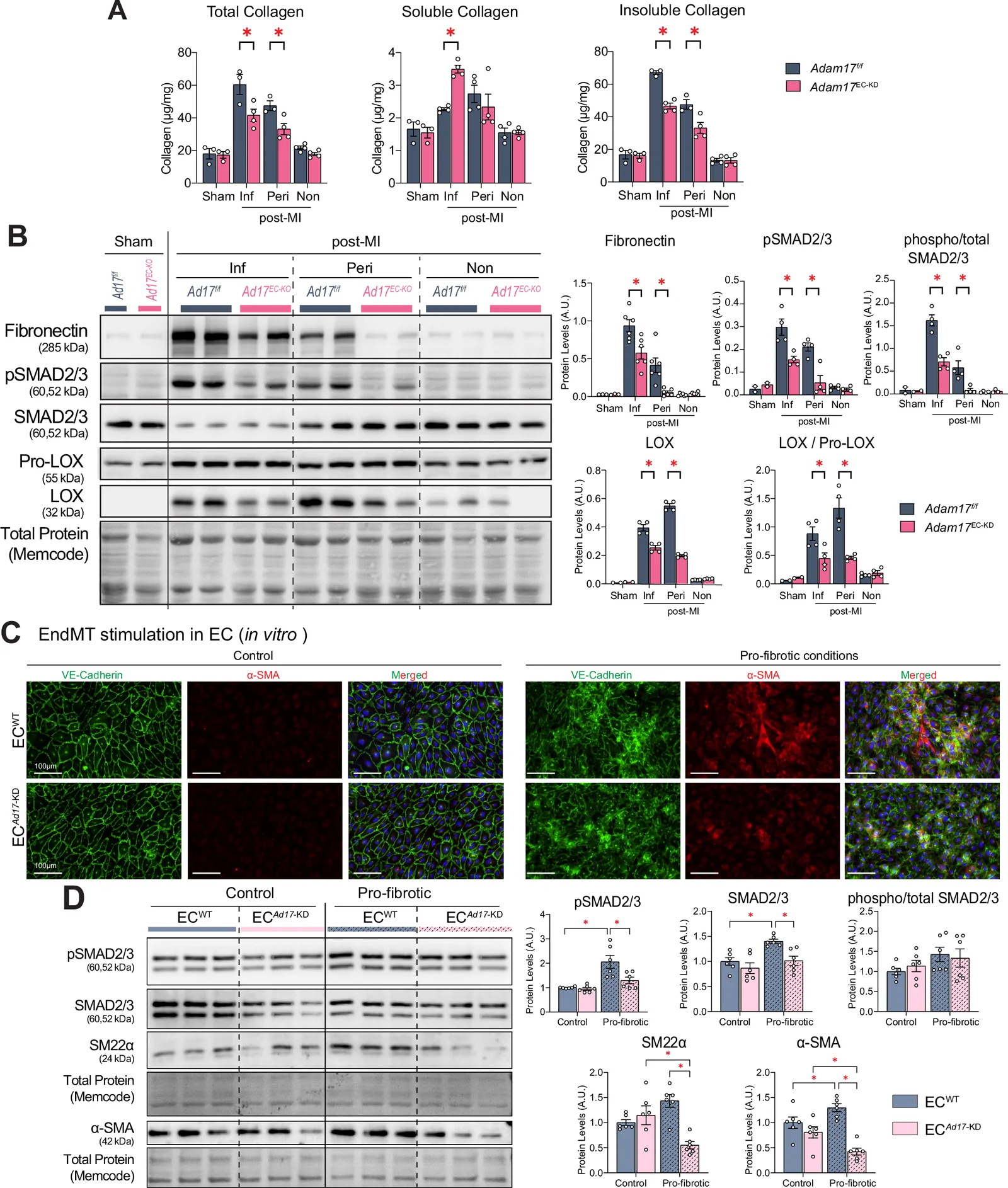
Endothelial ADAM17 deficiency impairs scar stabilization and endothelial-to-mesenchymal transition (EndMT). **A** Total, soluble, and insoluble collagen content (hydroxyproline assay) in sham or 3 days post-MI in the indicated groups. **B** Representative immunoblot and protein quantification of extracellular matrix protein, fibronectin, and related signaling molecules in *Adam17*^f/f^ and *Adam17*^EC-KD^ hearts at 3 days post-MI (*n* = 4–6 hearts/group). **C** Representative immunofluorescence staining for αSMA (red, marker of fibroblast-like characteristics); VE-Cadherin (green, EC protein) and DAPI (blue, Nuclear staining) in human coronary endothelial cells, with intact (EC^WT^) or *Adam17*-knockdown (EC^*Ad17*-KD^) in control or pro-fibrotic/hypoxia conditions (similar to infarct environment) to induce EndMT. **D** Representative immunoblot and protein quantification in EC^WT^ and EC^*Ad17*-KD^ in control or pro-fibrotic/hypoxic conditions (*n* = 3 independent experiments). SM22α smooth muscle protein (transgelin), αSMA alpha smooth muscle actin, LOX lysyl oxidase. Averaged data are presented as mean ± SEM. Statistical comparisons were performed using two-way ANOVA with a Bonferroni post hoc test. *p < 0.05.

To further identify the underlying mechanism for increased LV rupture in *Adam17*^EC-KD^-MI mice, we investigated the integrity of the infarct tissue. Hydroxyproline assay showed that total and insoluble (cross-linked) collagen were significantly reduced in the infarct and peri-infarct myocardium in *Adam17*^EC-KD^ compared to *Adam17*^f/f^ hearts (Fig. 6A). Notably, in mice that survived to day 7 post-MI, total collagen content and crosslinking were comparable between genotypes (Fig. S8A). Heatmap for FB populations showed a comparable profile in the sham hearts from either genotype, but decreased expression of the genes linked to ECM production and remodeling, and FB activation to myofibroblasts, were detected in the peri- and infarct regions of *Adam17*^EC-KD^ hearts (Fig. S8B). In addition, fibronectin, lysyl oxidase (LOX), and activation of the pro-fibrotic SMAD2/3 pathway were decreased in the infarct and peri-infarct regions of *Adam17*^EC-KD^ compared to *Adam17*^f/f^ hearts at 3 days post-MI (Fig. 6B), but not at 7 days post-MI (Fig. S8C). These data support the notion that impaired infarct tissue deposition and stabilization in *Adam17*^EC-KD^-MI hearts predisposed them to a greater risk of rupture.

Transformation of ECs to FB-like cells (EndMT) allows them to contribute to infarct formation by synthesizing ECM proteins [50]. SnRNAseq data showed suppressed EndMT-related pathways in the EC populations from *Adam17*^EC-KD^-MI hearts, with downregulation of canonical EndMT markers (*Snai1*, *Col1a1*, *Col1a2*, *Fn1*, *Tgfbr2*) and reduced representation of the EndMT-enriched EC_3 subcluster. Primary culture of Human coronary ECs exhibited EndMT when subjected to hypoxic-profibrotic conditions, detected by expression of αSMA (alpha smooth muscle actin), a marker of FB activation. In contrast, EC^*Ad17*-KD^ exhibited markedly suppressed αSMA expression **(**Fig. 6C**)**, as well as reduced SM22α levels and pro-fibrotic signaling, phosphoSMAD2/3, compared to EC^WT^ in hypoxic-profibrotic conditions (Fig. 6D). In addition, the conditioned media from EC^WT^ or EC^*Ad17*-KD^ in normoxic or hypoxic conditions, when added to primary cardiac FBs, showed that the conditioned media from hypoxic EC^WT^ triggered FB activation, but the parallel conditioned media from EC^*Ad17*-KD^ did not activate the FBs (Fig. S9). These data demonstrate a critical role for endothelial ADAM17 in mediating EndMT in ECs and in activating the FBs through a paracrine mechanism.

### Endothelial ADAM17 loss promotes necroptosis following myocardial infarction

**Fig. 7:**
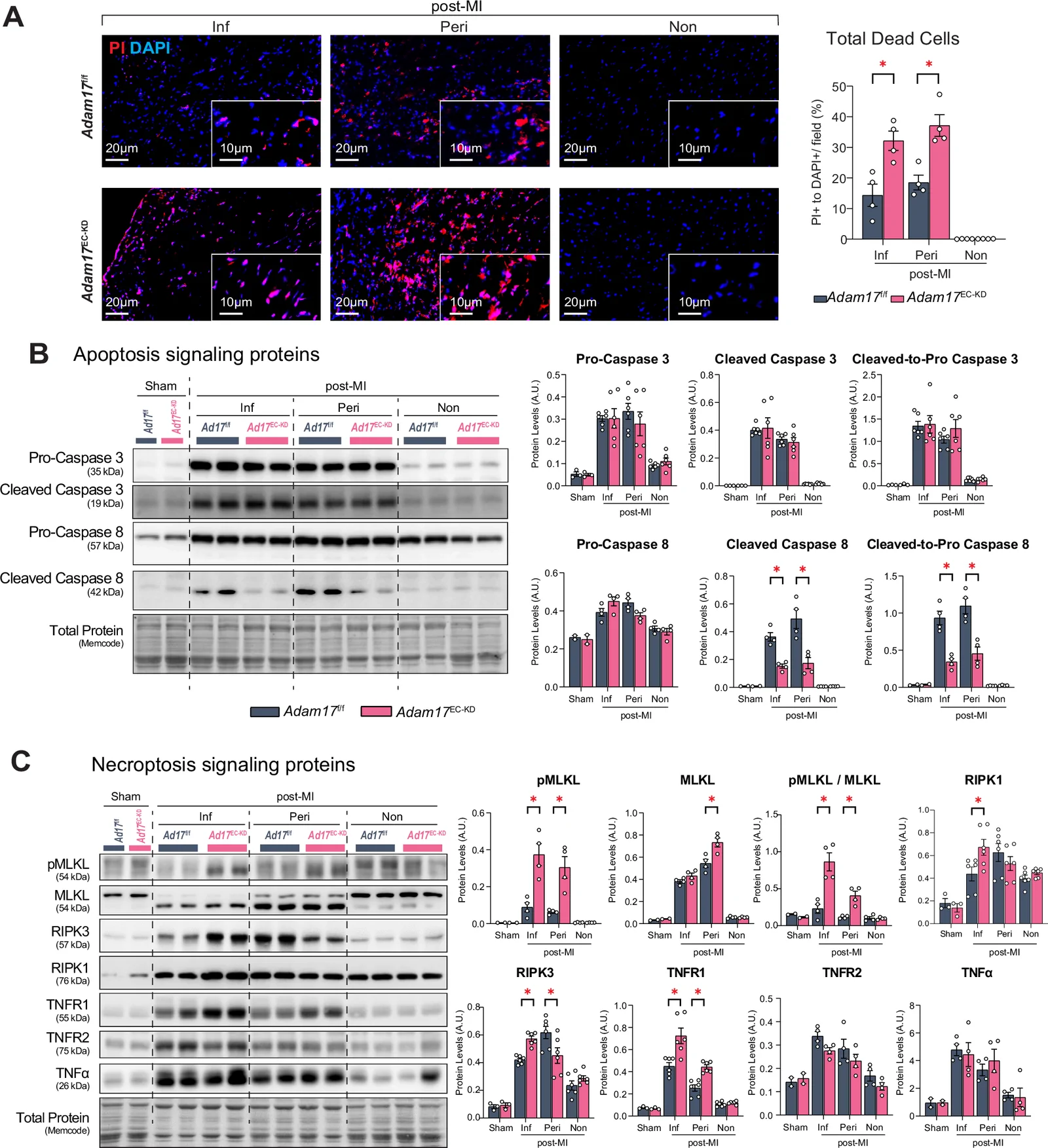
Endothelial ADAM17 deficiency promotes necroptotic cell death and suppresses apoptotic signaling following MI. **A** Representative immunofluorescent co-staining and quantification of dead/dying cells (Propidium Iodide, PI, red; DAPI, blue) in infarct (Inf), peri-infarct (Peri), and non-infarct (Non) regions at 3days post-MI (*n* = 4 hearts/group/genotype). **B** Representative immunoblot and protein quantification for pro-apoptosis (Caspase 3) and anti-apoptosis (Caspase 8) in indicated groups (*n* = 4–6 hearts/group/genotype). **C** Representative immunoblot and protein quantification for necroptosis-related proteins: Mixed Lineage Kinase Domain-Like (MLKL), receptor-interacting serine/threonine kinases (RIPK1, RIPK3), Tumor necrosis factor receptors (TNFR1, TNFR2), and Tumor necrosis factor-alpha (TNFα) at 3 days post-MI or sham in each genotype (*n* = 4–6 hearts/group/genotype). Averaged data are presented as mean ± SEM. Statistical analyses were performed using two-way ANOVA with a Bonferroni post hoc test. *p < 0.05 vs. *Adam17*^f/f^. *Ad17*^f/f^ = *Adam17*^f/f^ and *Ad17*^EC-KD^ = *Adam17*^EC-KD^.

SnRNAseq analyses revealed a strong enrichment of genes associated with cell death pathways in *Adam17*^EC-KD^ hearts (Fig. 5C), which would primarily include cells at the early stages of cell death (dead cells are selectively filtered and excluded from snRNAseq analyses). General cell death was assessed in vivo on day 3 post-MI (by PI), showing a markedly greater number of PI-positive cells in the infarct and peri-infarct regions of *Adam17*^EC-KD^ compared to *Adam17*^f/f^ heart (Fig. 7A). SnRNAseq data showed a downregulation of apoptosis-related genes (*Casp3, Casp8*), but an enrichment of necroptosis-associated transcripts (*Mlkl, Ripk1*, and *Ripk3*), in ECs from *Adam17*^EC-KD^-MI (Fig. 5D). Cleaved Caspase-3, a marker of apoptosis, was comparable between genotypes, whereas cleaved Caspase-8, a negative regulator of necroptosis [51], was significantly reduced in infarct and peri-infarct regions of *Adam17*^EC-KD^ hearts (Fig. 7B). Further assessment of the signaling pathways associated with necroptosis showed a significant increase in RIPK1, RIPK3, phosphorylated MLKL (pMLKL), and TNFR1, in the infarct and peri-infarct regions of *Adam17*^EC-KD^ compared to *Adam17*^f/f^ hearts, while TNFR2 and TNFα were comparable between genotypes (Fig. 7C).

**Fig. 8:**
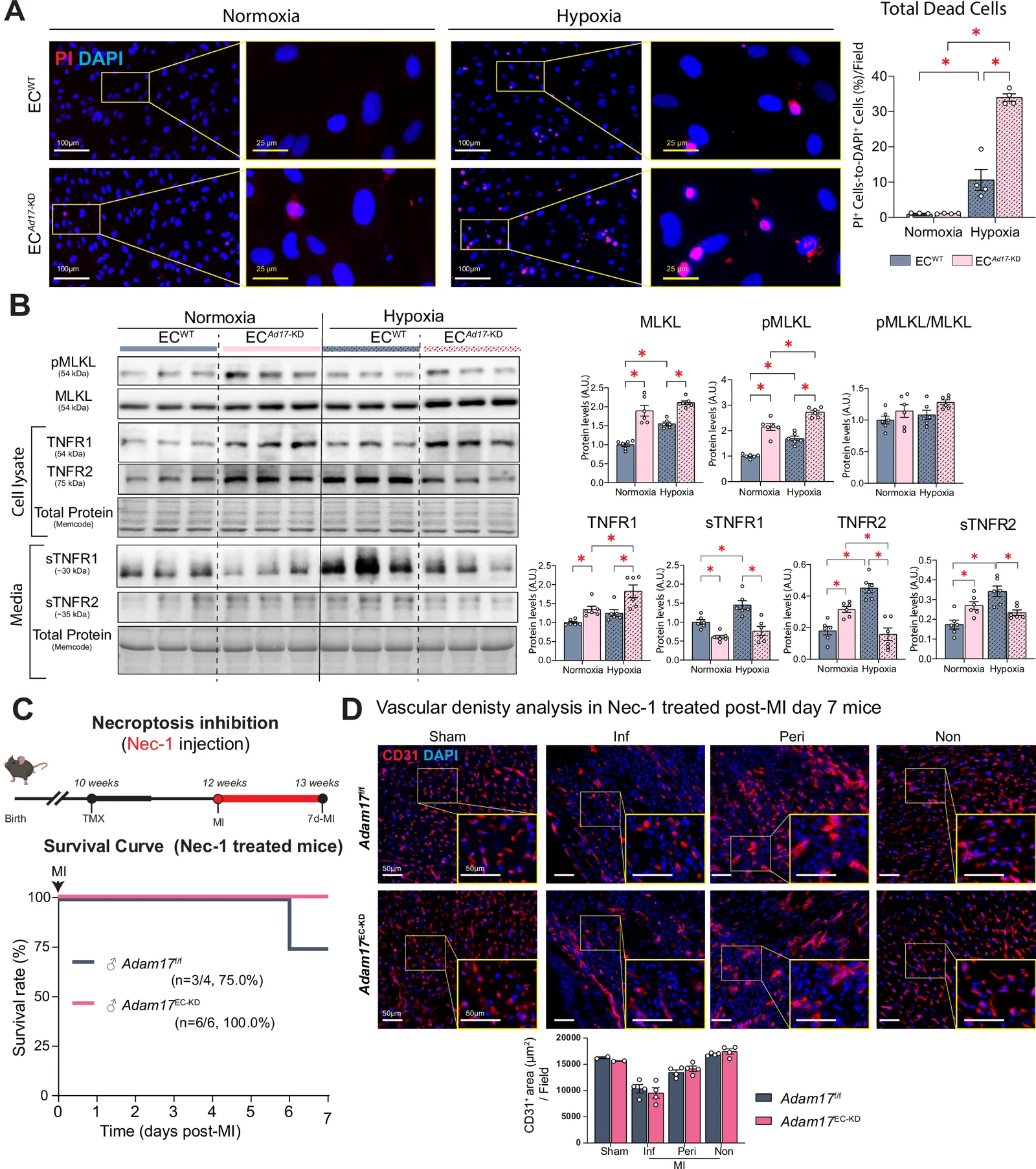
Endothelial ADAM17 loss reduces TNFR1 shedding and heightens necroptosis, while necroptosis inhibition prevents post-MI rupture and restores the endothelial cell population in *Adam17*^EC-KD^ mice. **A** Representative immunofluorescence images and quantification of propidium iodide (PI, red) and DAPI (blue) staining in endothelial cells (EC^WT^ and EC^*Ad17*-KD^) cultured under normoxia or hypoxia (*n* = 3 independent experiments; 6–8 fields per condition). **B** Representative immunoblots and protein quantification for Mixed Lineage Kinase Domain Like (MLKL and pMLKL), and Tumor necrosis factor receptor 1 (TNFR1) and receptor 2 (TNFR2), soluble (in conditioned media) and membrane-bound (in cell lysate), in ECs with intact *Adam17* (EC^WT^) or with *Adam17* knockdown (EC^*Ad17*-KD^). **C** Schematic of treatment of mice with a necroptosis inhibitor, Necrostatin-1 (Nec-1), and post-MI survival curve showing that pharmacological inhibition of necroptosis improved post-MI survival in *Adam17*^EC-KD^ mice by preventing LV rupture. **D** CD31 (red) immunostaining at day 7 post-MI in Nec-1–treated mice shows reversal of the drastic decrease in coronary density in *Adam17*^EC-KD^-MI compared to *Adam17*^f/f^-MI hearts in the infarct, peri-infarct, and non-infarct regions (*n* = 3–4 hearts/genotype). Statistical analysis was performed using two-way ANOVA with a Bonferroni post hoc test. *p < 0.05 vs. *Adam17*^f/f^.

Consistent with snRNA-seq analysis showing enrichment of necroptosis-related genes and expansion of the stressed EC_4 endothelial subcluster in *Adam17*^EC-KD^ hearts, co-immunostaining for pMLKL and ECs showed a significantly higher number of ECs in the infarct region of *Adam17*^EC-KD^ mice with activated MLKL signaling, indicating greater necroptosis in the ECs of *Adam17*^EC-KD^-MI hearts (Fig. S10). The direct impact of ADAM17 loss on susceptibility of ECs to necroptosis was confirmed in vitro as the higher hypoxia-mediated cell death in EC^*Ad17*-KD^ (Fig. 8A) was associated with increased levels of MLKL and its phosphorylated form (pMLKL), a well-known marker of necroptosis [52] compared to parallel EC^WT^ (Fig. 8B). Inhibition of necroptosis (by necrostatin-1) reduced hypoxia-induced cell death (Fig. S11A) and MLKL phosphorylation in ECs in vitro (Fig. S11B). Interestingly, inhibition of necroptosis in vitro also attenuated EndMT signaling and expression of mesenchymal markers (pSMAD2/3, αSMA, and SM22α) under pro-fibrotic conditions in both EC^WT^ and EC^*Ad17*-KD^ (Fig. S12), suggesting that necroptosis signaling also modulates EndMT-associated pathways in ECs. In vivo, hypoxia-mediated shedding of TNFR1 (into the conditioned media) was significantly decreased in EC^Ad17-KD^ compared to EC^WT^ (Fig. 8B), consistent with TNFR1 being a well-known substrate of ADAM17 [53, 54]. Interestingly, TNFR2 shedding was not altered in EC^Ad17-KD^ under hypoxic conditions (Fig. 8B), demonstrating a selective function of ADAM17 sheddase activity towards TNFR1 in ECs, and its contribution to activation of the downstream necroptosis pathway. We further demonstrate that inhibition of necroptosis, by injection of necrostatin-1 post-MI, prevented LV rupture, ameliorated LV dysfunction and dilation (Fig. 8C), and significantly improved the endothelial cell population that could represent coronary vascular density (Fig. 8D) in *Adam17*^EC-KD^ mice.

## Discussion

Myocardial recovery from ischemic injury is a complex and time-dependent interplay of the cells in the heart. The relative contribution of fibroblast-driven scar formation [55] and inflammatory cell recruitment [56] on post-MI outcome has been explored. ADAM17 levels are increased after MI in patients [20–22] and in animal models [57], and its role in cardiomyocytes [18, 58] and fibroblasts [32] has been studied. Endothelial cells are primarily known for their role in vascularization. Here, we report endothelial ADAM17 as a key regulator for the critical role of ECs in optimal post-MI recovery. EC-specific *Adam17*-knockdown, in vivo and in vitro, uncovered novel functions of ADAM17 in ECs in post-MI remodeling and survival, including inflammation, VEGFR2-mediated vascularization, ECM formation and assembly (via EndMT), and TNFR1-mediated necroptosis. Our findings highlight the role of ECs and that their fate can drastically affect the stability of the infarcted myocardium.

ECs play a pivotal role in the conditions of the infarct microenvironment by orchestrating angiogenesis and modulating inflammation [59]. Coronary density and VEGFR2-mediated signaling, including AKT1/2, the downstream effector of VEGFR2 that can promote endothelial survival, proliferation, and vessel stability [60], were significantly suppressed in the infarct and peri-infarct regions in *Adam17*^EC-KD^ mice. This is consistent with the reported role of VEGFR2 activity for neo-vascularization and functional recovery after MI [4, 18, 61]. ADAM17-mediated shedding of HB-EGF and transactivation of EGFR, which in turn sustains VEGFR2 transcription [62], could underlie the reduced VEGFR2 protein levels observed in *Adam17*^EC-KD^ hearts. Although endothelial ADAM17 loss also exacerbated the inflammatory response in *Adam17*^EC-KD^-MI hearts, immune cell depletion revealed that neutrophil and CD8⁺ T cell-mediated inflammation is not the primary driver of post-MI LV rupture in this model, indicating a predominant role for endothelial intrinsic mechanisms in the compromised infarct integrity in *Adam17*^EC-KD^ mice.

This study further identifies a critical role for ADAM17 in regulating EndMT and its contribution to scar formation. While fibroblasts are the principal cell source for ECM, EC-derived fibroblast-like cells, generated via EndMT, provide an essential early source of collagen that stabilizes the infarct scar during wound healing [50]. *Adam17*^EC-KD^-MI hearts displayed depletion of the EndMT-enriched EC_3 cluster, suppressed activation of the SMAD2/3 pathway, which is required for EndMT initiation [63], and failure to undergo TGFβ1-induced EndMT in vitro, indicating that endothelial ADAM17 regulates EndMT post-MI. In addition to regulating endothelial phenotypic transition, our findings show that endothelial ADAM17 also modulates cardiac FB activation through paracrine mechanisms. ECs can secrete mediators such as PDGF and CTGF, which regulate FB activation and ECM deposition during infarct healing [64, 65]. Impaired endothelial signaling in the absence of ADAM17 can therefore limit FB activation and collagen deposition, contributing to defective scar formation and increased susceptibility to ventricular rupture post-MI.

Another novel finding of this study is the role of endothelial ADAM17 in necrosis post-MI. While apoptosis is considered a canonical mode of EC death in cardiovascular diseases, accumulating evidence points to necroptosis as a dominant driver of inflammation-induced vascular injury [66, 67]. Necroptosis mediated through the RIPK1–RIPK3–MLKL pathway is recognized as a dominant form of endothelial demise, amplifying injury through both lytic death and pro-inflammatory signaling [66–68]. In our study, snRNAseq revealed upregulation of necroptosis-related transcripts (*Ripk1, Ripk3, Mlkl*) and downregulation of *Casp8*, an inhibitor of this pathway [51], in *Adam17*^EC-KD^-MI hearts. These transcriptomic changes were validated at the protein level in vivo and in vitro, with hyperactivation of the RIPK1, RIPK3, and pMLKL in *Adam17*^EC-KD^-MI hearts, and in EC^Ad17-KD^ in vitro. This was further linked to cell membrane stabilization of TNFR1, a well-known substrate for ADAM17 sheddase activity [53, 54], consistent with the role of TNFR1 in promoting RIPK1–RIPK3--MLKL-mediated necroptosis in other cell types [69–71]. Rescue of post-MI adverse outcomes in *Adam17*^EC-KD^ by inhibition of necroptosis highlights the role of ADAM17 in controlling endothelial necroptosis as a key regulator of post-MI outcomes. Consistent with our findings, analysis of publicly available single-cell RNA-seq data from human ischemic cardiomyopathy specimens [41] also revealed that EndMT in ECs positively correlated with *Adam17* expression, while EC necroptosis negatively correlated with *Adam17* expression (Fig. S13), supporting that loss of endothelial ADAM17 can reduce EndMT and increase necroptosis, underscoring the translational and clinical relevance of our findings.

Female mice generally develop a smaller infarct, less LV dilation and dysfunction, and no LV rupture post-MI [72]. Interestingly, female *Adam17*^EC-KD^ mice showed comparable post-MI cardiac function but an increase in LV rupture incidence (10%) compared to 0.0% in *Adam17*^f/f^ females. This demonstrates the important role of endothelial ADAM17 in the formation of infarct tissue in both sexes. Nonetheless, it has been reported that necroptosis is sexually dimorphic [73]. Experimental and clinical studies have shown that females exhibit greater resistance to ischemic cardiac injury, which has been partly attributed to cardioprotective effects of estrogen signaling [74, 75]. In addition, females exhibit lower RIPK1-RIPK3-MLKL activation due to hormone-dependent modulations, which may reduce their susceptibility to necroptosis [73, 76], which could underlie the protection observed in female *Adam17*^EC-KD^ mice against LV rupture compared to male *Adam17*^EC-KD^ mice.

Overall, this study uncovers novel functions for ADAM17 as a master regulator of endothelial fate and scar stability after MI. These findings provide new insight into the diverse and distinct cell-specific functions of ADAM17 in the complex process of myocardial recovery post-MI.

## Supplementary information

SUPPLEMENTAL MATERIAL

Original Data

## Data Availability

All data included in this manuscript will be made available upon reasonable request. snRNA sequencing data have been archived at NCBI. The accession number is NCBI: GSE311896. All analyses were performed using standard functions and workflows from the Seurat R package. No custom software or code was developed for this study.

## Code availability

All data included in this manuscript will be made available upon reasonable request. snRNA sequencing data have been archived at NCBI. The accession number is NCBI: GSE311896. All analyses were performed using standard functions and workflows from the Seurat R package. No custom software or code was developed for this study.
