# Supplementary material for: Necroptosis is a key contributor to impaired cardiac repair following myocardial infarction
Source: Cell Death Dis. 2026 Jun 15;17(1):751. doi: 10.1038/s41419-026-08979-5 (PMC13500855; doi:10.1038/s41419-026-08979-5)
Supplement: Supplementary file 1 [file 41419_2026_8979_MOESM1_ESM.pdf]

Necroptosis is more critical than inflammation in the adverse myocardial remodeling following myocardial infarction

Razoan Al Rimon[1], Yingxi Li[1], Ilamaran Meganathan[1], Faqi Wang[2], Allan G. Murray[2], Gavin Y. Oudit[2], Slava Epelman[3], Xavier Clemente-Casares[4], Zamaneh Kassiri[1]

[1] Department of Physiology, Faculty of Medicine and Dentistry, University of Alberta, Edmonton, AB, Canada.

[2]Department of Medicine, Faculty of Medicine and Dentistry, University of Alberta, Edmonton, AB, Canada

[3] Department of Immunology, University of Toronto, Ted Rogers Centre for Heart Failure Research, Peter Munk Cardiac Centre, Toronto, ON, Canada

[4] Department of Medical Microbiology and Immunology, Faculty of Medicine and Dentistry, University of Alberta, Edmonton, AB, Canada

EXPANDED METHODS

Mouse Strains and Tamoxifen-Induced Endothelial-Specific ADAM17 Knockdown
Wild type (WT) and *Adam17*$^{flox/flox}$ (*Adam17*$^{f/f}$) mice were obtained from The Jackson Laboratory (1, 2). *Cdh5-CreERT2* transgenic mice were generated as previously described (3). Endothelial-specific ADAM17 knockdown mice (*Adam17*$^{f/f}$*/Cdh5CreERT2*$^{+/-}$ or *Adam17*$^{EC\text{-}KD}$) were generated by crossing *Adam17*$^{f/f}$ mice with *Cdh5-CreERT2* transgenic mice. Littermate *Adam17*$^{f/f}$ mice lacking Cre recombinase served as controls, along with WT and Cre-recombinase control, *Cdh5-CreERT2* mice. Cre-mediated recombination was activated through tamoxifen (100mg/kg/day) (Sigma, 10540-29-1) administration intraperitoneally for five consecutive days. Injections were performed two weeks prior to myocardial infarction (MI) surgery. Deletion of *Adam17* was confirmed by DNA genotyping and immunofluorescence. Genotyping identified LoxP-flanked alleles in both *Adam17*$^{f/f}$ and *Adam17*$^{EC\text{-}KD}$ mice (1100bp band), with exon 2 deletion (320 bp band) detected only in *Adam17*$^{EC\text{-}KD}$ mice **(Figure S2A)**. Co-immunofluorescence staining of heart tissue sections demonstrated markedly reduced ADAM17 expression in endothelial cells ($CD31^+$ staining) following tamoxifen injection **(Figure S2B)**.

Myocardial Infarction Surgery and Tissue Collection
Myocardial infarction (MI) was induced in 11-12-week-old mice by permanent ligation of the left anterior descending (LAD) coronary artery. Sham-operated mice underwent an identical surgical procedure without LAD ligation and served as controls. Post-operative survival was closely monitored, and cardiac rupture was assessed through autopsy in all deceased mice. At defined time points, hearts were harvested and processed for downstream analyses. Tissues were fixed in 10% neutral-buffered formalin for histological evaluation, embedded in optimal cutting temperature (OCT) compound for immunofluorescent staining; or snap-frozen and dissected into infarct, peri-infarct, and remote zones for molecular studies.

Echocardiography and Myocardial Strain Analysis
Cardiac structure and function were evaluated using noninvasive transthoracic echocardiography in anesthetized mice (1-1.5% isoflurane in oxygen) with a high-resolution Vevo 3100 imaging system (VisualSonics), as previously described (4-6). Standard B-mode and M-mode images were acquired from parasternal long-axis and short-axis views. Left ventricular (LV) parameters including LV anterior wall thickness (LVAW), LV posterior wall thickness (LVPW), end-diastolic diameter (LVEDD), end-systolic diameter (LVESD), and fractional shortening (FS) were measured using M-mode imaging. LV volumes in diastole and systole were calculated from EKV loops using Simpson's method to determine ejection fraction (EF). Global longitudinal and circumferential strain analyses were performed using Vevo LAB software, with strain derived from EKV loops obtained from the parasternal long-axis and short-axis views at the mid-papillary levels. *In vivo* infarct size was estimated from parasternal long-axis views based on regional wall motion abnormalities, as previously reported (7, 8).

In Vitro Culture and ADAM17 Knockdown in Human Aortic Endothelial Cells (HAoECs)

Primary human aortic endothelial cells (HAoECs; ATCC, PCS-100-011) were cultured in vascular cell basal medium (VCBM; ATCC, PCS-100-030) supplemented with an endothelial cell growth kit-BBE (ATCC, PCS-100-040), according to the manufacturer's instructions. Cells were maintained at 37°C in a humidified incubator with 5% CO_2 and 95% air. Endothelial identity and purity were confirmed by characteristic cobblestone morphology and VE-cadherin immunofluorescence staining. For ADAM17 knockdown, HAoECs were transfected with ADAM17-specific siRNA (Ambion, s13720) using Lipofectamine® RNAiMAX transfection reagent (Invitrogen, 13778-150) for 6 hours as per the manufacturer's protocol. A scrambled siRNA (Ambion, Cat. No. 4390844) was used as a negative control. Knockdown efficiency was confirmed by Western blot analysis **(Figure S2C)**. Following siRNA transfection, HAoECs were serum-starved in vascular cell basal medium supplemented with 0.5% FBS for 24 hours prior to the initiation of downstream molecular experiments. Cells were then randomized into two experimental groups (Scramble siRNA and *Adam17* siRNA) and treated in starvation medium for 18 hours under the following conditions: Normoxia (21% O_2) and Hypoxia (1% O_2). After treatment, both cell lysates and conditioned media were collected for subsequent molecular analyses.

In parallel experiments, HAoECs subjected to siRNA transfection and serum starvation were treated with 1 ng/mL TGF-β1 (R&D Systems) in 0.5% FBS medium for 24 hours under either normoxic (21% O_2) conditions, serving as the control, or to hypoxic conditions (1% O_2) to mimic a pro-fibrotic environment and promote endothelial-to-mesenchymal transition (EndMT) (9, 10). To evaluate the effect of necroptosis inhibition *in vitro*, necrostatin-1 (25 μM, MCE HY-15760) was added to the culture medium during treatment under both normoxic and hypoxic conditions to inhibit necroptosis in endothelial cells while vehicle-treated cells (0.025% DMSO in PBS) used as controls (11, 12). To further assess the role of necroptosis in EndMT, necrostatin-1 along with vehicle were similarly added under both control (normoxia+TGF-β1) and pro-fibrotic (hypoxia+TGF-β1) conditions as described above.

Co-culture of HAoEC with Myofibroblasts

Primary cardiac fibroblasts (cFBs) were isolated from adult wild-type (WT) mice using a Langendorff perfusion system, as previously described (13). Isolated cFBs were cultured in DMEM/F12 medium (Gibco) supplemented with 5% FBS, 100 U/mL penicillin, and 100 μg/mL streptomycin in a humidified incubator at 37 °C with 5% CO_2. Passage 1 fibroblasts were used to generate myofibroblasts (MyoFBs) via treating cFB with 1 ng/mL TGF-β for 24 hours. For co-culture experiments, HAoECs previously transfected with siRNA (as described above) were seeded on top of the MyoFB monolayer at a 1:1 seeding density. The culture medium was replaced with a 1:1 mixture of DMEM/F12 (5% FBS, penicillin/streptomycin) and vascular cell basal medium (VCBM). Co-cultures were maintained for 16 hours under the following conditions: (1) Normoxia + saline; (2) Normoxia + TGFβ (1ng/ml TGFβ); (3) Hypoxia + saline and (4) Hypoxia

+ TGFβ (1ng/ml TGFβ). Following treatment, cells were stained to evaluate endothelial cell growth and branching.

Endothelial Cell derived Conditioned Medium treatment of Cardiac Fibroblasts

To investigate the effect of endothelial ADAM17 deficiency and the paracrine influence of endothelial cells on cardiac fibroblast activation, conditioned medium was collected from endothelial cells (WT or ADAM17-depleted) cultured under normoxic or hypoxic conditions. Cardiac fibroblasts (cFBs) were then treated with the collected conditioned medium and maintained under normoxic conditions for 24 h prior for downstream analyses.

Histological and Immunofluorescent Staining

At designated time points, hearts were excised and arrested in diastole using 1 M potassium chloride (KCl), fixed in 10% neutral-buffered formalin, embedded in paraffin, and sectioned at a thickness of 5 µm. Masson's Trichrome staining was performed to assess overall cardiac morphology and visualize fibrotic regions within the infarcted area, as previously described (6).

For immunofluorescence, hearts were embedded in OCT compound, cryosectioned, and stained using standard protocols (14). Briefly, cryosections were fixed in 4% paraformaldehyde (PFA) at room temperature, permeabilized with 0.1% Triton X-100 for 5 minutes, and blocked with 4% bovine serum albumin (BSA) for 1 hour. Sections were then incubated with primary antibodies diluted in 2% BSA overnight at 4 °C, followed by incubation with fluorescent-conjugated secondary antibodies for 1 hour at room temperature. Nuclei were counterstained using ProLong™ Gold Antifade Reagent with DAPI (Invitrogen, P36931).

Fluorescence images were acquired using an Olympus IX81 fluorescence microscope and analyzed using CellSens software. Pearson's correlation coefficient (R value) was calculated for colocalization analysis using ImageJ software. Primary antibodies used included: CD68 (Bio-Rad, MCA1957), Ly6G (BioLegend, 127602), CD31 (BD Pharmingen, 550274), CD8a (Invitrogen, 14-0808-82), CD4a (BD, 550280), Myeloperoxidase/MPO (R&D Systems, AF3667), Citrullinated Histone H3 (Abcam, ab5103), VE-Cadherin (Cell Signaling Technology, 2500S), Vimentin (Abcam, ab20346), αSMA (Abcam, ab7817)), anti-pMLKL (Cell signaling, 37333) and ADAM17 (Millipore, AB19027). Secondary antibodies included: Cy3 goat anti-rat (Invitrogen, A10522), Alexa Fluor 488 anti-rabbit (Invitrogen, A21206), Alexa Fluor 594 anti-rabbit (Invitrogen, A21207), Alexa Fluor 594 anti-mouse (Invitrogen, A21203), and Alexa Fluor 594 anti-goat (Life Technologies, A11058). Quantification of positive staining was performed by calculating the percentage of fluorescent-positive area relative to DAPI-stained nuclei within each field in CellSens Dimention V3.2. Seven to eleven random fields per section were analyzed, averaged, and presented as a single data point per sample in the graphs. Each data point in the quantification plots represents one biologically independent sample.

Protein Extraction

Flash-frozen left ventricular tissue (Sham, infarct, peri-infarct and non-infarct) and cultured HAoECs were homogenized by freeze-crushing in CellLytic™ M tissue lysis buffer (Sigma, Cat. No. C2978) supplemented with EDTA-free protease inhibitor cocktails. For protein isolation from culture media, trichloroacetic acid (TCA) precipitation was performed using TCA protein precipitation protocol from Dr. Luis Sanchez (15). Briefly, one volume of 100% (w/v) TCA was added to four volumes of the media sample. The mixture was incubated at 4 °C for 10 minutes, then centrifuged at 14,000 rpm for 5 minutes. The supernatant was carefully removed, leaving the protein pellet intact. The pellet was washed twice with 200 µL of cold acetone, each followed by centrifugation at 14,000 rpm for 5 minutes. After the final wash, pellets were dried at 95 °C for 5-10 minutes to remove residual acetone and then resuspended in PBS.

Immunoblotting

Immunoblotting was performed using PVDF membrane (Millipore, 0.45µm) as before (5, 8). he following primary antibodies were used: anti-pro-LOX (Abcam, ab174316), anti-LOX (Abcam, ab31238), anti-pSMAD2/3 (Cell Signalling, 8828S), anti-SMAD2/3 (Cell Signalling, 3102S), anti-pSMAD2/3 (Abcam, ab272332), anti-SMAD2/3 (Sigma, 07-408), anti-Fibronectin (Abcam, ab2413), anti-pVEGFR2 (Cell Signalling, 2478S), anti-VEGFR2(Cell Signalling, 2479S), anti-NOTCH1 (Santa Cruz, 373891), anti-VCAM1 (Santa Cruz, 13160), anti-ICAM1 (Santa Cruz, 8439), anti-JAMA (Santa Cruz, 53623), anti-GZMB(Cell Signalling, 46890), anti-CXCR3 (Santa Cruz, 137140), anti-Pro-Caspase 8 (Cell signaling, 4790), anti-Cleaved Caspase 8 (Cell signaling, 95702S), anti-Pro-Caspase 3 (Cell signaling, 9665S), anti-Cleaved Caspase 3 (Novas, 31A1067), anti-pMLKL (Cell signaling, 37333), anti-MLKL (Cell signaling, 37705), anti-pMLKL (Abcam, ab184717), anti-MLKL (Abcam, ab187091), anti-RIPK3 (Cell signaling, 95702S), anti-RIPK1 (Cell signaling, 3493s), anti-TNFR1 (Proteintech, 60192-1), anti-TNFR2 (Santa Cruz, 8041), anti-TNFR2 (Cell signaling 3727S),anti-TNFα (Invitrogen, 701135), anti-pAKT1/2/3 (Santa Cruz, 377556) , anti-AKT1/2 (Santa Cruz, 55523) , anti-SM22α (Abcam, ab155272), anti-αSMA (Abcam, ab5694). HRP-conjugated secondary antibodies were used: anti-mouse IgG (Cell Signaling, 7076S), anti-rat IgG (Cell Signaling, 7077S) and anti-rabbit IgG (Cell Signaling, 7074S). For loading control, membranes were stained using the Pierce™ Reversible Protein Stain Kit for PVDF membranes (Thermo, 24585) to assess total protein. Where needed, membranes were stripped using Restore™ PLUS Western Blot Stripping Buffer (Thermo Scientific, 46430) and re-probed. Protein bands were visualized using Clarity Max Western ECL substrate (Bio-Rad, 1705062) and imaged with the ImageQuant LAS 4000 system (GE Healthcare). Band intensities were quantified using ImageQuant TL software and normalized to total protein loading.

Hydroxyproline assay

Hydroxyproline assay was performed to quantify soluble and insoluble collagen content in cardiac tissue, as previously described (5, 16). Flash-frozen infarct, peri-infarct, and non-infarct regions of the left ventricle were lyophilized, and the dry weight of each sample was recorded. Lyophilized

tissue was then pulverized and suspended in 1 N NaCl containing protease and phosphatase inhibitors, and incubated at 4 °C with gentle agitation overnight. Following incubation, samples were centrifuged to separate the soluble collagen fraction (supernatant) and the insoluble fraction (pellet). Both fractions were subjected to acid hydrolysis in sealed glass ampules with 6 N HCl at 120 °C for 18 hours. After hydrolysis, samples were neutralized using 6 N NaOH. For colorimetric detection, 250 µL of 1.4% chloramine-T solution was added to 500 µL of each neutralized sample to oxidize hydroxyproline (20 minutes at room temperature), followed by the addition of Ehrlich's reagent and incubation at 65 °C to develop the chromophore. The reaction was stopped by adding cold water, and absorbance was measured at 558 nm using a microplate reader (SpectraMax iD5). Soluble and insoluble collagen contents were normalized to the corresponding dry tissue weight and expressed as micrograms of collagen per milligram of dry left ventricular tissue (µg/mg). Total collagen content was calculated as the sum of soluble and insoluble fractions.

Micro-Computed Tomography (micro-CT) Imaging of Coronary Vasculature

To visualize coronary vessels, hearts were excised and cannulated via the aorta for retrograde perfusion. Initially, hearts were flushed with heparinized saline to remove blood, followed by perfusion with the radiopaque contrast agent Microfil MV-122 Yellow (Flow Tech Inc., MA, USA) at a flow rate of 0.5 mL/min. Perfusion continued until the contrast was observed in the coronary sinus, at which point the aorta was clamped to prevent outflow. Hearts were then left at room temperature for 20 minutes to allow partial polymerization of the Microfil agent and subsequently fixed in 10% formalin at 4 °C overnight for complete polymerization.

The next day, hearts were rinsed with saline and scanned using a high-resolution micro-CT system (MILabs, Netherlands). Each heart was placed in a tube and imaged in three segments using MILabs Acquisition software. Image reconstruction was carried out with MILabs Reconstruction software following a modified protocol [21]. Reconstructed datasets were processed and analyzed using 3D Slicer software. To minimize background noise and exclude extravasated Microfil, a threshold of 2000 was applied. Coronary vessel volume was quantified using the "Closed Surface Statistics" function within 3D Slicer.

Propidium Iodide (PI)-Based Cell Death Assessment

Mice were anesthetized with isoflurane and administered 100 µL of propidium iodide (PI) solution (1 mg/mouse; Invitrogen, P3566) via retro-orbital injection 30 minutes prior to euthanasia. Immediately after sacrifice, hearts were excised, arrested in diastole using 1 M KCl, fixed in 10% neutral-buffered formalin, embedded in OCT compound, and cryosectioned at 5 µm thickness. Following sectioning, nuclei were counterstained with ProLong™ Gold Antifade Reagent containing DAPI (Invitrogen). Fluorescent images were captured using an Olympus IX81 fluorescence microscope and analyzed as previously described.

For *in vitro* cell death assessment, human aortic endothelial cells (HAoECs) were treated with normoxia (21% O_2) or hypoxia (1% O_2) as described above. After treatment, cells were incubated with propidium iodide (1 µg/mL) for 15 minutes at 37 °C, washed with PBS, and immediately

imaged using an Olympus IX81 fluorescence microscope. PI-positive nuclei were quantified as an index of dead cells.

Single-Nucleus RNA Sequencing (snRNA-seq) Analysis

For single-nucleus RNA sequencing (snRNA-seq), three types of left ventricular (LV) tissue samples were collected: (1) sham-operated LV free wall, (2) peri-infarct region of the LV free wall at post-MI day 3, and (3) infarct region of the LV free wall at post-MI day 3. Samples were obtained from both *Adam17*$^{f/f}$ and *Adam17*$^{EC\text{-}KD}$ mice. Tissues from three biological replicates (n=3) were pooled for each condition, and nuclei were isolated and processed to generate a single sequencing library per group. Publicly available snRNA-seq data from human myocardial infarction tissue were obtained from previously published datasets (17). The processed dataset is accessible through the Zenodo data repository (https://zenodo.org/records/6578047).

10x Genomics Single-Nucleus Library Preparation and Sequencing

Frozen cardiac tissue samples were minced into small fragments in a petri dish containing 1 mL of ice-cold lysis buffer composed of 0.32 M sucrose, 5 mM $CaCl_2$, 5 mM $Mg(Ac)_2$, 20 mM Tris-HCl (pH 7.5), 0.10% Triton X-100, 2 mM EDTA (pH 8.0),1mM DTT, sterile water, and 0.16 U/mL RNase inhibitor (Millipore Sigma, Cat. No. 3335402001). The tissue lysate was transferred to a 2 mL tissue grinder (Millipore Sigma, Cat. No. D8938), and an additional 1 mL of cold lysis buffer was added. Tissue was homogenized using five strokes with the loose pestle (Pestle A), followed by five strokes with the tight pestle (Pestle B). The homogenate was incubated on ice for 10 minutes to allow lysis. The resulting lysate was filtered through a 30 μm MACS® SmartStrainer (Miltenyi Biotec, Cat. No. 130-098-458), and nuclei were pelleted by centrifugation at 800 × g for 10 minutes at 4 °C. The supernatant was discarded, and the nuclei pellet was resuspended in 2 mL of wash buffer consisting of 1× PBS, 1% BSA, and 0.24 U/μL RNase inhibitor. The wash step was repeated once more by centrifugation at 800 × g for 10 minutes at 4 °C. After the second wash, the nuclei suspension was again filtered through a 30 μm SmartStrainer. The final nuclei pellet was resuspended in wash buffer. Nuclei integrity and concentration were assessed by DAPI staining (Thermo Fisher, Cat. No. D1306) and visualized under a fluorescent microscope using a hemocytometer (Thermo Fisher, Cat. No. 22-600-100). Nuclei were then diluted to the appropriate volume for target capture of 10,000 nuclei and loaded onto a 10x Genomics Single Cell 3' G Chip for droplet generation. Following droplet formation, emulsions were transferred to a pre-chilled strip tube (USA Scientific, Cat. No. US14024700) and incubated in a Bio-Rad thermal cycler. cDNA was recovered using the 10x Recovery Agent and purified using Silane DynaBeads (Thermo Fisher), following the manufacturer's protocol (10x Genomics Single Cell 3' Reagent Kits v3.1). Purified cDNA was amplified for 13 PCR cycles and cleaned using SPRIselect beads (Beckman Coulter). cDNA concentration and size distribution were assessed using an Agilent Bioanalyzer. Final libraries were prepared according to the 10x Genomics Single Cell 3' v3.1 user guide, with PCR cycle adjustments based on measured cDNA concentrations. Library molarity was calculated based on fragment size (Bioanalyzer, Agilent Technologies) and qPCR quantification (Roche, Cat.

No. 07960140001). Gene expression libraries were sequenced on an Illumina NovaSeq X platform at the Princess Margaret Genomics Centre, targeting approximately 400 million reads per sample.

Preprocessing and Quality Control

Raw sequencing data were first processed using Cell Ranger (v9.0.0) (18) for alignment and quantification. Each sample was aligned individually to the mouse reference genome (refdata-gex-GRCm39-2024-A) obtained from the 10x Genomics website, then aggregated into a combined dataset. Barcode rank plots were examined to assess library quality. Ambient RNA contamination was removed using CellBender (v0.3.2) (19), with a contamination fraction of 10%.

All downstream analyses were conducted in R (v4.3.2) using the Seurat package (v5.0.1) (20). Adjusted count matrices from CellBender were used as input. Cells with fewer than 500 detected genes were filtered out as low-quality, and cells with >20,000 genes or >5% mitochondrial gene content were excluded as potential doublets/multiplets or compromised cells. Each sample was initially preprocessed independently and later merged using the merge() function in Seurat for integrated analysis.

Normalization, Dimensionality Reduction, and Clustering

Data normalization and variable gene identification were performed using the NormalizeData function in Seurat, which applies regularized negative binomial regression to minimize technical variation. The variance-stabilizing transformation (VST) method was used to select the top 3,000 highly variable features. Technical confounders, including total RNA count (nCount_RNA) and mitochondrial content, were regressed out. Principal Component Analysis (PCA) was used for linear dimensionality reduction. The top 20 statistically significant PCs were used for downstream analyses. Harmony was applied for batch correction across samples. Graph-based clustering was performed using FindNeighbors and FindClusters, and Uniform Manifold Approximation and Projection (UMAP) was used for visualization. UMAP plots were used to evaluate clustering by sample identity and cell type.

Cell Type Annotation

Cell types were annotated using the FindAllMarkers function (Wilcoxon Rank Sum test, min.pct = 0.25, logFC.threshold = 0.25, adjusted $P < 0.05$). Heatmaps were generated using the top 10 differentially expressed genes (DEGs) per cluster. Canonical marker genes were used for cell type identification using DotPlot and FeaturePlot: Cardiomyocyte (*Myh7, Ryr2*), Fibroblast (*Dcn, Pdgfra*), Endothelial cells (*Pecam1, Cdh5*), Macrophages (*C1qa, C1qc*), Neutrophils (*S100a9, Siglecf*), B cells (*Cd79a, Cd79b*), T cells (*Cd3d, Cd3e*), Smooth muscle cells (*Tagln, Myh11*) and Pericytes (*Abcc9, Rgs5*) (17, 21, 22).

Differential Gene Expression and Pathway Enrichment Analysis

To investigate the effect of endothelial ADAM17 loss, cells were subset by cell type using the subset() function in Seurat. The FindMarkers() function (Wilcoxon Rank Sum test, min.pct = 0.25,

logFC.threshold = 0.25, adjusted $p < 0.05$) was used to identify DEGs between *Adam17*$^{EC-KD}$ and *Adam17*$^{f/f}$ samples within each condition (sham, infarct, and peri-infarct). Gene Ontology (GO) enrichment analysis was performed using the clusterProfiler package to identify overrepresented biological processes (BP), cellular components (CC), and molecular functions (MF) from the DEG lists (http://www.geneontology.org) (23). Enrichment was reported as -log10 adjusted P values along with fold enrichment. Z-score normalization was used for expression comparison and visualization.

Endothelial Cell Subclustering

To investigate heterogeneity within endothelial populations, endothelial cells were isolated and re-clustered. Preprocessing followed the same pipeline as described above, using the top 3,000 variable genes, 20 PCs, and Harmony batch correction. Clustering and UMAP visualization were performed using FindNeighbors, FindClusters, and RunUMAP. DEGs across endothelial subclusters were identified using FindAllMarkers and visualized via heatmaps. Functional enrichment for each endothelial subcluster was performed as described previously.

Receptor-Ligand Interaction Analysis (NicheNet)

Receptor-ligand communication was assessed using the Cellchat R package (v2.1.2) (24). Endothelial subclusters were designated as sender cells, while macrophages, fibroblasts, and cardiomyocytes were set as receiver populations. Ligand activity analysis was conducted to identify predicted target genes and receptors for the most active ligands. Interactions were filtered to include only literature-supported and database-validated ligand-receptor pairs.

***In vivo* Neutrophil,Cytotoxic T cell depletion and Necroptosis inhibition**

Neutrophils were depleted *in vivo* using an anti-Ly6G monoclonal antibody (Bio X Cell, BE00075). Mice received intraperitoneal injections of 250 μg anti-Ly6G antibody diluted in sterile PBS on pre-MI day 1, and post-MI days 1 and 3. Depletion efficacy was confirmed by flow cytometric analysis of peripheral blood and spleen sample using $CD64^-$, $Ly6G^+$ gating strategy **(Figure S2D)**. Control animals were injected with an equivalent dose of isotype control (Bio X Cell, BE00090) antibody following the same schedule. Cytotoxic T cells were depleted using anti-CD8α monoclonal antibody (Bio X Cell, BE0117). Mice received 200 μg of anti-CD8 antibody intraperitoneally on pre-MI day 1 and post-MI day 2. To confirm depletion efficacy, blood was collected via cardiac puncture following CO_2 euthanasia and transferred into heparinized tubes. After red blood cell lysis, cell suspensions were incubated for 30 minutes at 4 °C with fluorophore-conjugated antibodies against CD45 (clone 30-F11), Ly6G (clone 1A8), CD3 (17A2), and CD8 (53-6.7); all from BioLegend. Flow cytometric data were acquired on an LSRFortessa X-20 cytometer (BD Biosciences) and analyzed using FlowJo v10 software (Tree Star) **(Figure S2E)**.
To inhibit necroptosis *in vivo*, both *Adam17*$^{f/f}$ and *Adam17*$^{EC-KD}$ mice were administered daily intraperitoneal injections of Necrostatin-1 (Nec-1; MCE HY-15760) at 0.6 mg/kg body weight, beginning on the day of MI induction and maintained through post-MI day 7.

Statistical Analysis

All statistical analyses were performed using IBM SPSS Statistics v21 and GraphPad Prism. Data were assessed for normality using the Shapiro-Wilk test. For comparisons between two groups, Student's two-tailed t-test was used. For comparisons among multiple groups, two-way ANOVA (factors: genotype and treatment) was applied, followed by Bonferroni or Tukey post hoc tests, as appropriate. Kaplan-Meier survival curves were analyzed using the log-rank (Mantel-Cox) test. Sample sizes (n) represent the number of mice used *in vivo* studies or the number of independent biological replicates in vitro experiments. Data are presented as mean ± standard error of the mean (SEM). A p-value < 0.05 was considered statistically significant.

REFERENCES

1. Horiuchi K, Kimura T, Miyamoto T, Miyamoto K, Akiyama H, Takaishi H, et al. Conditional inactivation of TACE by a Sox9 promoter leads to osteoporosis and increased granulopoiesis via dysregulation of IL-17 and G-CSF. J Immunol. 2009;182(4):2093-101.
2. Horiuchi K, Kimura T, Miyamoto T, Takaishi H, Okada Y, Toyama Y, et al. Cutting edge: TNF-alpha-converting enzyme (TACE/ADAM17) inactivation in mouse myeloid cells prevents lethality from endotoxin shock. J Immunol. 2007;179(5):2686-9.
3. Wang Y, Nakayama M, Pitulescu ME, Schmidt TS, Bochenek ML, Sakakibara A, et al. Ephrin-B2 controls VEGF-induced angiogenesis and lymphangiogenesis. Nature. 2010;465(7297):483-6.
4. Chen X, Zhabyeyev P, Azad AK, Wang W, Minerath RA, DesAulniers J, et al. Endothelial and cardiomyocyte PI3Kbeta divergently regulate cardiac remodelling in response to ischaemic injury. Cardiovasc Res. 2019;115(8):1343-56.
5. Chute M, Aujla PK, Li YX, Jana S, Zhabyeyev P, Rasmuson J, et al. ADAM15 is required for optimal collagen cross-linking and scar formation following myocardial infarction. Matrix Biology. 2022;105:127-43.
6. Fan D, Takawale A, Shen MC, Wang W, Wang XH, Basu R, et al. Cardiomyocyte A Disintegrin And Metalloproteinase 17 (ADAM17) Is Essential in Post-Myocardial Infarction Repair by Regulating Angiogenesis. Circ-Heart Fail. 2015;8(5):970-9.
7. Dann MM, Clark SQ, Trzaskalski NA, Earl CC, Schepers LE, Pulente SM, et al. Quantification of murine myocardial infarct size using 2-D and 4-D high-frequency ultrasound. Am J Physiol-Heart C. 2022;322(3):H359-H72.
8. Aujla PK, Hu M, Hartley B, Kranrod JW, Viveiros A, Kilic T, et al. Loss of ADAM15 Exacerbates Transition to Decompensated Myocardial Hypertrophy and Dilation Through Activation of the Calcineurin Pathway. Hypertension. 2023;80(1):97-110.
9. Evrard SM, Lecce L, Michelis KC, Nomura-Kitabayashi A, Pandey G, Purushothaman KR, et al. Endothelial to mesenchymal transition is common in atherosclerotic lesions and is associated with plaque instability. Nat Commun. 2016;7:11853.
10. Zhou G, Dada LA, Wu M, Kelly A, Trejo H, Zhou Q, et al. Hypoxia-induced alveolar epithelial-mesenchymal transition requires mitochondrial ROS and hypoxia-inducible factor 1. Am J Physiol Lung Cell Mol Physiol. 2009;297(6):L1120-30.
11. An S, Qi Y, Zhang Z, Mo R, Hou L, Yao X, et al. Antagonism of receptor interacting protein 1 using necrostatin-1 in oxidized LDL- induced endothelial injury. Biomed Pharmacother. 2018;108:1809-15.
12. Liu L, Liang L, Yang C, Zhou Y, Chen Y. Extracellular vesicles of Fusobacterium nucleatum compromise intestinal barrier through targeting RIPK1-mediated cell death pathway. Gut Microbes. 2021;13(1):1-20.
13. Chute M, Aujla PK, Li Y, Jana S, Zhabyeyev P, Rasmuson J, et al. ADAM15 is required for optimal collagen cross-linking and scar formation following myocardial infarction. Matrix Biol. 2022;105:127-43.
14. Fan D, Takawale A, Shen M, Wang W, Wang X, Basu R, et al. Cardiomyocyte A Disintegrin And Metalloproteinase 17 (ADAM17) Is Essential in Post-Myocardial Infarction Repair by Regulating Angiogenesis. Circ Heart Fail. 2015;8(5):970-9.

15. Sanchez L. TCA protein precipitation protocol 2001 [Available from: https://www.its.caltech.edu/~bjorker/TCA_ppt_protocol.pdf.
16. Sakamuri SS, Takawale A, Basu R, Fedak PW, Freed D, Sergi C, et al. Differential impact of mechanical unloading on structural and nonstructural components of the extracellular matrix in advanced human heart failure. Transl Res. 2016;172:30-44.
17. Kuppe C, Ramirez Flores RO, Li Z, Hayat S, Levinson RT, Liao X, et al. Spatial multi-omic map of human myocardial infarction. Nature. 2022;608(7924):766-77.
18. Zheng GX, Terry JM, Belgrader P, Ryvkin P, Bent ZW, Wilson R, et al. Massively parallel digital transcriptional profiling of single cells. Nat Commun. 2017;8:14049.
19. Fleming SJ, Chaffin MD, Arduini A, Akkad AD, Banks E, Marioni JC, et al. Unsupervised removal of systematic background noise from droplet-based single-cell experiments using CellBender. Nat Methods. 2023;20(9):1323-35.
20. Hao Y, Stuart T, Kowalski MH, Choudhary S, Hoffman P, Hartman A, et al. Dictionary learning for integrative, multimodal and scalable single-cell analysis. Nat Biotechnol. 2024;42(2):293-304.
21. Koenig AL, Shchukina I, Amrute J, Andhey PS, Zaitsev K, Lai L, et al. Single-cell transcriptomics reveals cell-type-specific diversification in human heart failure. Nat Cardiovasc Res. 2022;1(3):263-80.
22. Marin-Sedeno E, de Morentin XM, Perez-Pomares JM, Gomez-Cabrero D, Ruiz-Villalba A. Understanding the Adult Mammalian Heart at Single-Cell RNA-Seq Resolution. Front Cell Dev Biol. 2021;9:645276.
23. Yu G, Wang LG, Han Y, He QY. clusterProfiler: an R package for comparing biological themes among gene clusters. OMICS. 2012;16(5):284-7.
24. Browaeys R, Saelens W, Saeys Y. NicheNet: modeling intercellular communication by linking ligands to target genes. Nat Methods. 2020;17(2):159-62.

SUPPLEMENTARY TABLES

Table S1: Echocardiography data of cardiac structure and function in **male** *Adam17*$^{f/f}$ and *Adam17*$^{EC\text{-}KD}$ mice before, and 3 and 7 days after induction of MI.

Parameters	Pre-MI		3 days Post-MI		7 days Post-MI	
	Adam17$^{f/f}$	*Adam17*$^{EC\text{-}KD}$	*Adam17*$^{f/f}$	*Adam17*$^{EC\text{-}KD}$	*Adam17*$^{f/f}$	*Adam17*$^{EC\text{-}KD}$
**ESV (µL)**	28.42 ± 0.81	30.23 ± 0.48	56.63 ± 2.87	69.2 ± 1.38*‡	87.78 ± 2.78	118.63 ± 1.92*‡
**EDV (µL)**	68.4 ± 1.72	70.96 ± 0.79	91.5 ± 3.4	95.34 ± 1.96*	129.9 ± 1.24	146.31 ± 2.03*‡
**EF (%)**	58.34 ± 1.16	57.4 ± 0.48	38.56 ± 1.54	27.36 ± 0.87*‡	32.45 ± 1.91	18.93 ± 0.37*‡
**LVAWd (mm)**	0.86 ± 0.02	0.84 ± 0.01	0.78 ± 0.03	0.79 ± 0.06*	0.69 ± 0.05	0.72 ± 0.05*
**LVAWs (mm)**	1.1 ± 0.02	1.17 ± 0.02	1.07 ± 0.05	0.99 ± 0.05*	0.94 ± 0.05	0.8 ± 0.06*
**LVIDd (mm)**	4.04 ± 0.11	4.01 ± 0.08	4.54 ± 0.09	4.73 ± 0.08*‡	5.29 ± 0.06	5.58 ± 0.12*
**LVIDs (mm)**	2.69 ± 0.08	2.82 ± 0.05	3.76 ± 0.15	4.07 ± 0.14*	4.55 ± 0.1	4.95 ± 0.13*‡
**LVPWd (mm)**	0.85 ± 0.03	0.85 ± 0.02	0.76 ± 0.04	0.69 ± 0.03*	0.62 ± 0.03	0.54 ± 0.03*
**LVPWs (mm)**	1.03 ± 0.03	1.11 ± 0.02	1.06 ± 0.04	0.84 ± 0.05*‡	0.88 ± 0.06	0.75 ± 0.04*‡
**FS (%)**	41.51 ± 0.9	46.29 ± 1.47	23.8 ± 4.31	15 ± 1.75*‡	10.91 ± 1.82	6.63 ± 0.47*‡
**LA (mm)**	1.75 ± 0.02	1.66 ± 0.02	2.11 ± 0.05	2.18 ± 0.05*	2.4 ± 0.08	2.53 ± 0.05*
**GLS (%)**	-21.28 ± 0.74	-20.58 ± 0.95	-4.74 ± 0.24	-3.89 ± 0.1*‡	-3.58 ± 0.19	-1.89 ± 0.13*‡
**GCS (%)**	-30.21 ± 0.59	-31.82 ± 0.48	-6.11 ± 0.11	-6.02 ± 0.13*	-2.62 ± 0.12	-2.55 ± 0.15*

EDV – End-diastolic volume; ESV – End-systolic volume; EF – Ejection fraction; IVSd/s - Interventricular septal thickness in diastole/systole; LVIDd/s - Left ventricular internal diameter in diastole/systolic; LVIDs - Left ventricular internal diameter in systole; LVPWd/s - Left ventricular posterior wall thickness in diastole/systole; FS - Fractional shortening; LA - Left atrial diameter; GLS - Global longitudinal strain and GCS - Global circumferential strain. Values are mean ±SEM. *$p < 0.05$ compared to corresponding sham group; ‡$p < 0.05$ compared to corresponding *Adam17*$^{f/f}$ MI group

Table S2: Echocardiography data of **male** wildtype (WT) and littermate *Cdh5*-Cre^{ERT2} mice before, and 3 and 7 days after induction of myocardial infarction (MI).

	**Pre-MI**		**3 days Post-MI**		**7 days Post-MI**	
**Parameters**	**WT**	***Cdh5*-Cre^{ERT2}**	**WT**	***Cdh5*-Cre^{ERT2}**	**WT**	***Cdh5*-Cre^{ERT2}**
**ESV (µL)**	28.15 ± 0.79	27.4 ± 0.47	54.46 ± 1.8	56.36 ± 2.56*	80.47 ± 1.53	86.17 ± 2.47*
**EDV (µL)**	67.32 ± 2.24	68.02 ± 1.09	89.38 ± 2.13	92.51 ± 3.36*	120.11 ± 2.4	125.8 ± 2.79*
**EF (%)**	57.98 ± 1.12	59.69 ± 0.76	39.16 ± 0.73	39.22 ± 0.64*	32.93 ± 1	31.5 ± 1.27*
**LVAWd (mm)**	0.85 ± 0.03	0.85 ± 0.02	0.77 ± 0.04	0.76 ± 0.05*	0.7 ± 0.04	0.67 ± 0.05*
**LVAWs (mm)**	1.12 ± 0.04	1.16 ± 0.04	1.01 ± 0.03	0.96 ± 0.04*	0.95 ± 0.04	0.8 ± 0.05*
**LVIDd (mm)**	3.87 ± 0.14	4.03 ± 0.13	4.52 ± 0.13	4.48 ± 0.19*	5.00 ± 0.11	5.33 ± 0.24*
**LVIDs (mm)**	2.67 ± 0.08	2.75 ± 0.08	3.69 ± 0.14	3.78 ± 0.19*	4.15 ± 0.1	4.61 ± 0.27*
**LVPWd (mm)**	0.87 ± 0.03	0.86 ± 0.02	0.72 ± 0.02	0.75 ± 0.03*	0.62 ± 0.05	0.64 ± 0.05*
**LVPWs (mm)**	1.06 ± 0.04	1.14 ± 0.03	0.93 ± 0.04	0.95 ± 0.05*	0.81 ± 0.04	0.84 ± 0.05*
**FS (%)**	45.01 ± 1.16	46.56 ± 2.17	24.76 ± 2.89	20.6 ± 2.53*	11.9 ± 1.98	13.1 ± 3.6*
**LA (mm)**	1.72 ± 0.02	1.65 ± 0.04	1.92 ± 0.06	2.04 ± 0.06*	2.2 ± 0.07	2.33 ± 0.12*
**GLS (%)**	-22.2 ± 0.68	-21.36 ± 0.61	-4.96 ± 0.27	-4.93 ± 0.21*	-4.07 ± 0.18	-3.89 ± 0.13*
**GCS (%)**	-31.02 ± 0.38	-29.5 ± 0.51	-5.95 ± 0.12	-6.19 ± 0.12*	-2.65 ± 0.13	-2.46 ± 0.22*

EDV – End-diastolic volume; ESV – End-systolic volume; EF – Ejection fraction; IVSd/s - Interventricular septal thickness in diastole/systole; LVIDd/s - Left ventricular internal diameter in diastole/systolic; LVIDs - Left ventricular internal diameter in systole; LVPWd/s - Left ventricular posterior wall thickness in diastole/systole; FS - Fractional shortening; LA - Left atrial diameter; GLS - Global longitudinal strain and GCS - Global circumferential strain. Values are mean ±SEM *$p < 0.05$ compared to corresponding sham group; ‡$p < 0.05$ compared to corresponding WT MI group

Table S3: Echocardiography data in **female** *Adam17*$^{f/f}$ and *Adam17*$^{EC-KD}$ mice at Pre-MI, Post-MI day 3 and Post-MI day7.

Parameters	Pre-MI		3 days Post-MI		7 days Post-MI	
	♀ *Ad17*^{WT}	♀ *Ad17*$^{EC-KD}$	♀ *Ad17*^{WT}	♀ *Ad17*$^{EC-KD}$	♀ *Ad17*^{WT}	♀ *Ad17*$^{EC-KD}$
**ESV (µL)**	25.21 ± 0.33	25.71 ± 0.33	59.69 ± 2.41	65.29 ± 2.9*	70.32 ± 1.97	80.8 ± 4.8*‡
**EDV (µL)**	52.68 ± 1.14	53.35 ± 0.72	87.79 ± 2.71	93.8 ± 3.41*	98.7 ± 2.84	108.55 ± 5.39*
**EF (%)**	52.06 ± 0.77	51.79 ± 0.3	32.14 ± 0.73	30.53 ± 0.76*	28.71 ± 0.81	25.91 ± 0.94*‡
**LVAWd (mm)**	0.89 ± 0.03	0.87 ± 0.03	0.73 ± 0.02	0.69 ± 0.02*	0.64 ± 0.01	0.63 ± 0.01*
**LVAWs (mm)**	1.12 ± 0.02	1.12 ± 0.02	0.85 ± 0.02	0.87 ± 0.02*	0.79 ± 0.01	0.79 ± 0.01*
**LVIDd (mm)**	3.69 ± 0.08	3.63 ± 0.07	4.58 ± 0.05	4.46 ± 0.06*	4.82 ± 0.07	4.89 ± 0.07*
**LVIDs (mm)**	2.31 ± 0.05	2.27 ± 0.03	3.34 ± 0.06	3.35 ± 0.09*	3.62 ± 0.08	3.64 ± 0.08*
**LVPWd (mm)**	0.83 ± 0.03	0.93 ± 0.02	0.76 ± 0.03	0.7 ± 0.03*	0.58 ± 0.02	0.57 ± 0.02*
**LVPWs (mm)**	1.13 ± 0.02	1.1 ± 0.02	0.85 ± 0.02	0.91 ± 0.02*	0.74 ± 0.01	0.73 ± 0.01*
**FS (%)**	50.37 ± 1.05	50.48 ± 1.03	25.91 ± 1.44	27.01 ± 1.36*	18.64 ± 0.75	16.14 ± 0.78*
**LA (mm)**	1.43 ± 0.03	1.45 ± 0.03	1.82 ± 0.03	1.83 ± 0.02*	2.15 ± 0.03	2.14 ± 0.04*
**GLS (%)**	-20.28 ± 0.42	-20.16 ± 0.37	-7.68 ± 0.45	-7.38 ± 0.59*	-5.88 ± 0.3	-5.38 ± 0.33*
**GCS (%)**	-28.21 ± 0.72	-27.31 ± 0.58	-15.36 ± 0.36	-14.46 ± 0.27*	-8.47 ± 0.32	-8.85 ± 0.26*

EDV – End-diastolic volume; ESV – End-systolic volume; EF – Ejection fraction; IVSd/s - Interventricular septal thickness in diastole/systole; LVIDd/s - Left ventricular internal diameter in diastole/systolic; LVIDs - Left ventricular internal diameter in systole; LVPWd/s - Left ventricular posterior wall thickness in diastole/systole; FS - Fractional shortening; LA - Left atrial diameter; GLS - Global longitudinal strain and GCS - Global circumferential strain. Values are mean ±SEM. *$p < 0.05$ compared to corresponding sham group; ‡$p < 0.05$ compared to corresponding *Adam17*$^{f/f}$ MI group

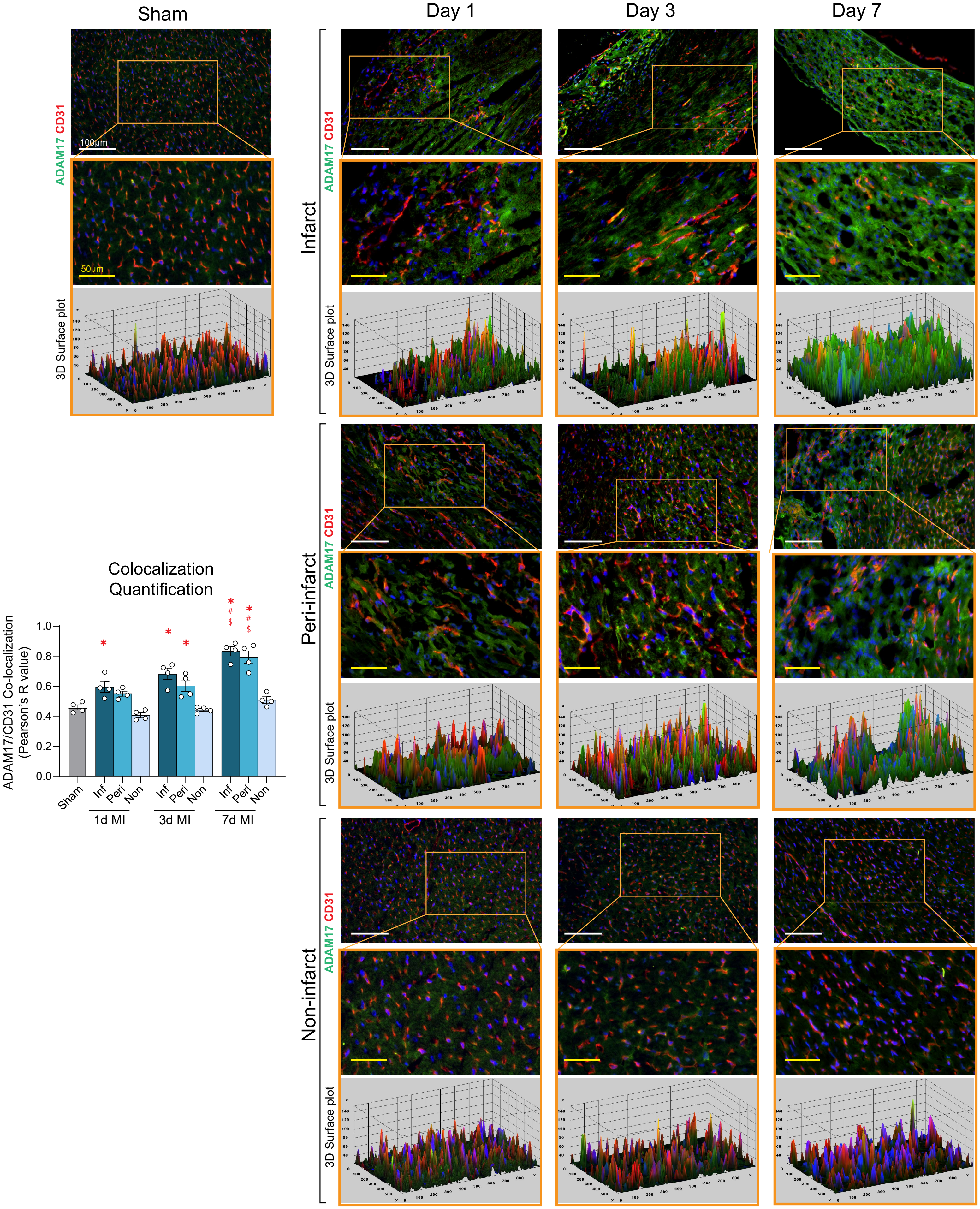

Figure S1: Temporal expression of ADAM17 in ECs following myocardial infarction in WT mice. Representative co-immunofluorescence staining for ADAM17 (green), endothelial cells (CD31, red) and nuclei (DAPI, blue), 3D surface plot and quantification for CD31-ADAM17 colocalization in sham (control), and in infarct, peri-infarct, and non-infarct regions at days 1, 3, and 7 post-MI (n=4 hearts/group; 8–10 sections per heart). Averaged Pearson's R values are presented as mean ± SEM. *$p < 0.05$ compared to sham; $^{\#}p < 0.05$ compared to corresponding 1d-MI; $^{\$}p < 0.05$ compared to corresponding 3d-MI.

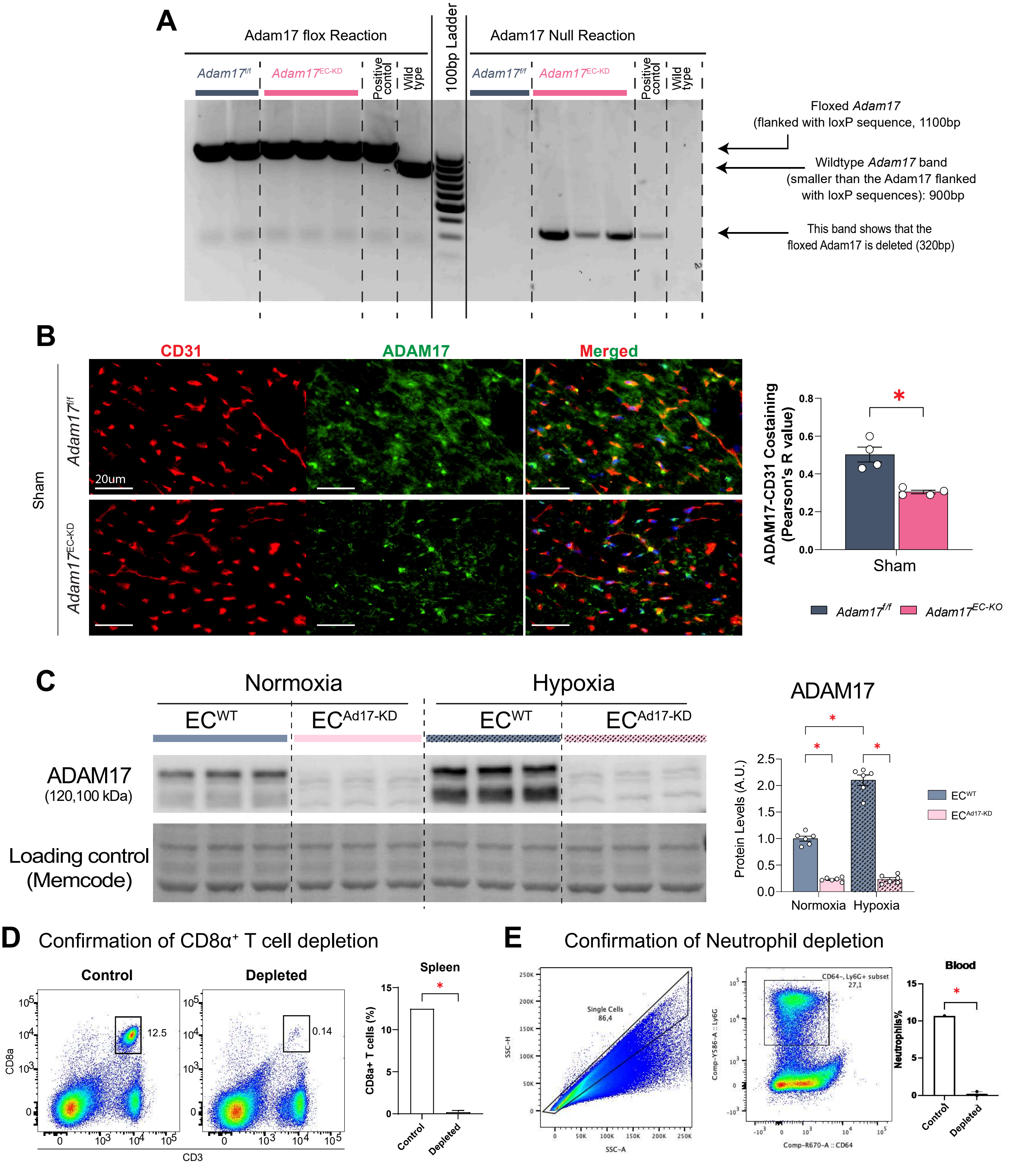

Figure S2: Validation of endothelial ADAM17 deletion, and depletion of inflammatory cell (CD8a+ cell and Neutrophils). **(A)** PCR validation of *Adam17* deletion in tamoxifen-treated *Adam17*$^{EC\text{-}KD}$ compared to *Adam17*$^{f/f}$ using primers that detect the floxed *Adam17* gene (Adam17 flox reaction) and the recombined null allele that happens in the presence of endothelial-specific Cre-recombinase enzyme (Adam17 Null reaction). **(B)** Representative immunofluorescence co-staining and quantification of endothelial cells(CD31, red) and ADAM17 (green) with nuclei counterstained by DAPI (blue), in the left ventricle of *Adam17*$^{f/f}$ and *Adam17*$^{EC\text{-}KD}$ mice (n=4 hearts/group, 6-8 images/heart). **(C)** Representative immunoblot and quantification of ADAM17 protein levels in human endothelial cells (ECs) treated with scrambled siRNA (EC^{WT}) and *Adam17* siRNA (EC$^{Ad17\text{-}KD}$) in normoxic (20% O_2) and hypoxic (1% O_2) conditions (n=3 independent cultures, 2 wells per group per experiment). **(D–E)** Flow cytometry on mouse blood confirming effective depletion of CD8+ T cells **(D)** or neutrophils **(E)** following *in vivo* antibody treatment in mouse**.** Averaged data are presented as Mean ± SEM. Statistical analysis was performed using unpaired t-test **(B)** or two-way ANOVA with Bonferroni post hoc test **(C)**. * $p < 0.05$ vs. *Adam17*$^{f/f}$.

A) Post-MI Survival and LV rupture in **Cdh5Cre^{ERT2} and WT** post-MI

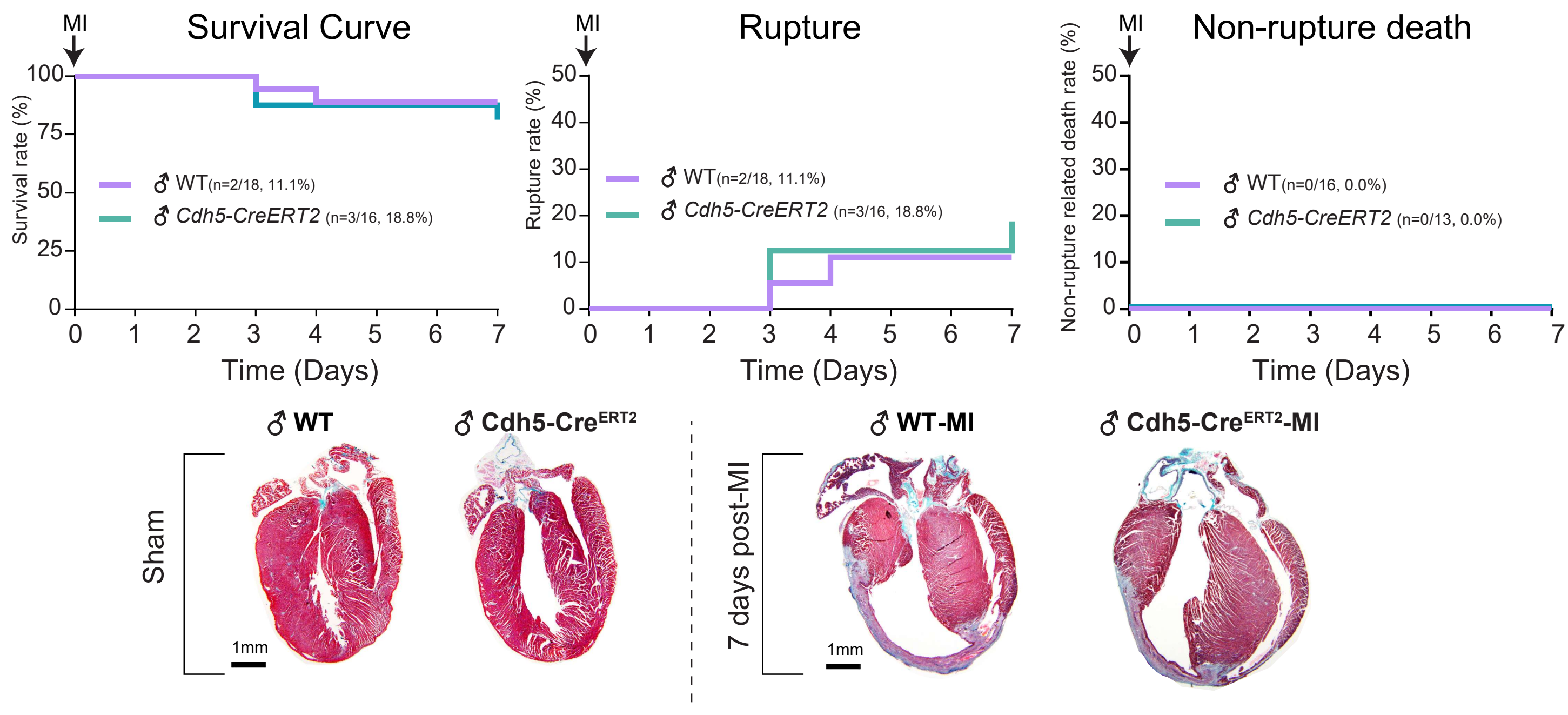

B) Post-MI Survival and rupture in **female** *Adam17*$^{f/f}$ and *Adam17*$^{EC\text{-}KD}$

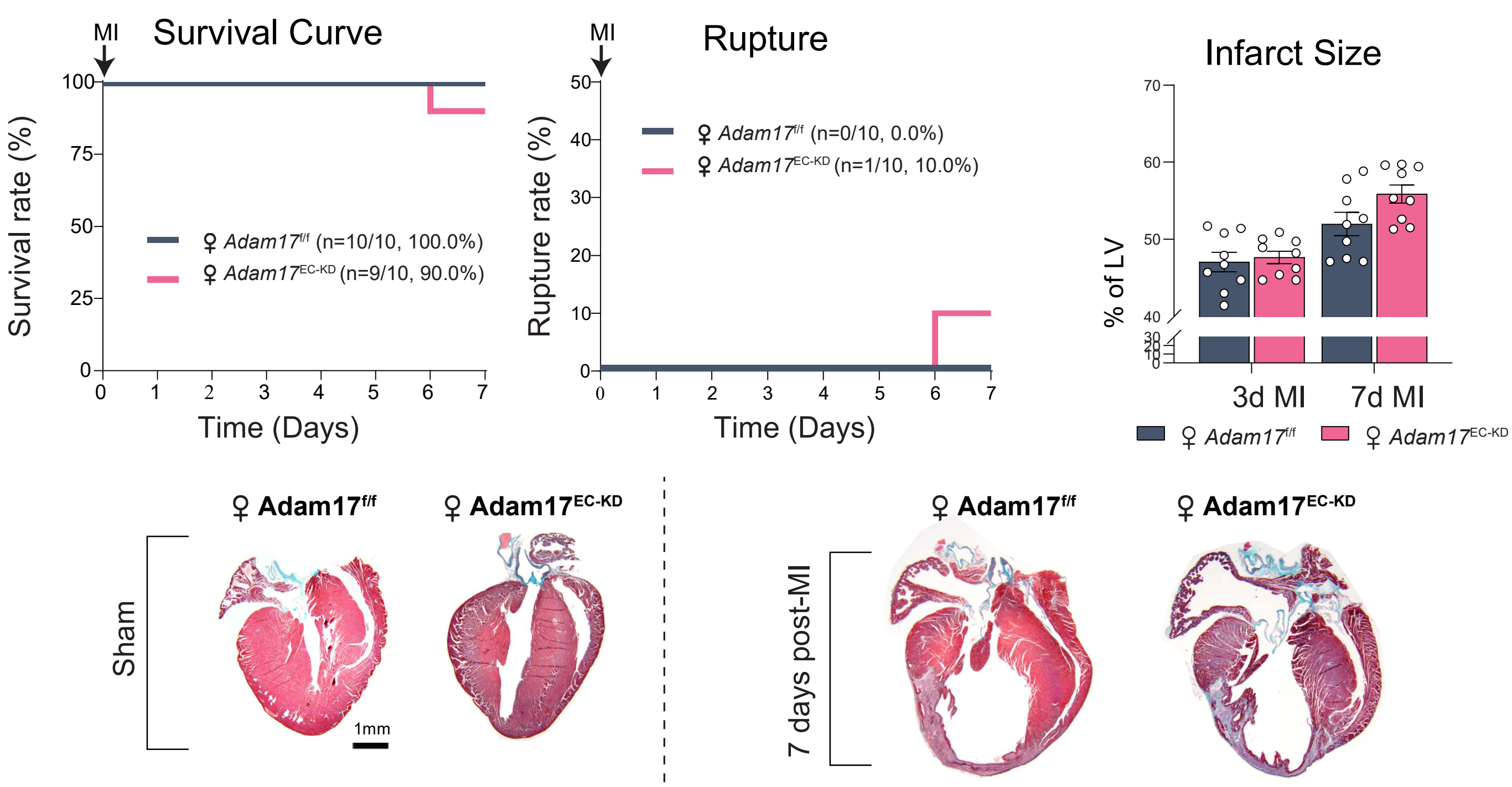

Figure S3: Post-MI survival in *Cdh5-Cre^{ERT2}* controls, and in female mice with endothelial ADAM17 knockdown.
(A) Kaplan–Meier curves for survival, left ventricular rupture, and non-rupture death incidences following myocardial infarctin (post-MI), in male mice expressing *Cdh5-Cre^{ERT2}* and littermate wildtype (WT) mice (all treated with tamoxifen prior to MI induction). Representative Masson's Trichrome-stained heart sections from sham and post-MI of each genotype. **(C)** Kaplan–Meier curves for survival and LV rupture in **female** *Adam17*$^{f/f}$ and *Adam17*$^{EC\text{-}KD}$ mice following MI. Echocardiographic assessment of infarct size at 3 and 7 days post-MI shows no difference between genotypes in females. Representative Masson's Trichrome-stained sections of sham and post-MI hearts from female *Adam17*$^{f/f}$ and *Adam17*$^{EC\text{-}KD}$ mice. Averaged data are presented as Mean ± SEM. Statistical analysis was performed using two-way ANOVA with Bonferroni post hoc test. **p*<0.05 vs *Adam17*$^{f/f}$.

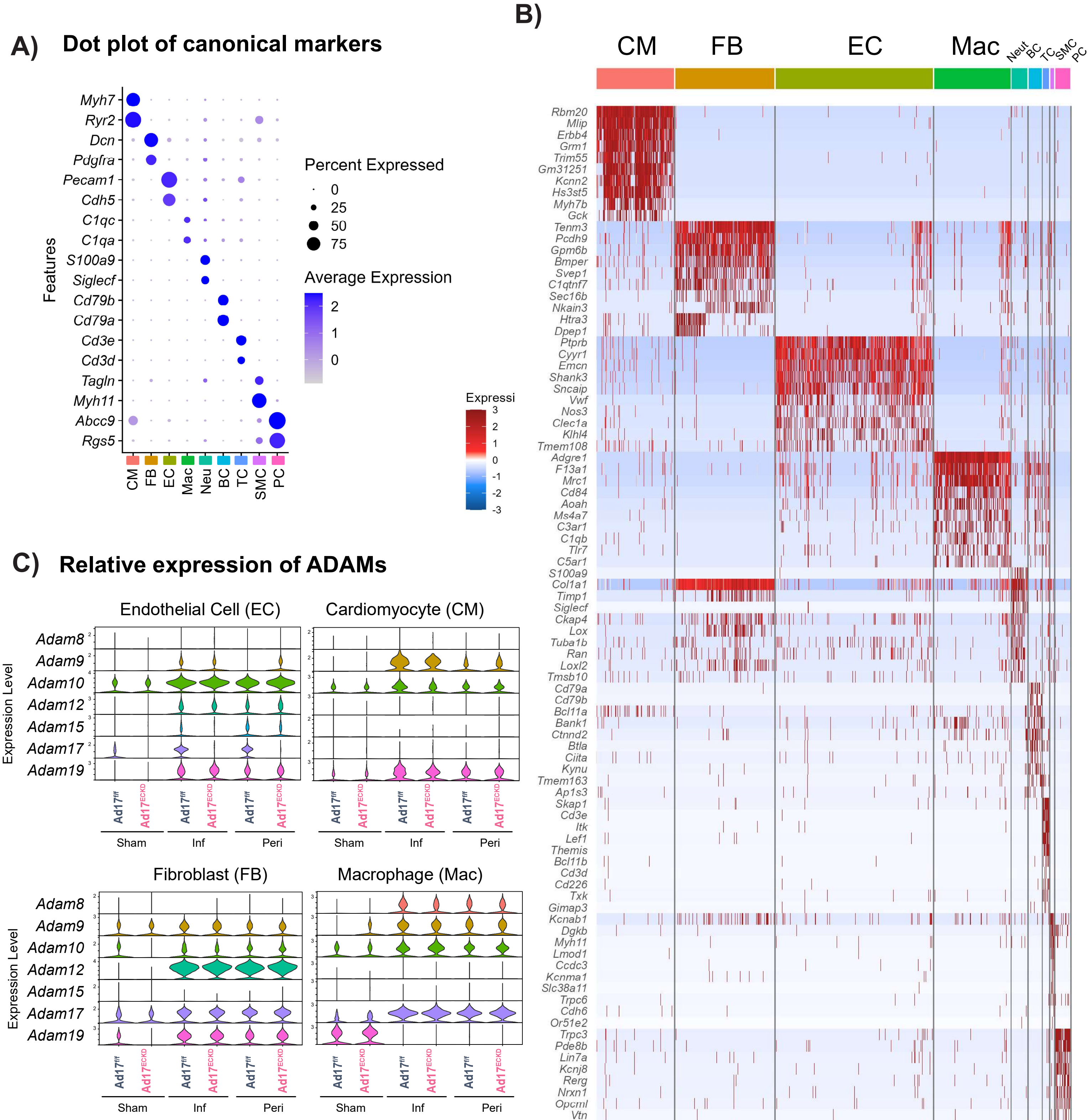

Figure S4: Single-nucleus RNA-seq identifies major cardiac cell populations in sham and post-MI hearts. (A) Dot plot of canonical marker genes used to assign major cell types. CM, cardiomyocyte; FB, fibroblast; EC, endothelial cell; Mac, macrophage; Neu, neutrophil; BC, B cell; TC, cytotoxic T cell; SMC, smooth muscle cell; PC, pericyte. **(B)** Heatmap showing the top-expressed genes across major cell populations in the heart. **(C)** Expression profiles of members of the *Adam* family that have been reported in the cardiovascular system, showing loss of *Adam17* in the endothelial cells (but not other cell types) in *Adam17*$^{EC-KD}$ compared to *Adam17*$^{f/f}$ hearts. Other Adams are altered comparably between genotypes in the infarct and peri-infarct reagions.

A) CD4 T cell staining (Post MI)

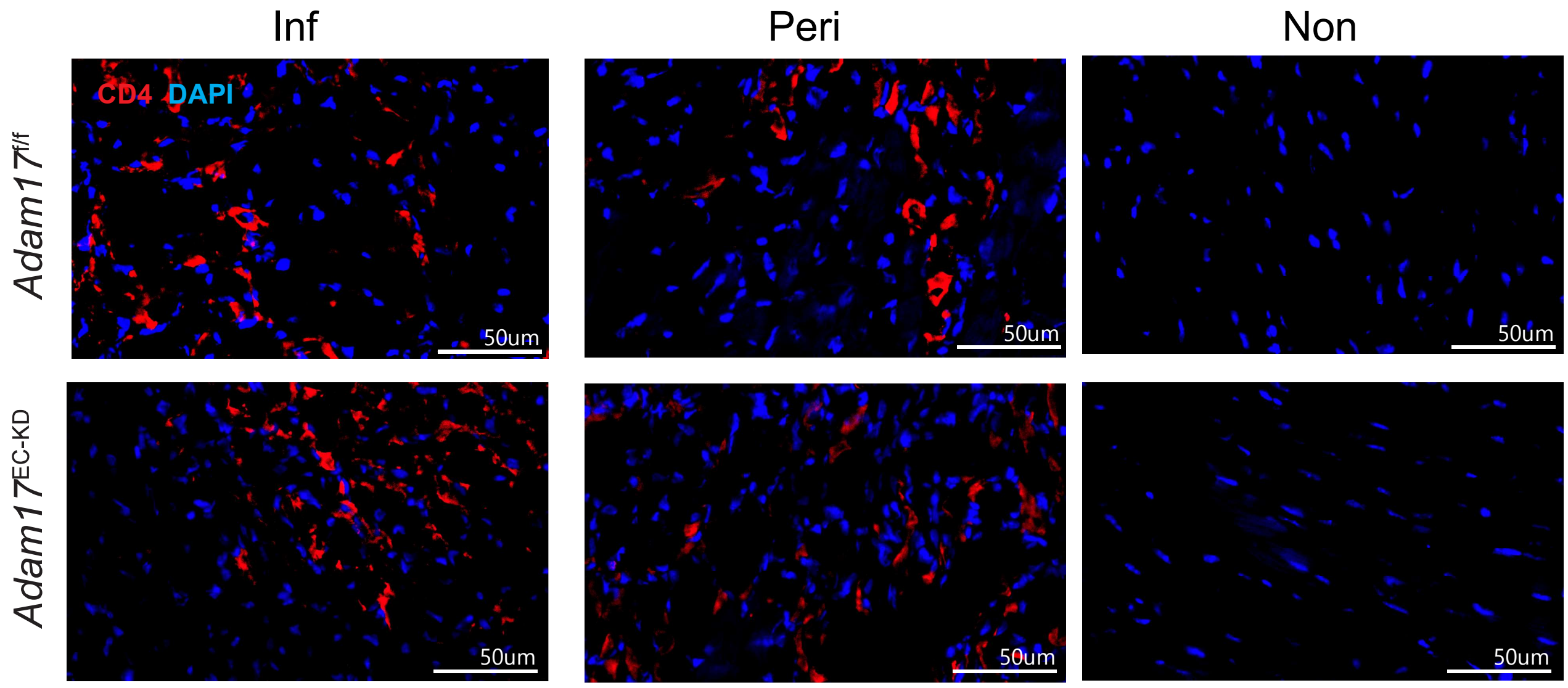

B)

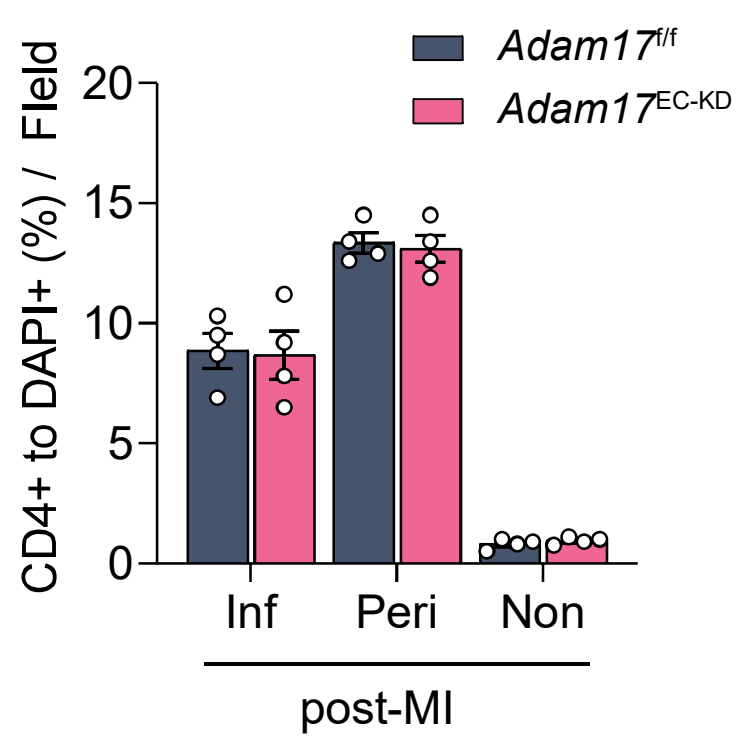

Figure S5: No difference in CD4+ T-Cell infiltration was detected between genotypes post-MI. Representative immunofluorescence images **(A)** and quantification **(B)** of $CD4^+$ T cells (CD4, red) and nuclei (DAPI, blue) in the infarct (Inf), peri-infarct (Peri), and non-infarct (Non) regions of *Adam17*$^{f/f}$ and *Adam17*$^{EC\text{-}KD}$ hearts at 3 days post-MI (n=4 hearts/group, 6-8 images/heart). Averaged data are presented as Mean ± SEM. Statistical analysis was performed using two-way ANOVA with Bonferroni post hoc test. * $p<0.05$ vs. *Adam17*$^{f/f}$.

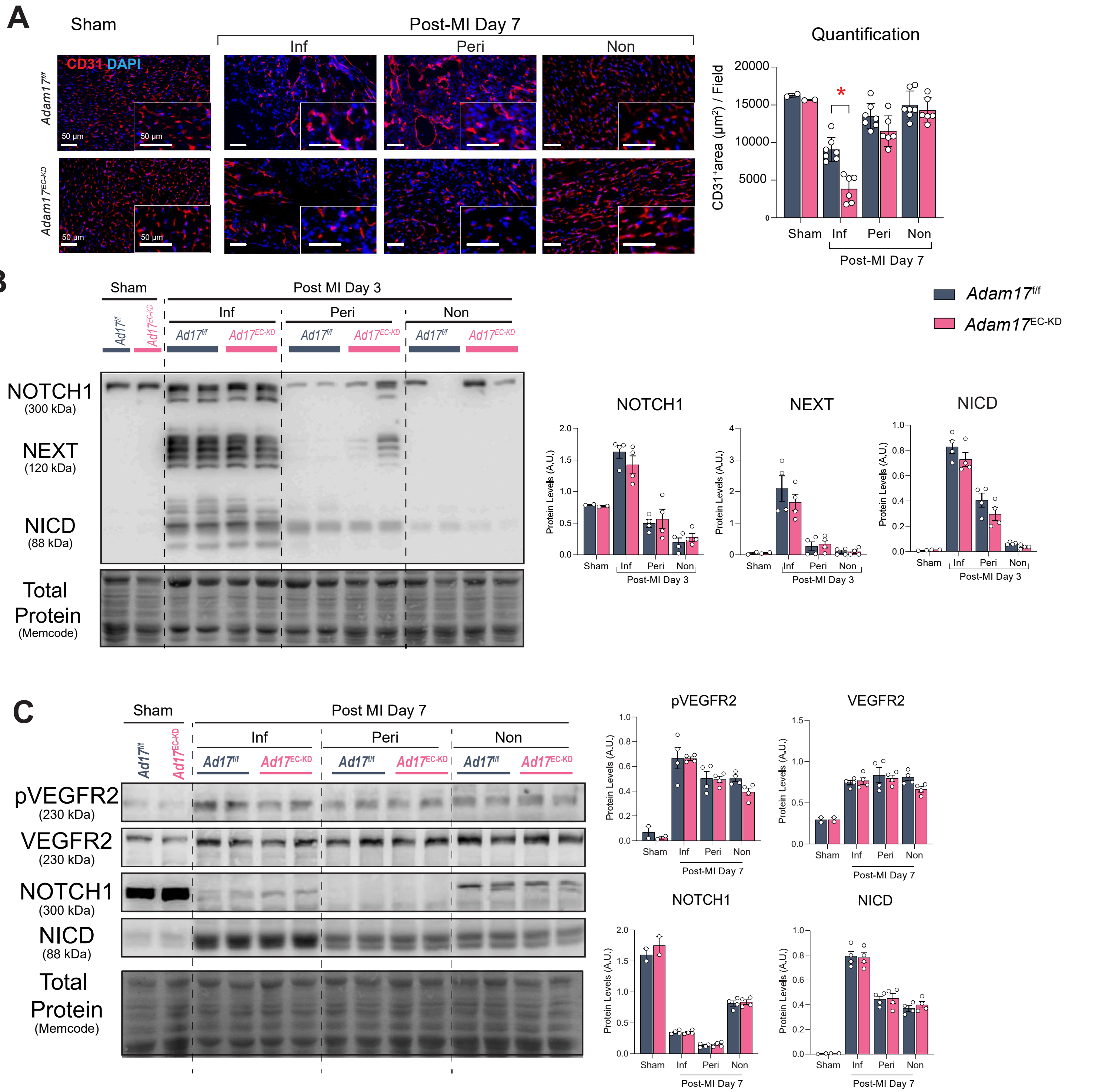

Figure S6: Endothelial *Adam17* deficiency resulted in sustained reduction in endothleila cell population, but did not alter VEGFR2 signalling post-MI compared to *Adam17$^{f/f}$* mice. **(A)** Representative CD31 immunofluorescence (red) and nuclear DAPI (blue) staining at 7 days post-sham or MI (Inf, Peri, and Non). Averaged CD31+ signal quantification are shown as bar graphs (n=4–6 hearts/group; 8–10 sections per heart). **(B)** Representative immunoblot and protein quantification of angiogenesis-associated proteins (NOTCH1, NEXT, NICD) in the LV at 3 days after sham or MI, in *Adam17$^{f/f}$* and *Adam17^{EC-KD}* hearts (n = 4–6 hearts/group). **(C)** Representative immunoblot and protein quantification for phosphorylated and total VEGFR2, NOTCH1 and NICD in sham and post-MI hearts (Inf, Peri, and Non) at day 7 post-MI (n=4–6 hearts/group). Inf=Infarct, Peri=peri-infarct, Non=non-infarct. A.U.=Arbitrary Units. Averaged data are presented as mean ± SEM. Two-way ANOVA with Bonferroni post hoc test. *p<0.05 vs. *Adam17$^{f/f}$*.

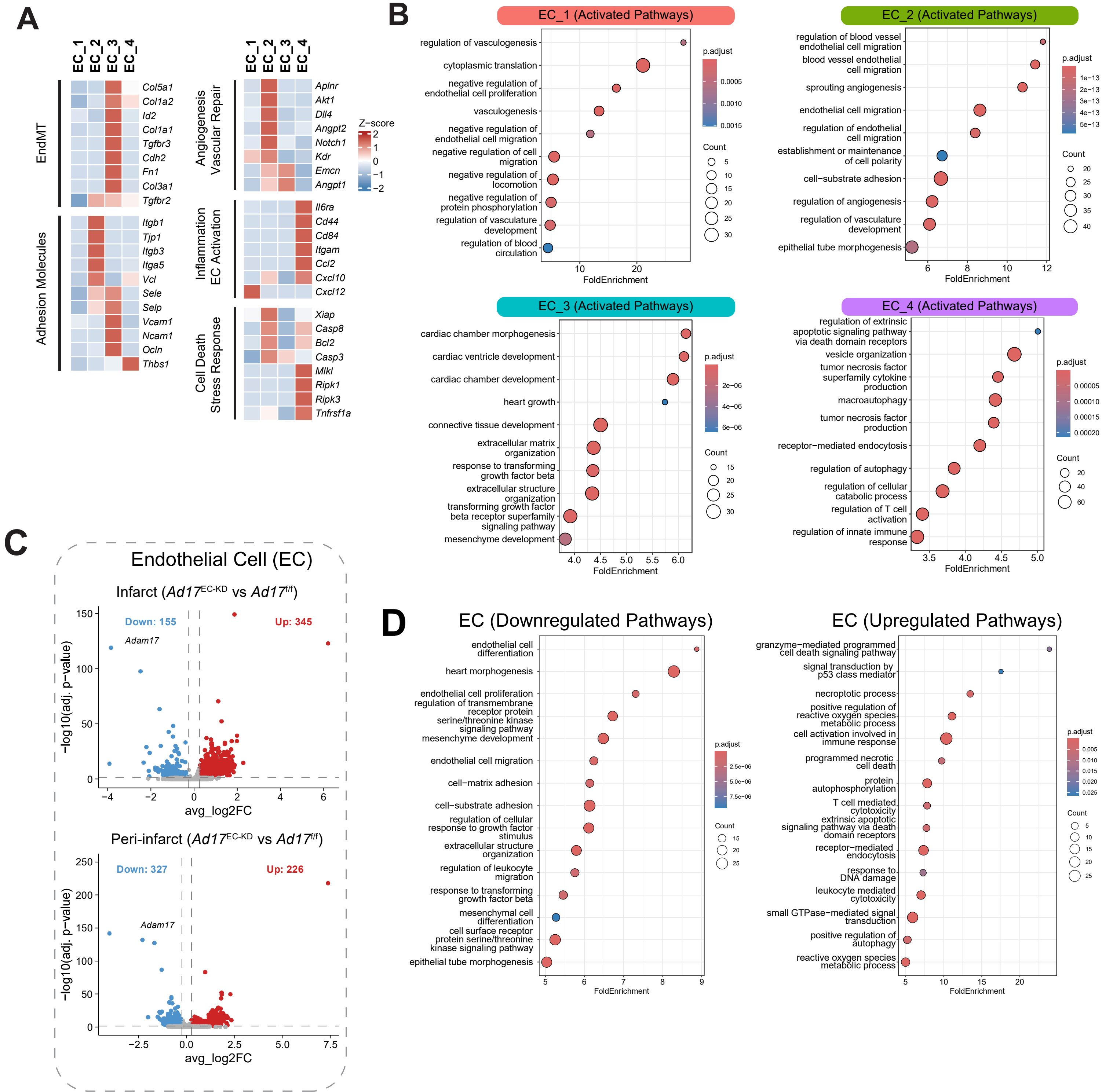

Figure S7: Endothelial ADAM17 deficiency reprograms endothelial transcription profile after MI. (A) Heatmap showing the expression of genes associated with angiogenesis, inflammation, endothelial-to-mesenchymal transition (EndMT), cell adhesion, and cell death across in distinct endothelial cell clusters(EC_1 to EC_4: naïve, proliferative, EndMT, and stressed ECs). **(B)** Functional enrichment analysis reveals EC_1 is associated with naïve endothelial identity, EC_2 with angiogenesis , EC_3 with endothelial-to-mesenchymal transition (EndMT), and EC_4 with inflammatory and cell death-related pathways. (**C)** Volcano plots of differentially expressed genes (DEGs) in endothelial cells from peri-infarct and infarct regions of *Adam17*$^{EC\text{-}KD}$ vs *Adam17*$^{f/f}$ hearts, highlighting downregulated (blue) and upregulated (red) transcripts. **(D)** Gene ontology enrichment analysis of significantly downregulated (left) and upregulated (right) pathways in endothelial cells from *Adam17*$^{EC\text{-}KD}$ vs *Adam17*$^{f/f}$ hearts, showing suppression of angiogenesis and adhesion programs and enrichment of stress and cell-death pathways.

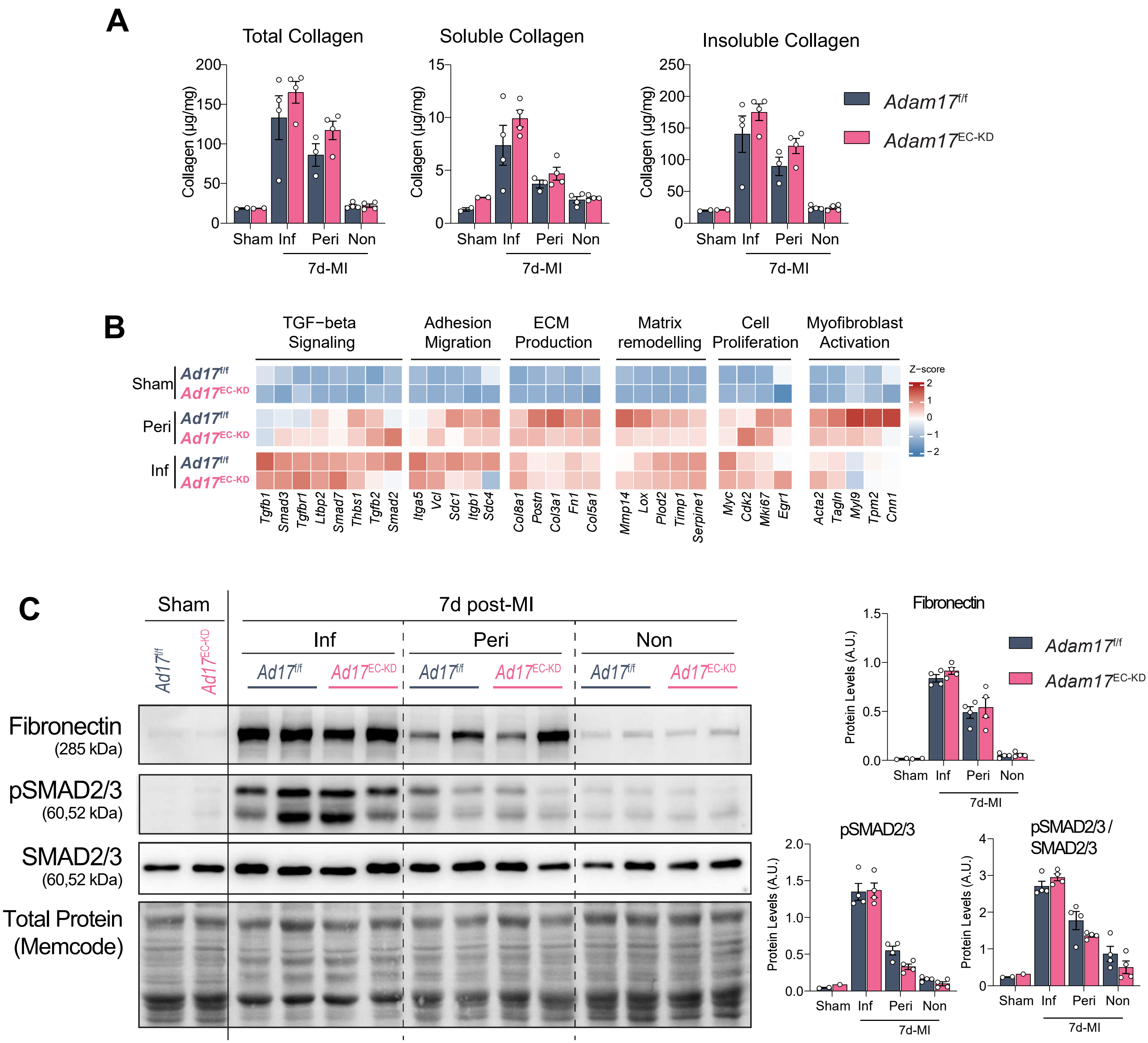

Figure S8: Comparable collagen processing and deposition, in *Adam17*$^{EC-KD}$ mice that survived to 7 days post-MI compared to parallel *Adam17*$^{f/f}$ mice. **(A)** Quantification of total, soluble and insoluble collagen content at 7 days post-MI in sham and post-MI hearts of each genotype. **(B)** Heatmap of genes associated with FB-like functions in *Adam17*$^{EC-KD}$ vs *Adam17*$^{f/f}$ hearts (based on snRNAseq data). **(C)** Representative immunoblot analysis and protein quantification phosphorylated and total SMAD2/3 signaling, and fibronectin expression in heart tissue at 7 days post-MI in *Adam17*$^{f/f}$ and *Adam17*$^{EC-KD}$ male mice. Averaged data are presented as Mean ± SEM. Statistical analysis was performed using two-way ANOVA with Bonferroni post hoc test. **p*<0.05 vs. *Adam17*$^{f/f}$.

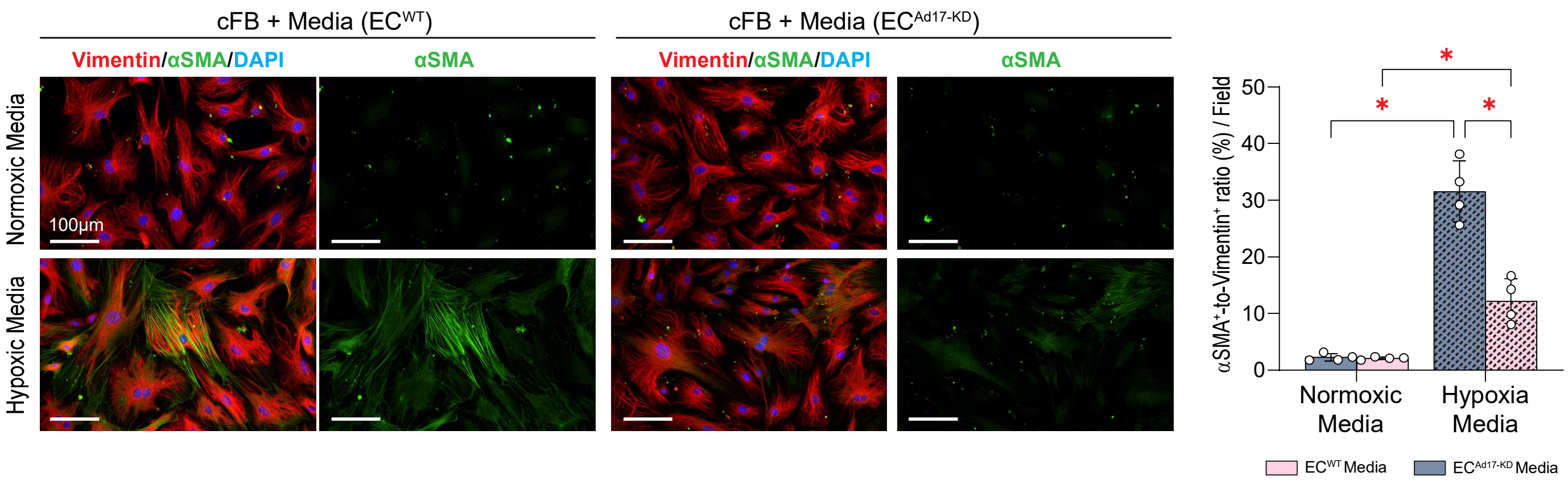

Figure S9: Conditioned media from hypoxia-treated *Adam17*-deficient ECs reduced activation of cardiac fibroblasts. Representative immunofluorescence staining of cardiac fibroblasts (cFB) treated with conditioned media from EC^{WT} or $EC^{Ad17\text{-}KD}$ cells cultured under normoxic or hypoxic conditions. Vimentin (red) marks fibroblasts, αSMA (green) indicates myofibroblast activation, and nuclei are stained with DAPI (blue) (n=4 independent experiments; 6-8 wells per condition). Data are presented as mean ± SEM. *$p < 0.05$.

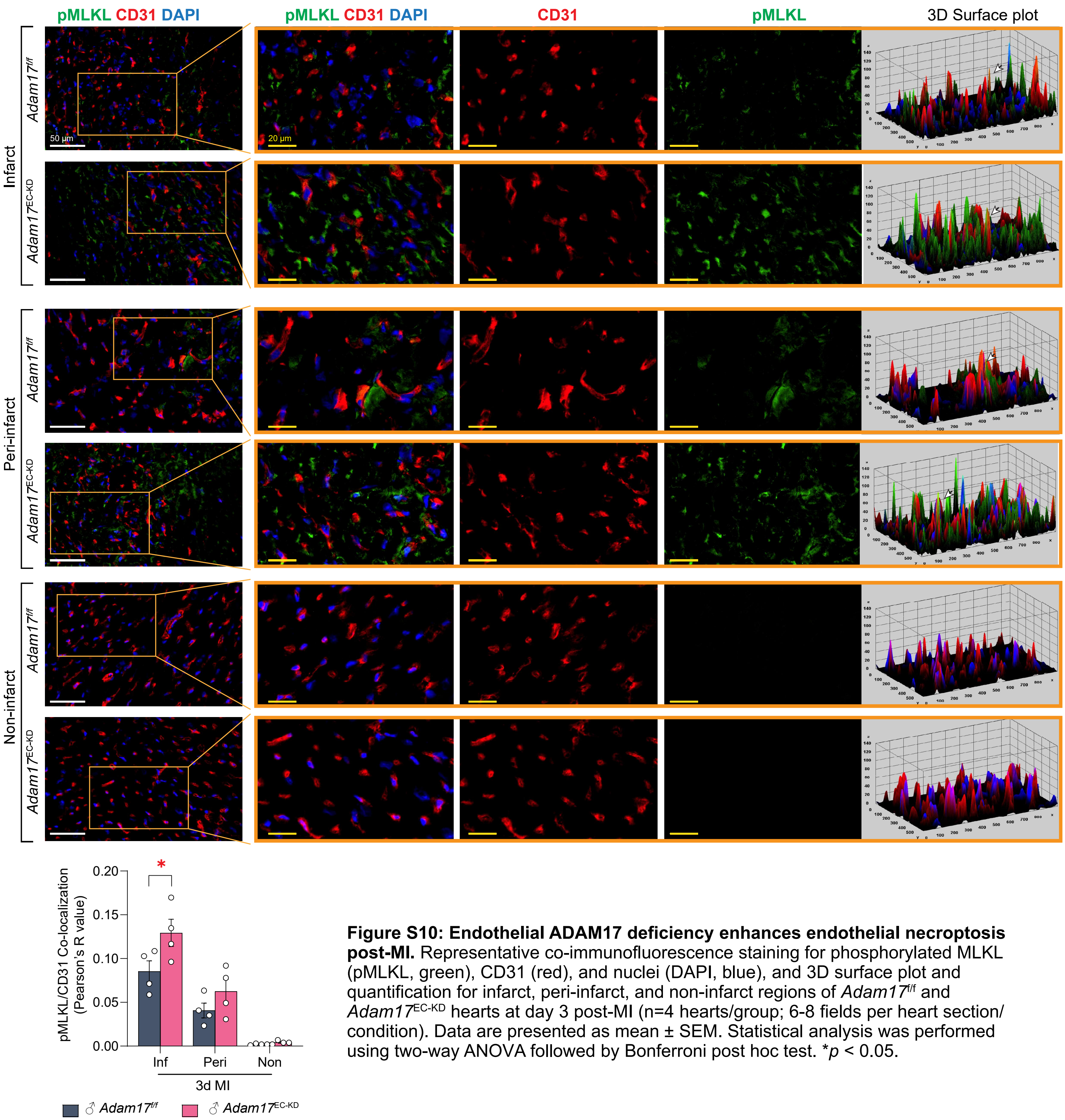

Figure S10: Endothelial ADAM17 deficiency enhances endothelial necroptosis post-MI. Representative co-immunofluorescence staining for phosphorylated MLKL (pMLKL, green), CD31 (red), and nuclei (DAPI, blue), and 3D surface plot and quantification for infarct, peri-infarct, and non-infarct regions of *Adam17*$^{f/f}$ and *Adam17*$^{EC\text{-}KD}$ hearts at day 3 post-MI (n=4 hearts/group; 6-8 fields per heart section/ condition). Data are presented as mean ± SEM. Statistical analysis was performed using two-way ANOVA followed by Bonferroni post hoc test. *$p < 0.05$.

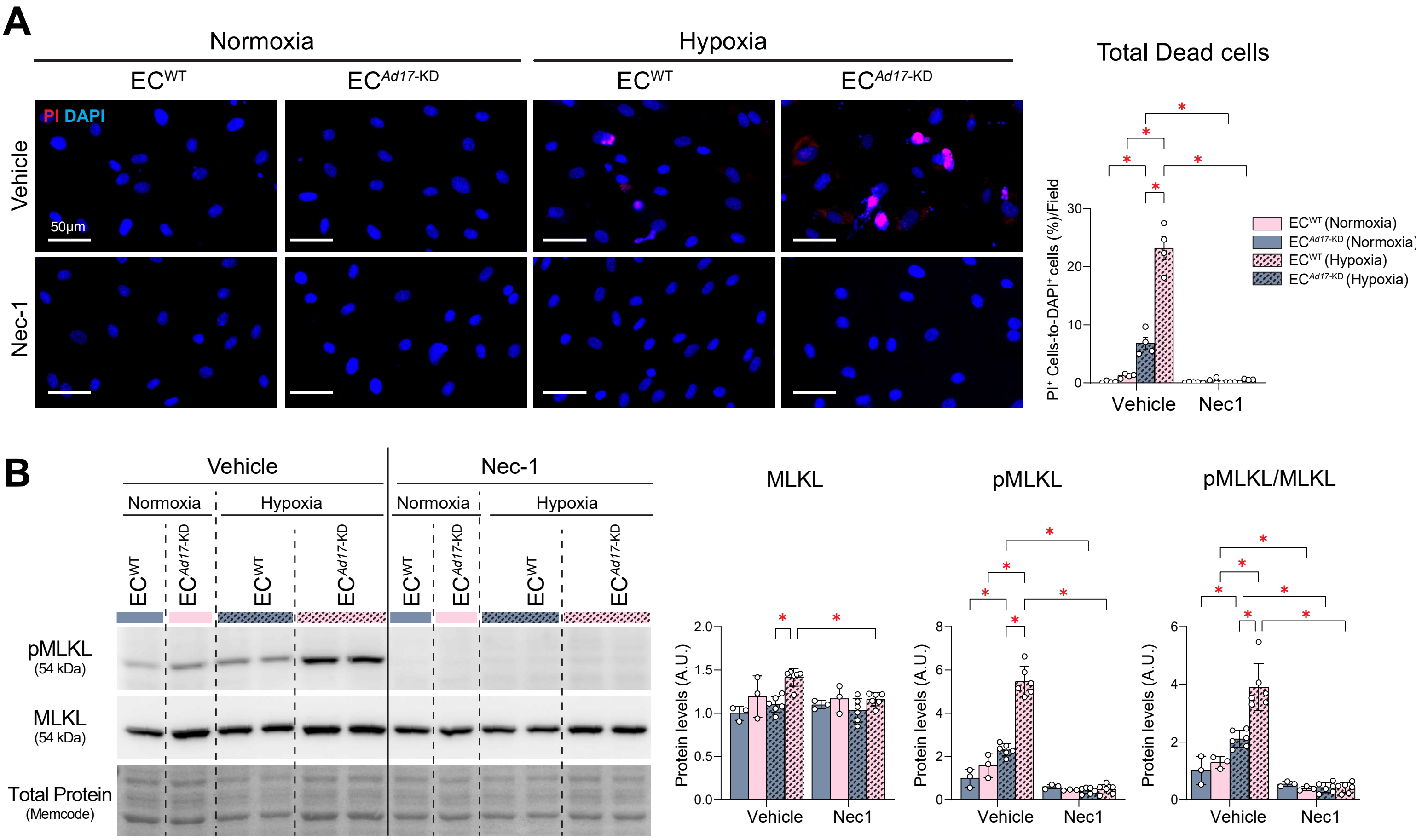

Figure S11: Hypoxia-induced cell death in ADAM17-deficient endothelial cells is inhibited by necrostatin-1. **(A)** Representative immunofluorescence images and quantification of endothelial cells with wild-type ADAM17 (EC^{WT}) or ADAM17 knockdown ($EC^{Ad17\text{-}KD}$) cultured under normoxia (21% O_2) or hypoxia (1% O_2) and treated with vehicle or necrostatin-1 (Nec-1) (n=2 independent experiments; 6-8 fields per condition). Dead cells were identified by propidium iodide (PI, red) and nuclei counterstained with DAPI (blue). **(B)** Representative immunoblots and quantification of MLKL, phosphorylated MLKL (pMLKL), and the pMLKL/MLKL ratio in EC^{WT} and $EC^{Ad17\text{-}KD}$ in similar cohorts. Data are presented as mean ± SEM. Statistical analysis was performed using two-way ANOVA followed by Bonferroni post hoc test. *$p < 0.05$.

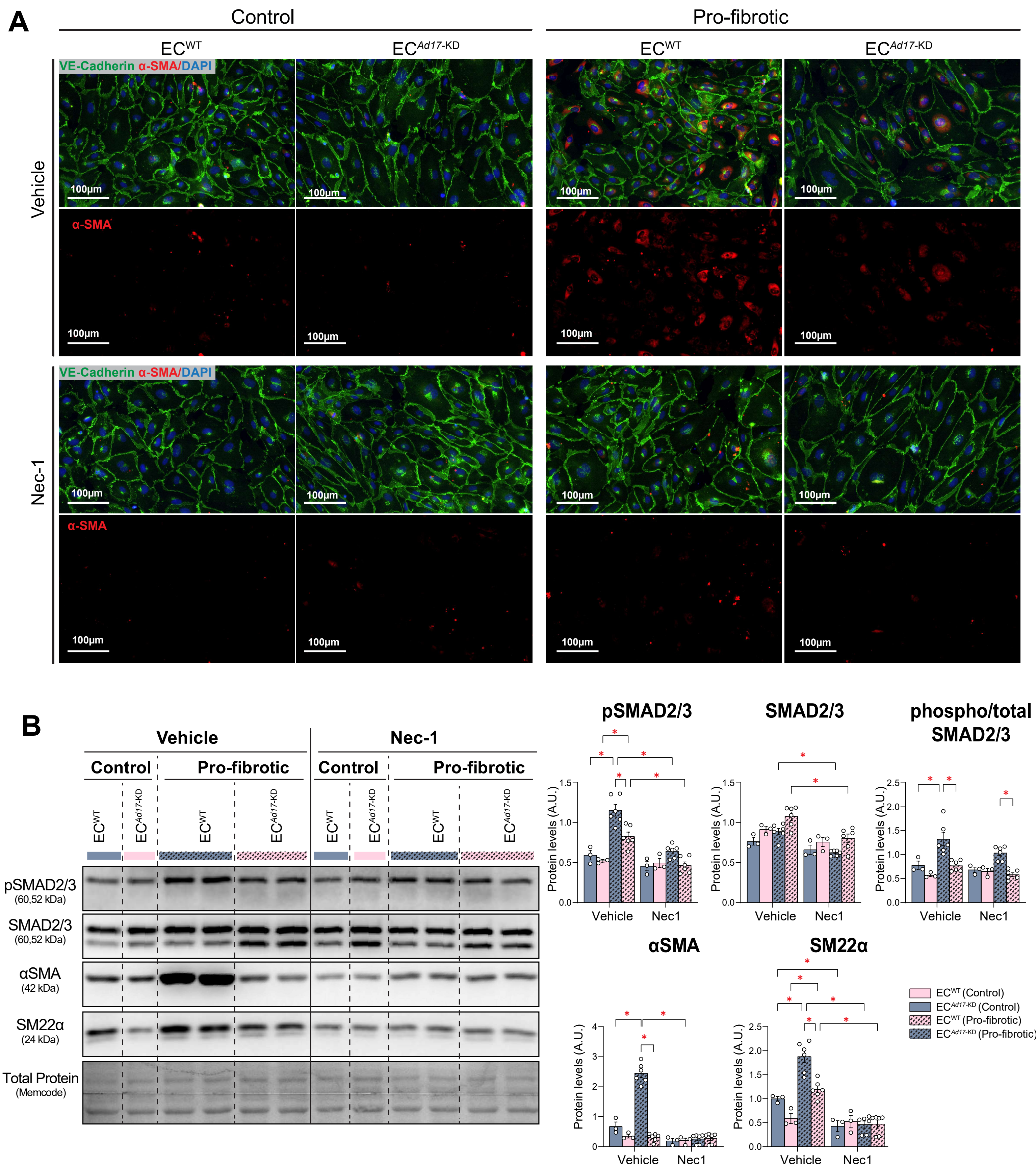

Figure S12: Loss of endothelial ADAM17 suppresses EndMT in pro-fibrotic conditions, which is mediated by necroptosis. (A) Representative co-immunofluorescence staining of VE-cadherin (green), αSMA (red), and DAPI (blue) in EC^{WT} and $EC^{Ad17\text{-}KD}$ under control (normoxia+TGFβ1) or pro-fibrotic (hypoxia+TGFβ1) conditions treated with vehicle or necrostatin-1(Nec-1, 25μM) (n=2 independent experiments; 6-8 fields per condition). **(B)** Representative immunoblots and averaged quantification of phospho- and total SMAD2/3, αSMA (alpha-smooth muscle actin), and SM22α (smooth muscle protein 22-alpha). Pro-fibrotic stimulation increased SMAD2/3 phosphorylation and mesenchymal markers (αSMA, Sm22α) in EC^{WT} but not in $EC^{Ad17\text{-}KD}$. Nec-1 reduced pSMAD2/3 and mesenchymal marker expression across all conditions. Averaged data are mean ± SEM. Statistical analysis was performed using two-way ANOVA followed by Bonferroni post-hoc test. *p<0.05 for indicated groups.

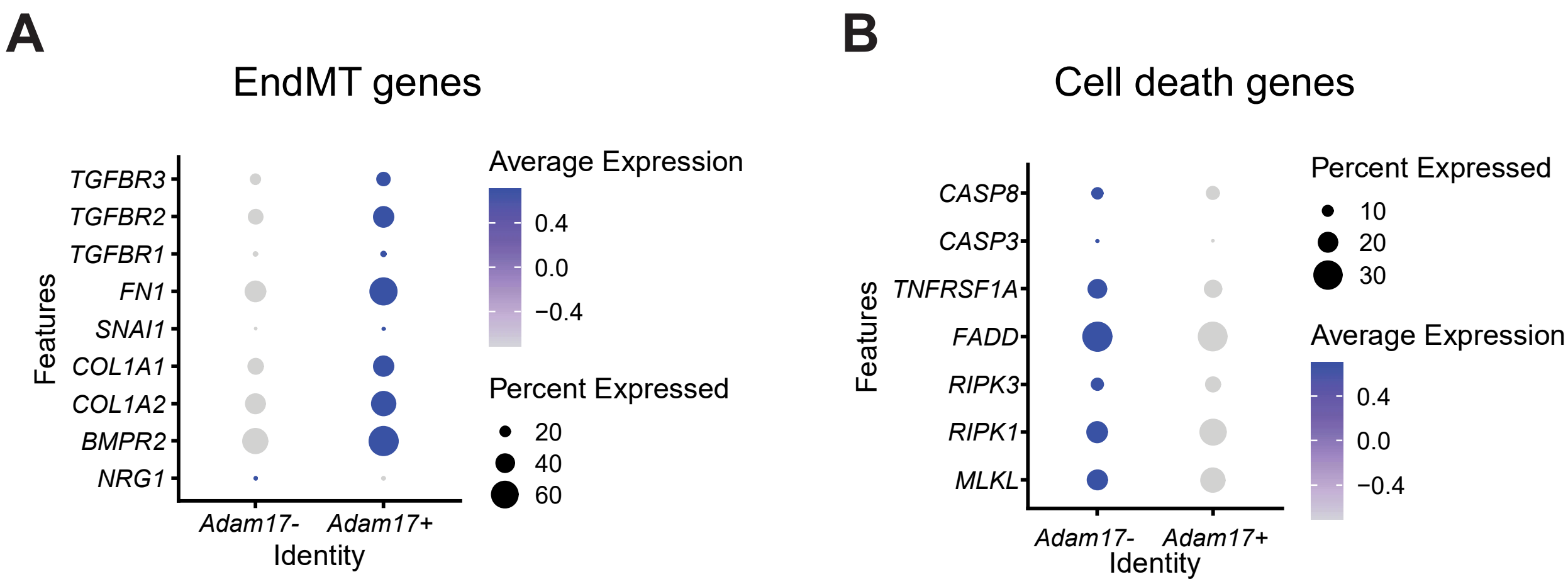

Figure S13: *Adam17* expression is posively related to EndMT and negatively to cell death in human cardiac endothelial cell. Expression of genes related to EndMT is higher in *Adam17*-positive (*Adam17*+) endothelial cells **(A)**, whereas expression of cell death- and necroptosis-related genes is higher in *Adam17*-negative (*Adam17*-) endothelial cells **(B)** from the ischemic myocardium of patients with ischemic cardiomyopathy (Publicly available single-cell RNA sequencing dataset from Kuppe *et al.*, 2022).
