# Supplementary material for: Necroptosis is a key contributor to impaired cardiac repair following myocardial infarction
Source: Cell Death Dis. 2026 Jun 15;17(1):751. doi: 10.1038/s41419-026-08979-5 (PMC13500855; doi:10.1038/s41419-026-08979-5)

Figure 3A: VCAM1

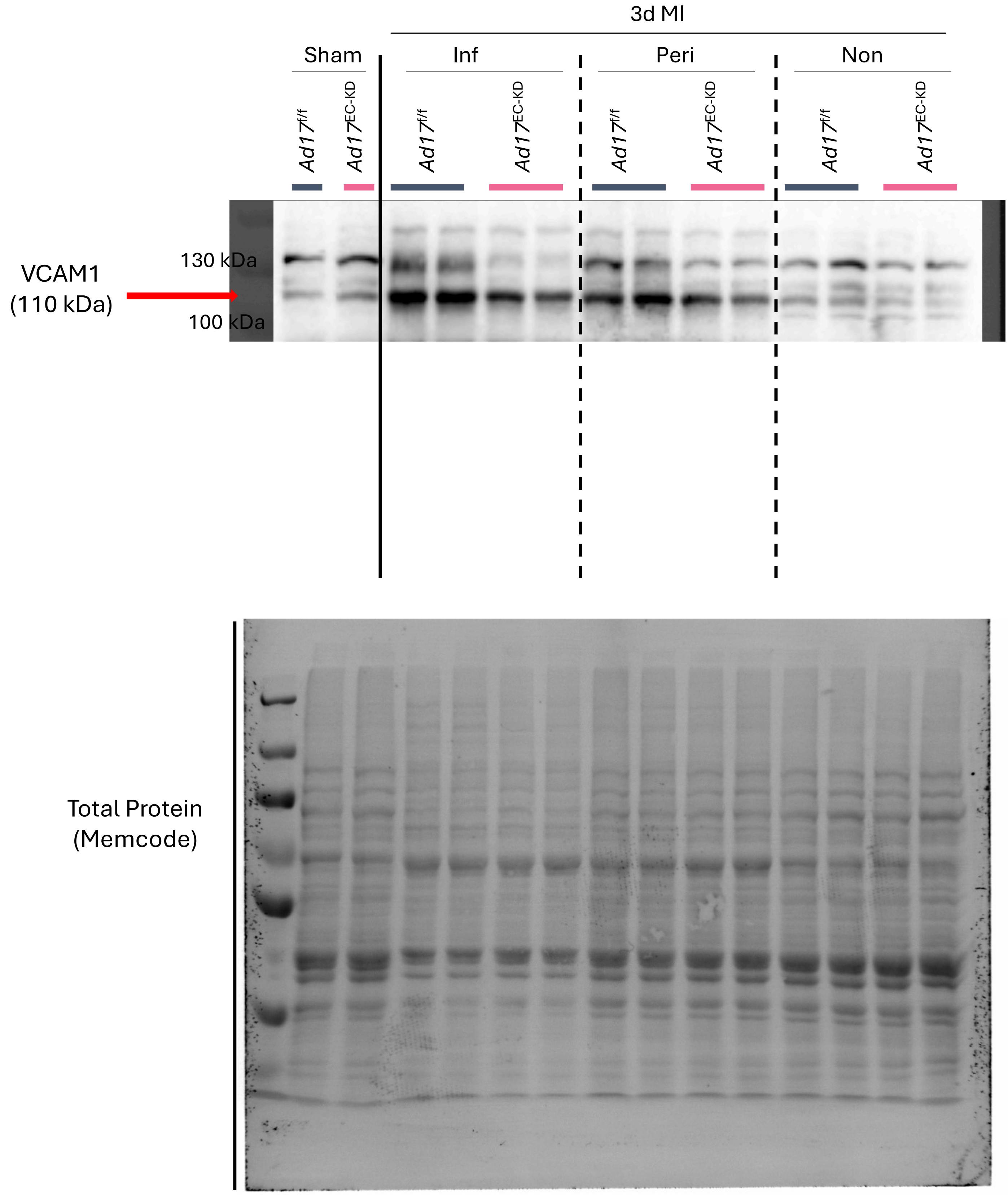

Figure 3A: ICAM1

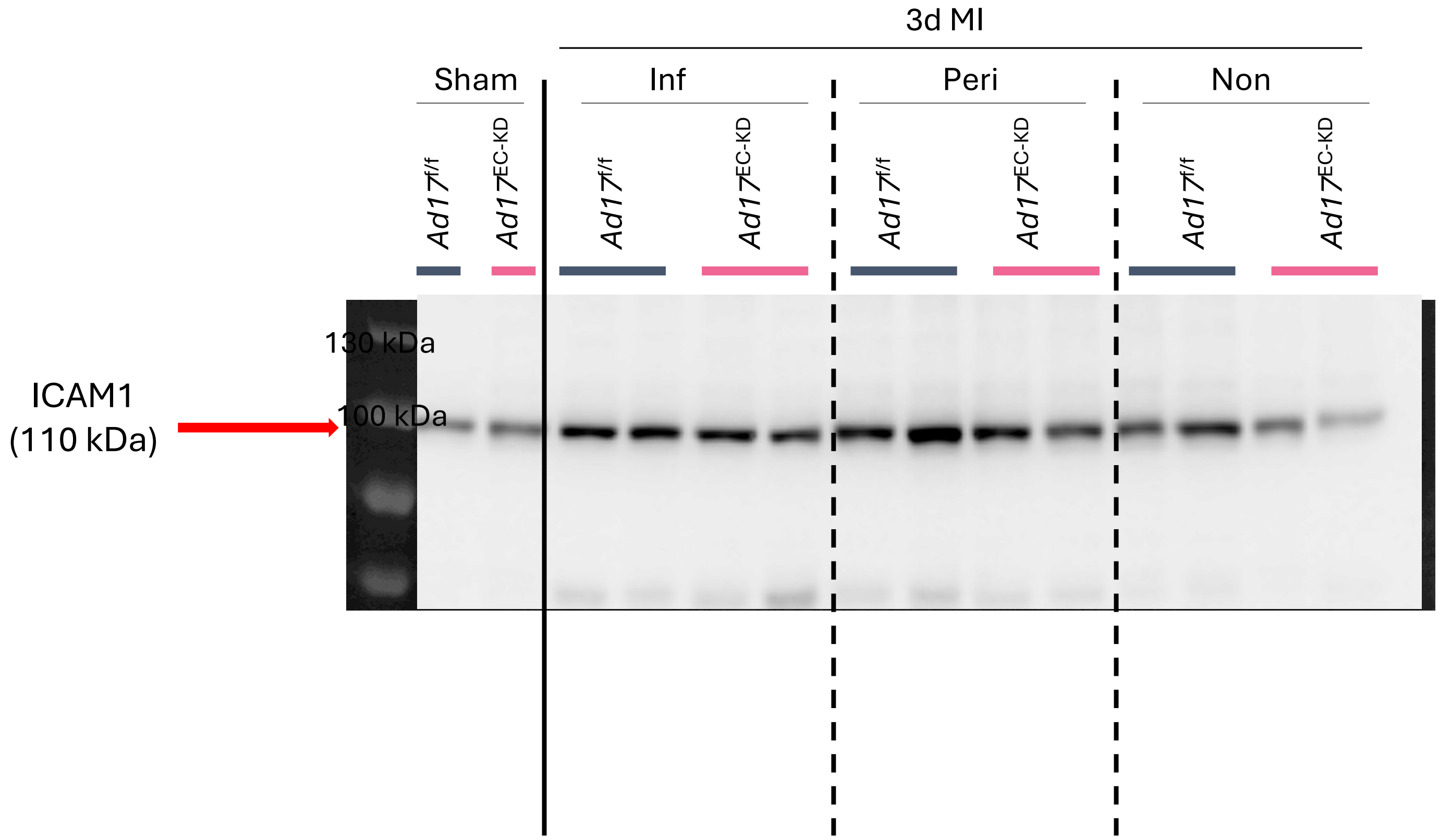

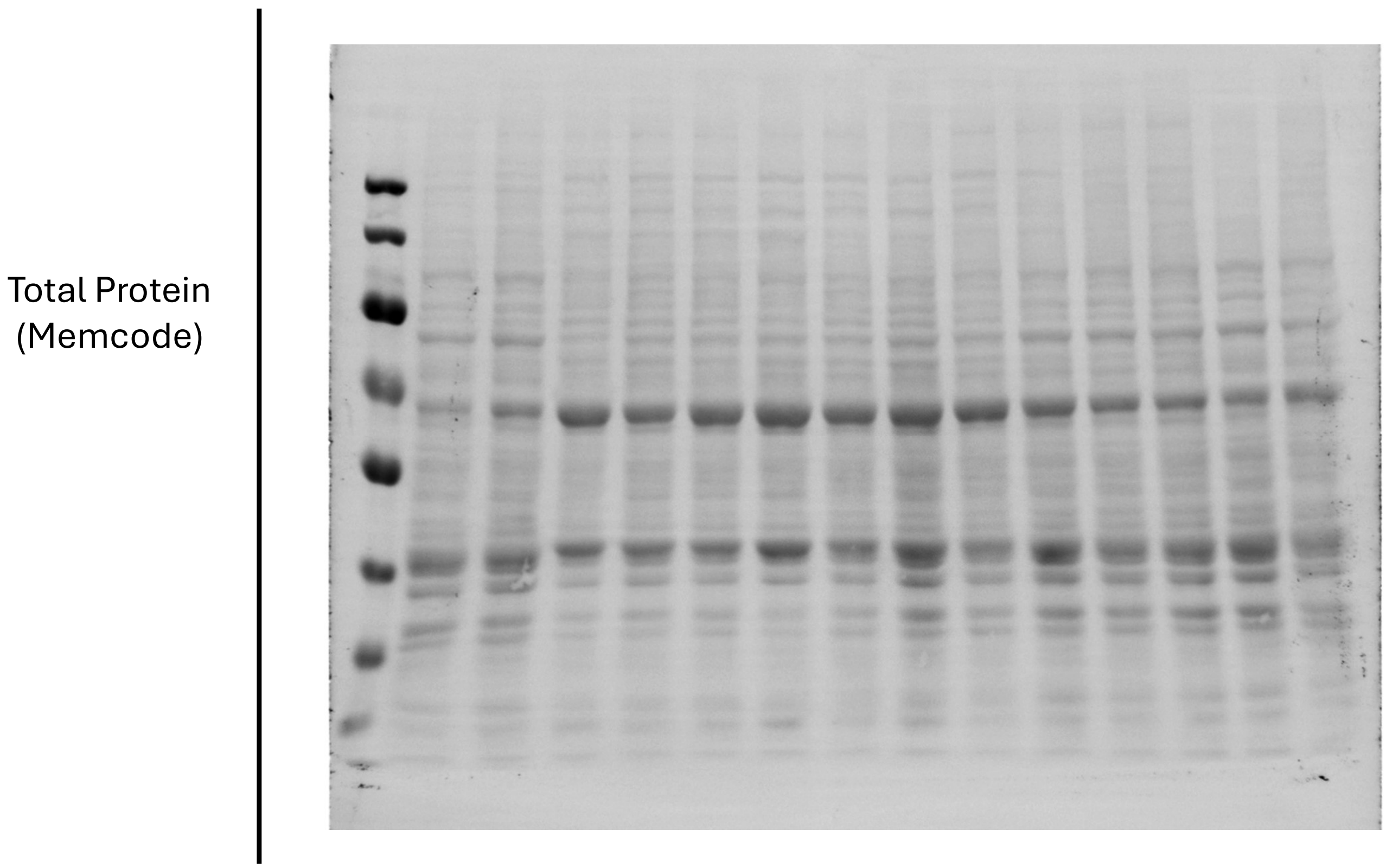

Figure 3A: JAM-A

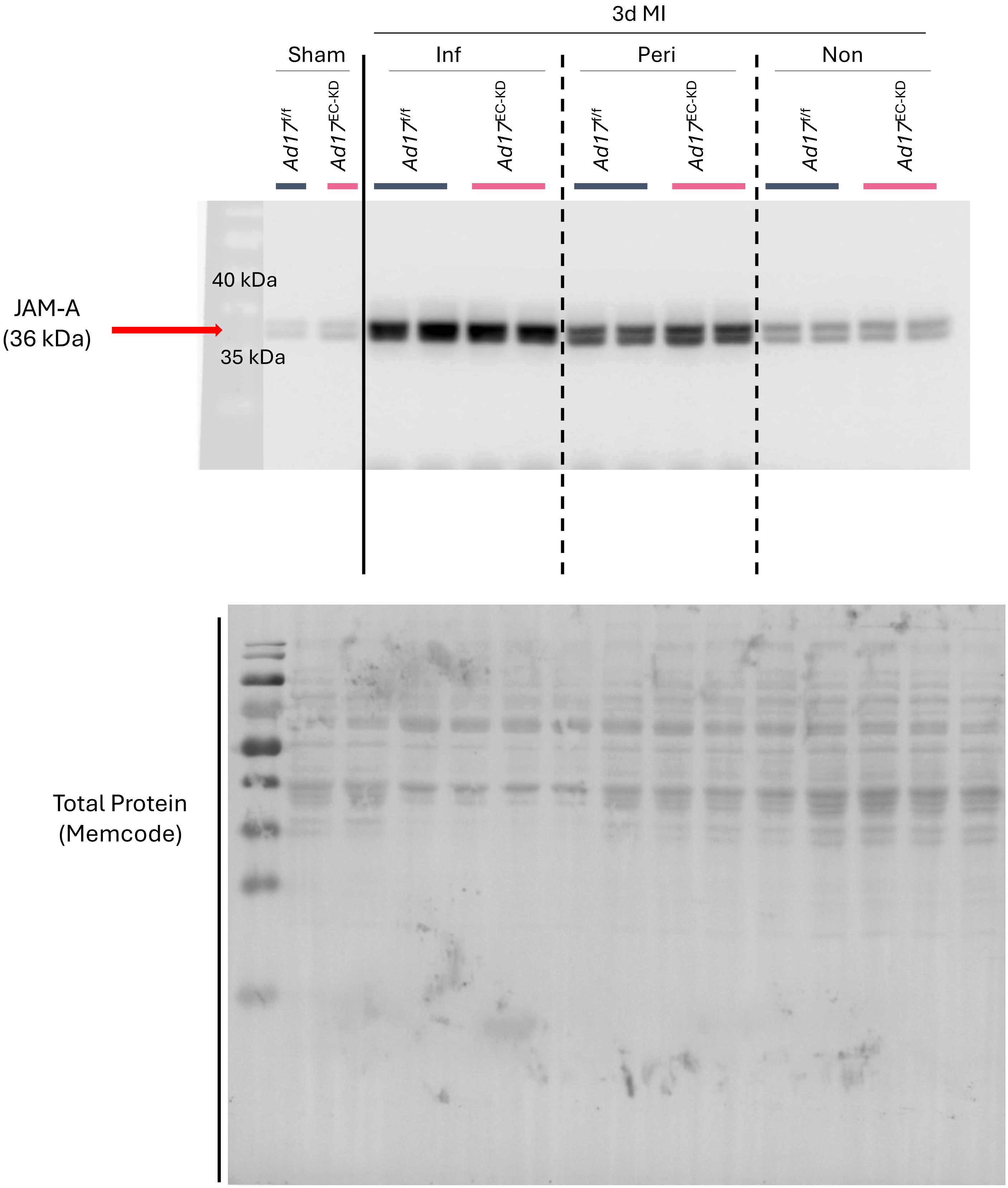

Figure 3A: CXCR3

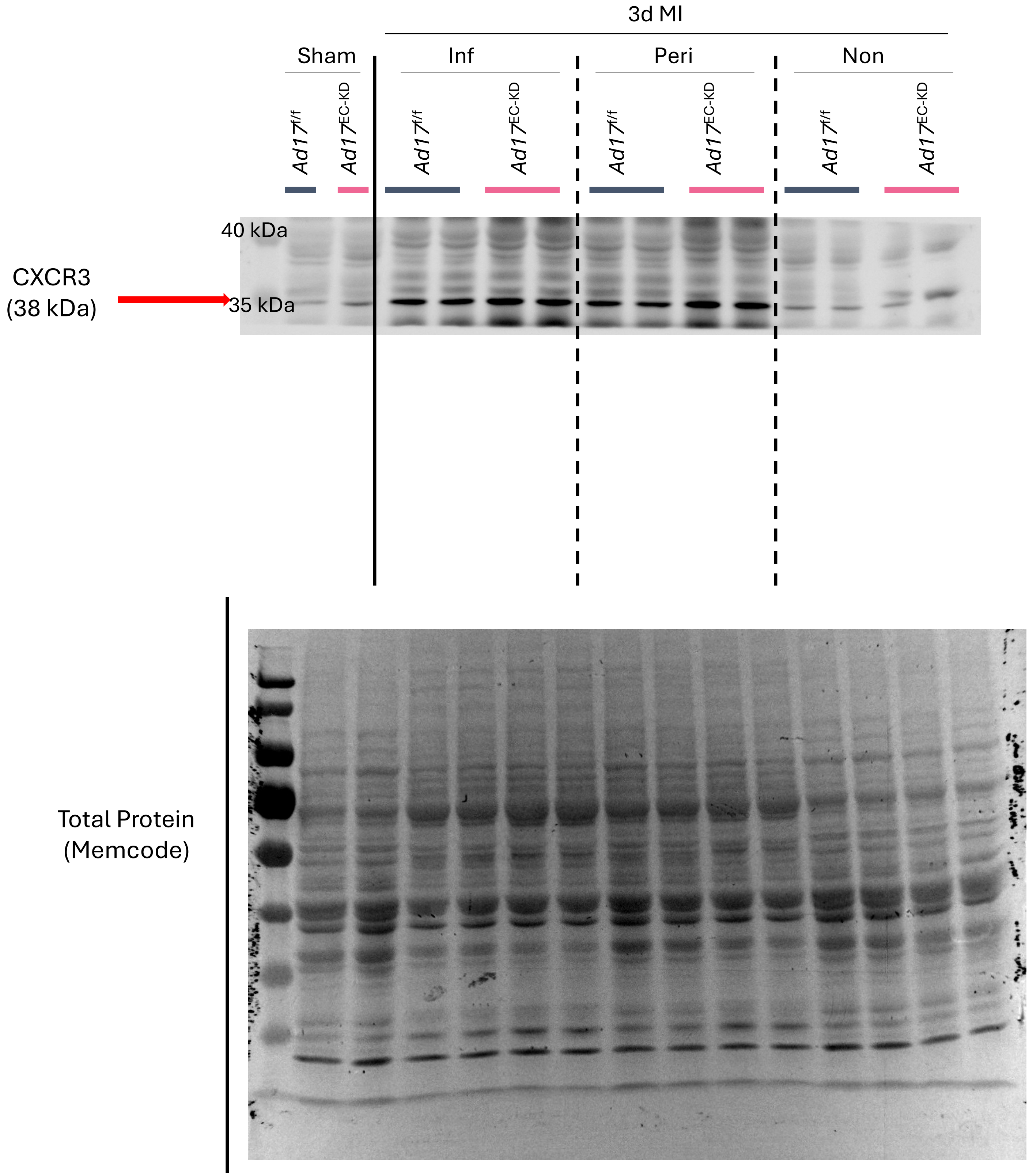

Figure 3A: GZMB

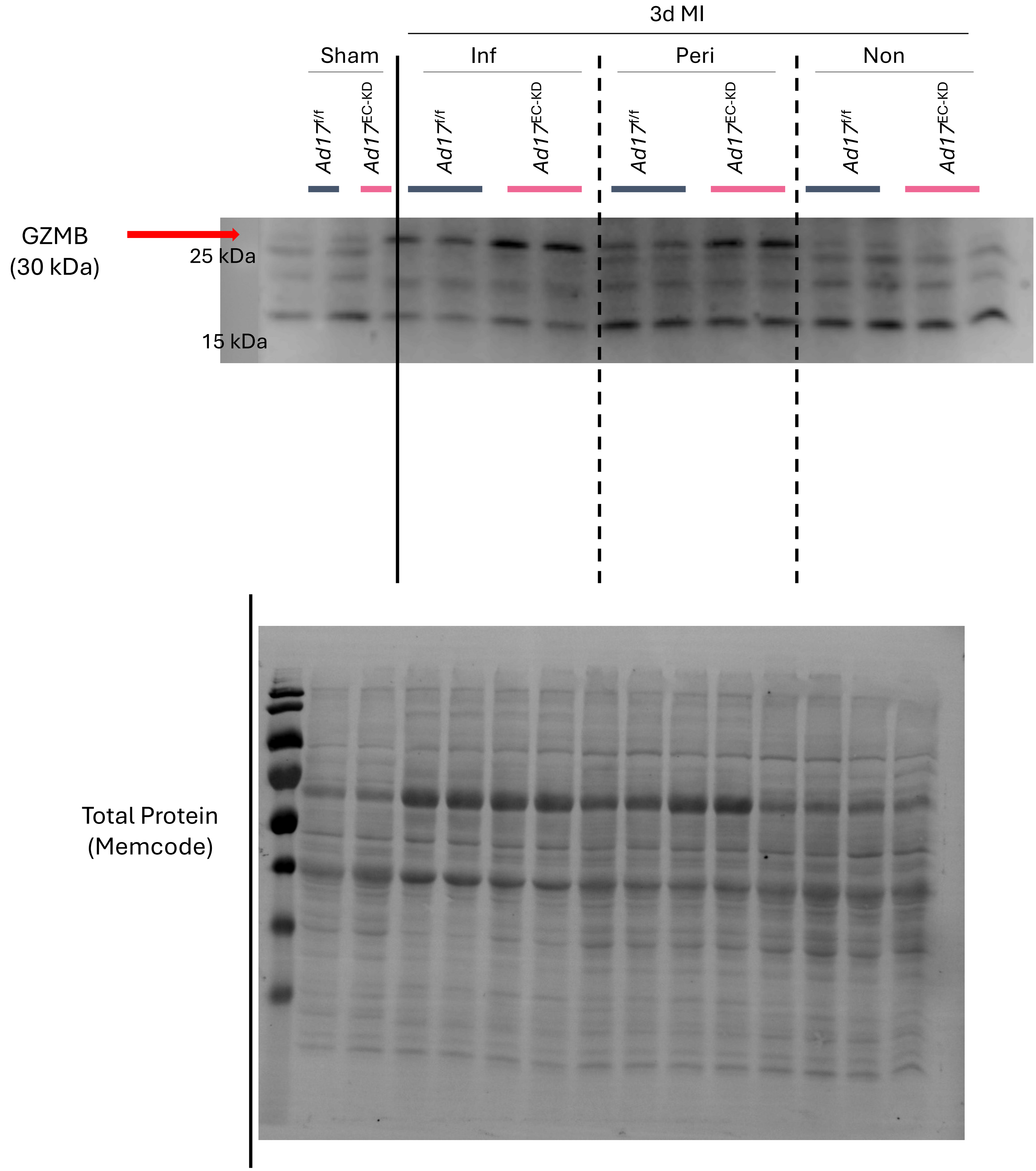

Figure 3B: ICAM1

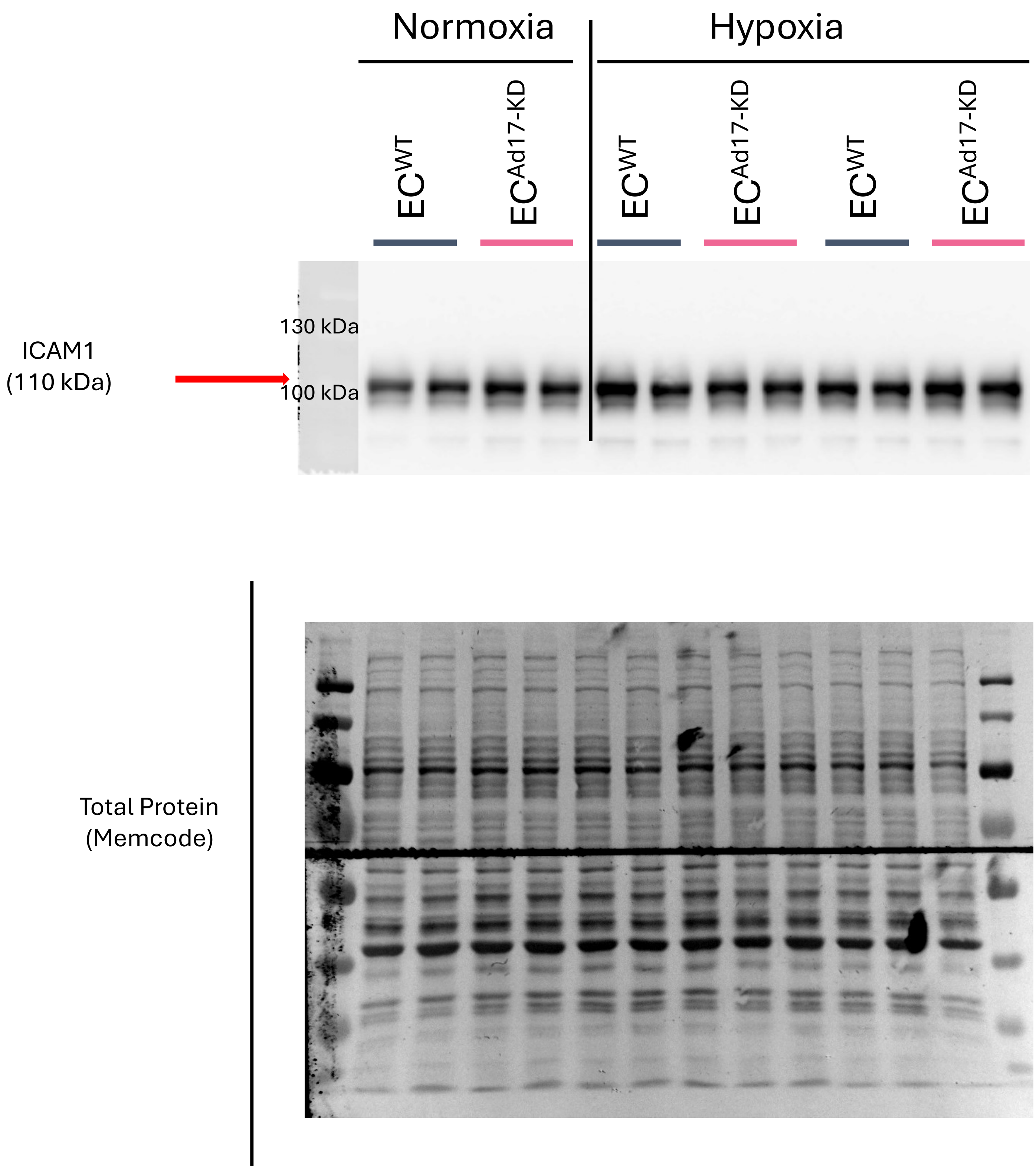

Figure 3B: VCAM1

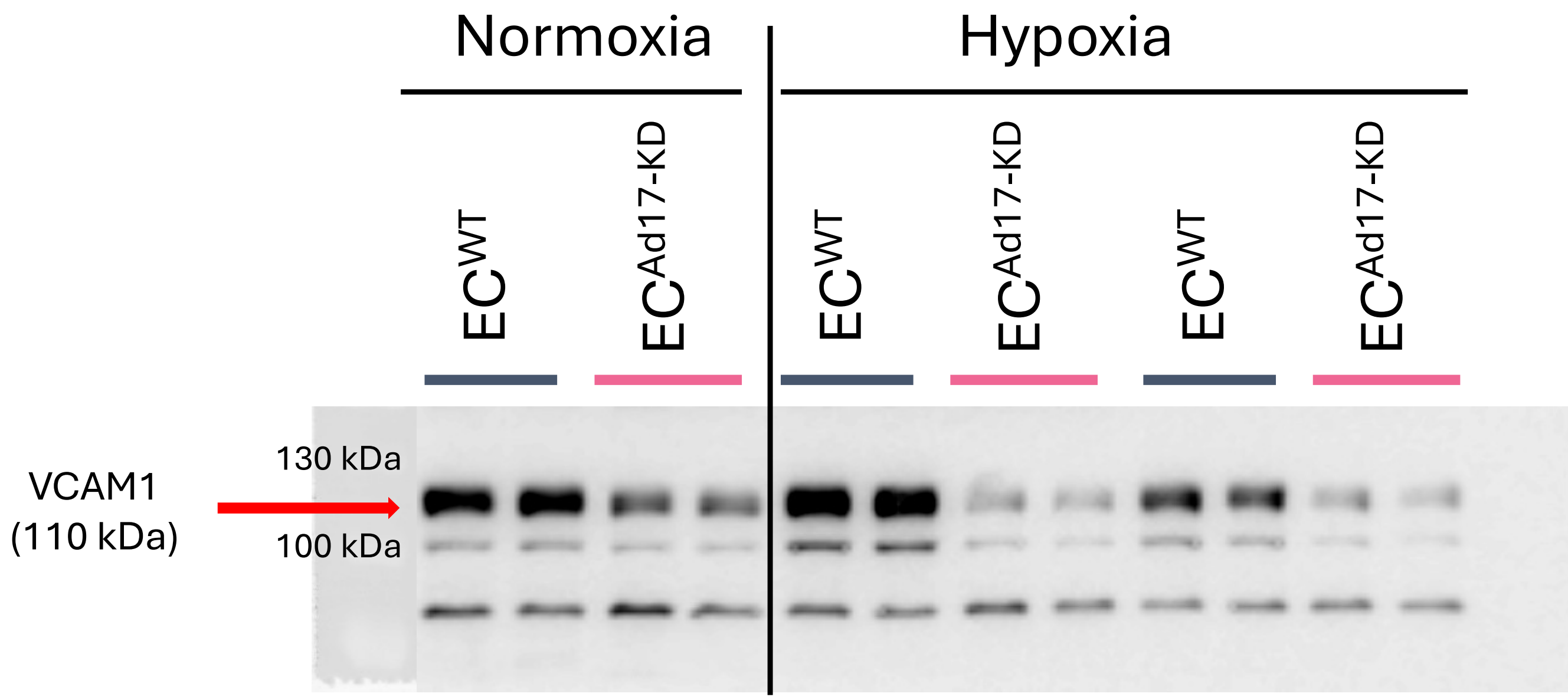

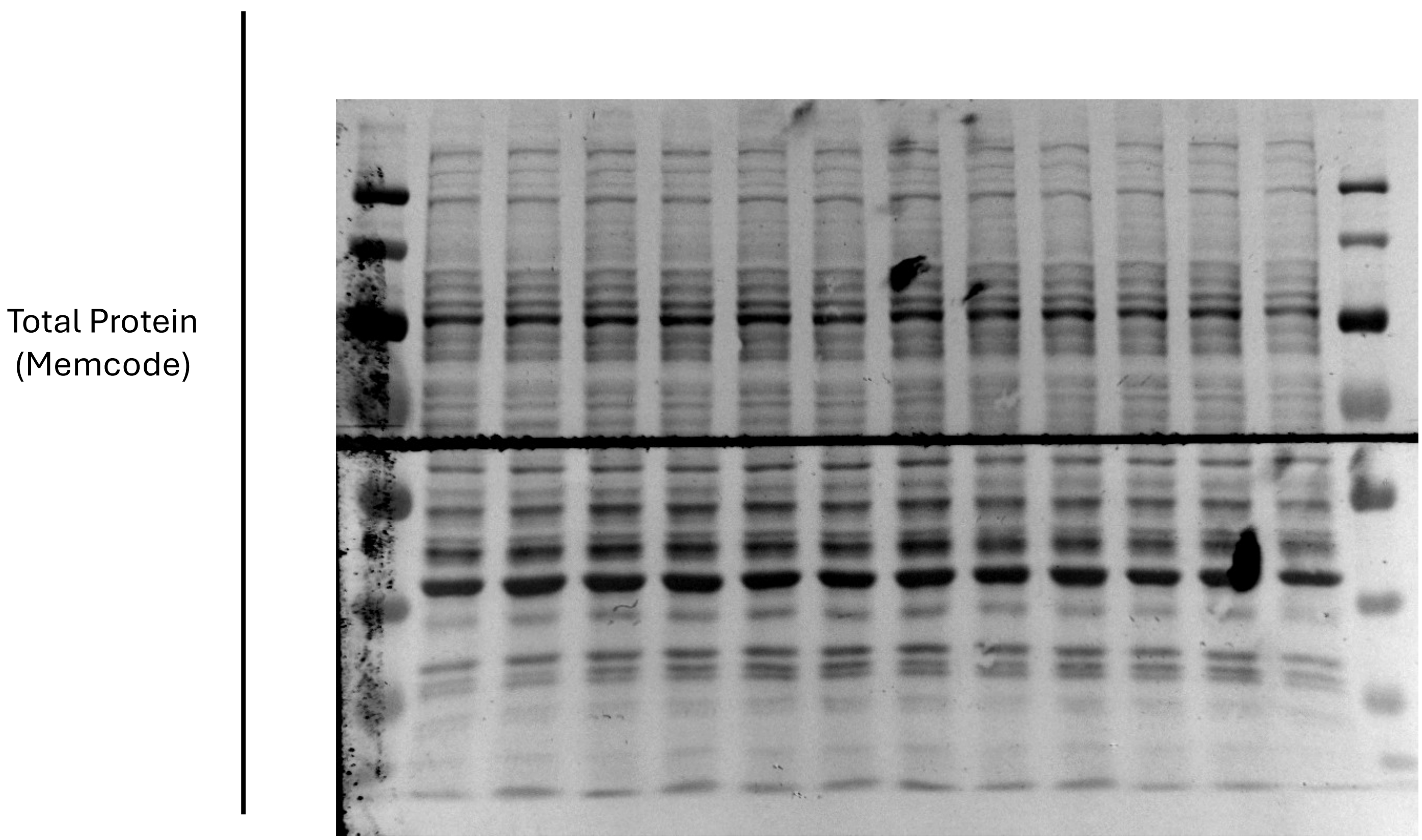

Figure 3B: JAMA

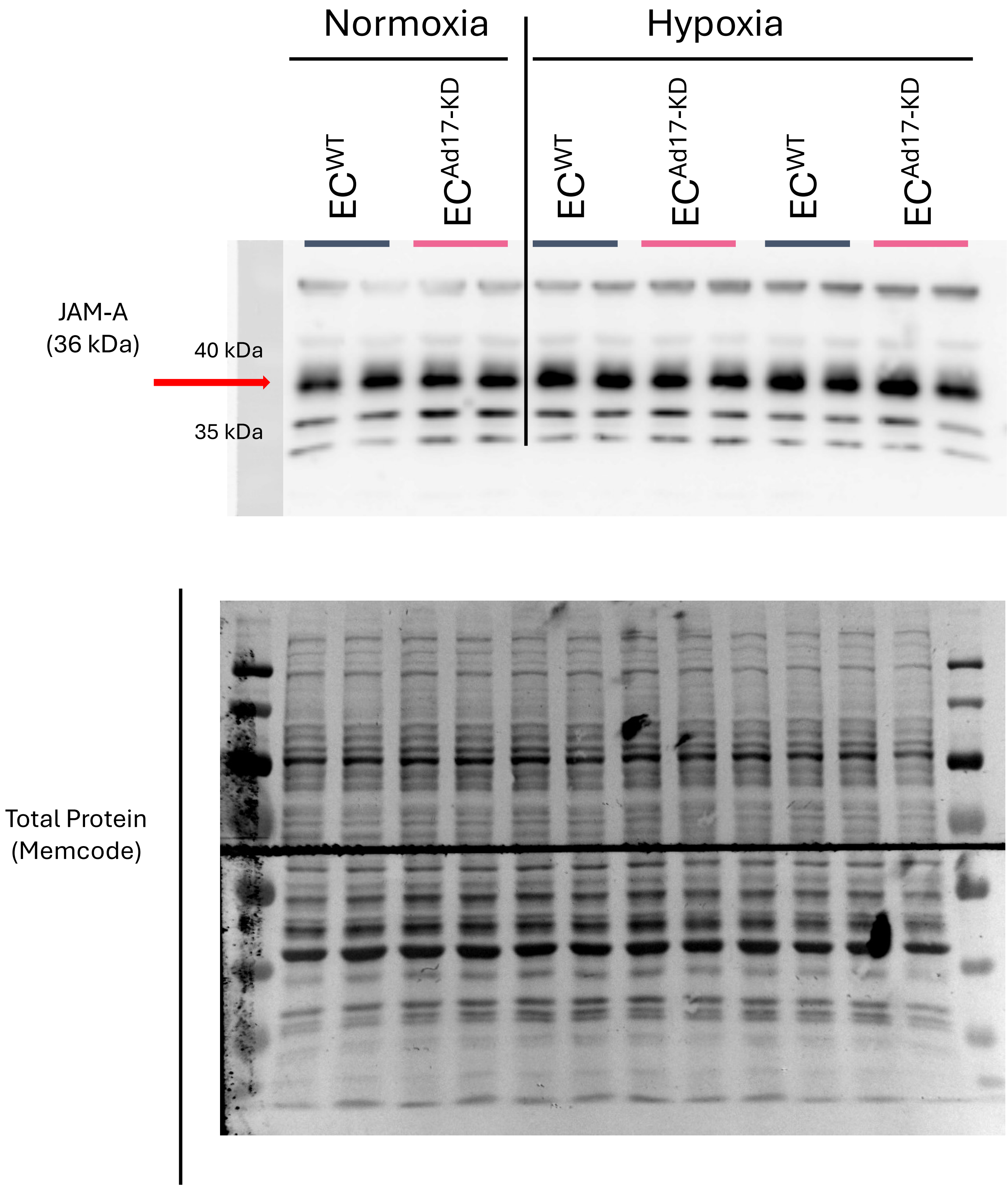

Figure 3B: CXCR3

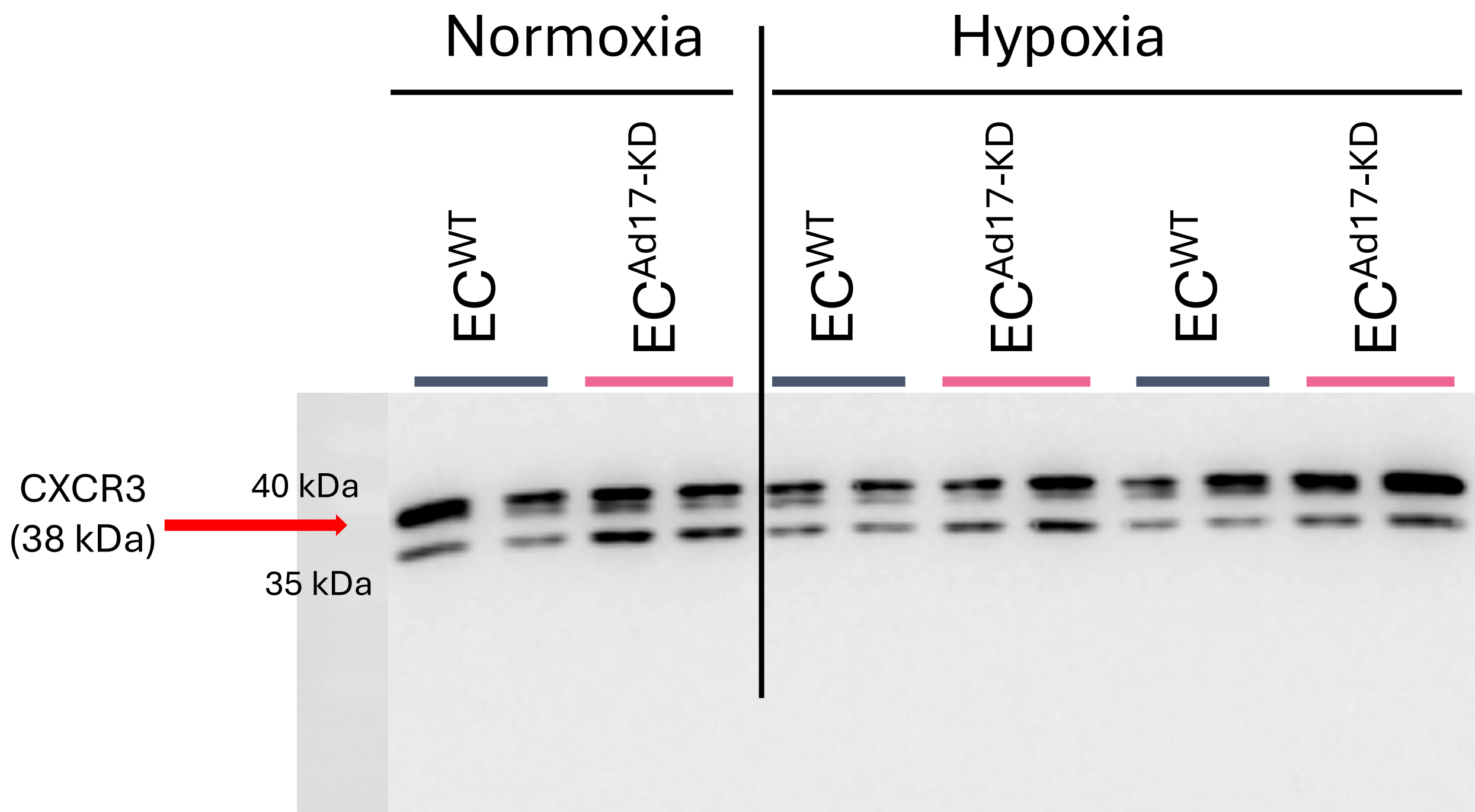

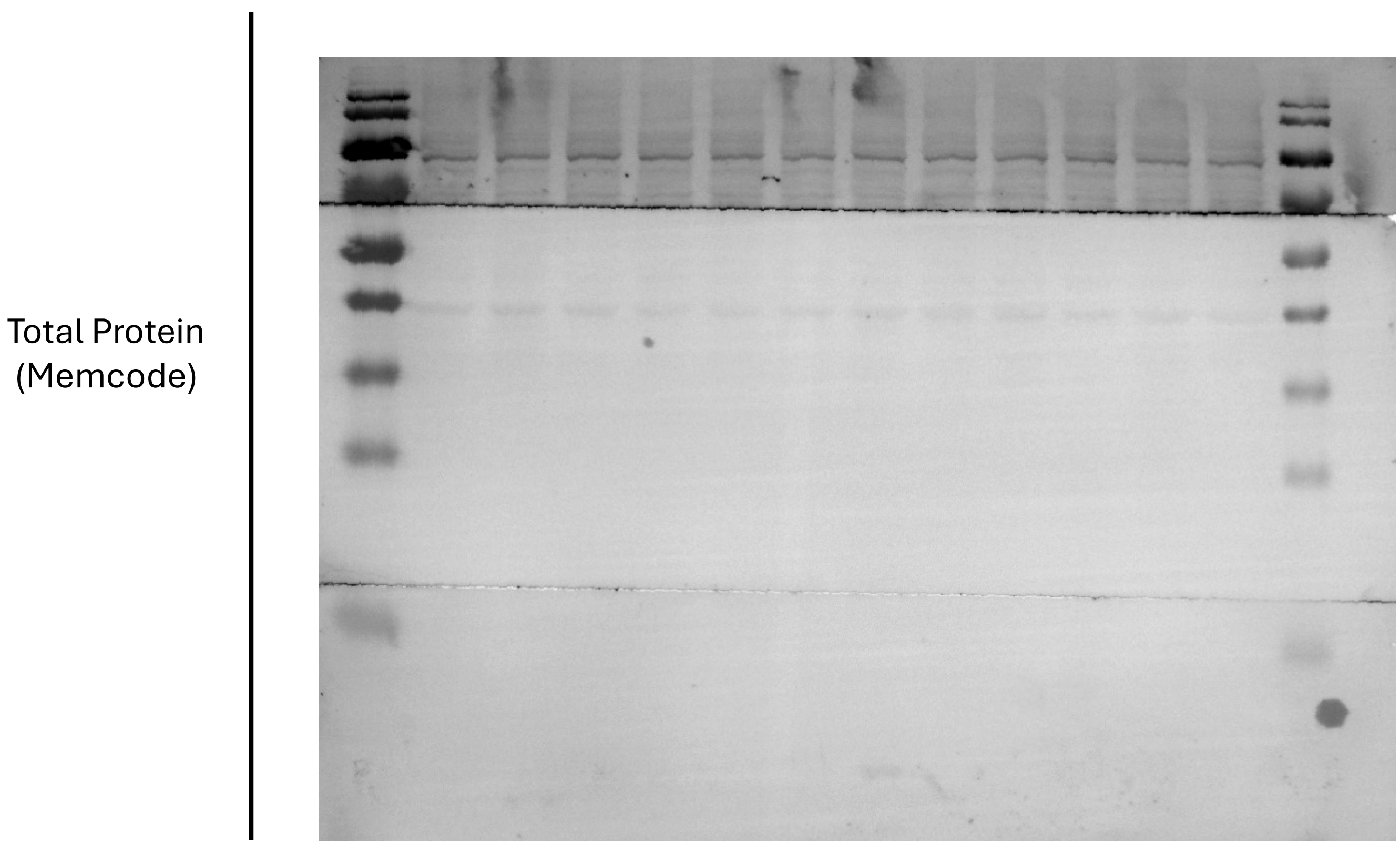

Figure 4C: pVEGFR2 + VEGFR2

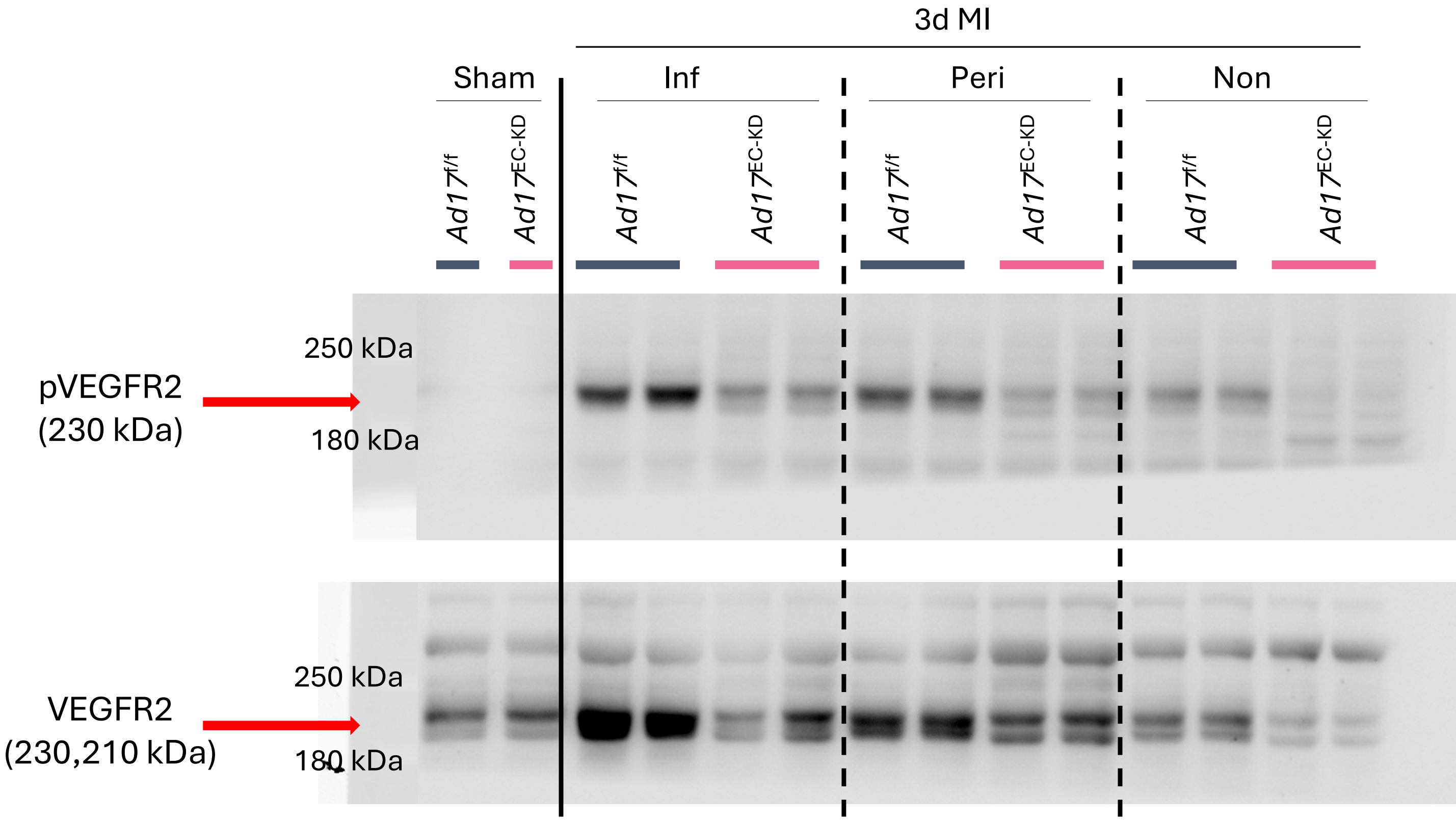

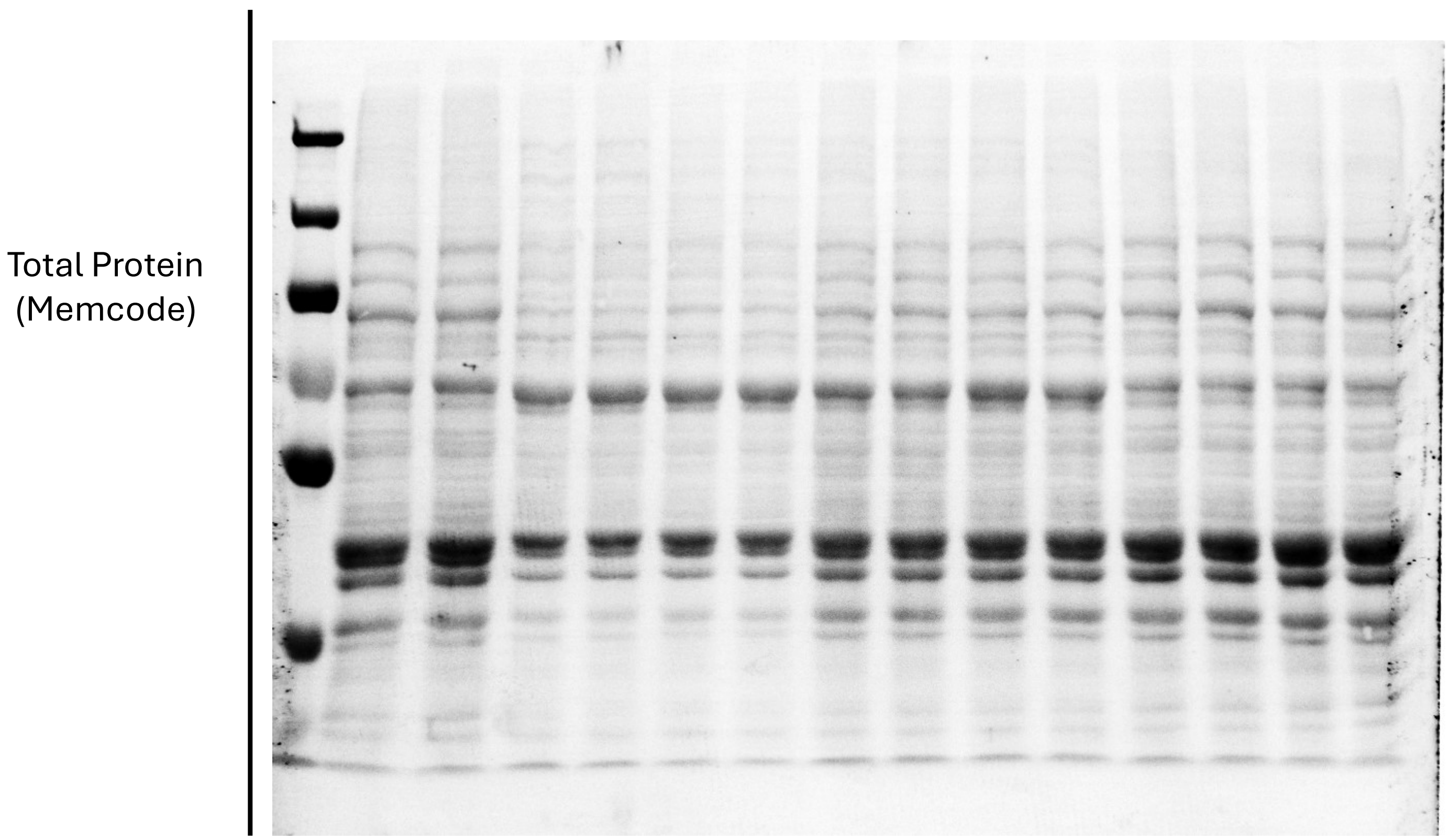

Figure 4C: pAKT1/2 + AKT1/2

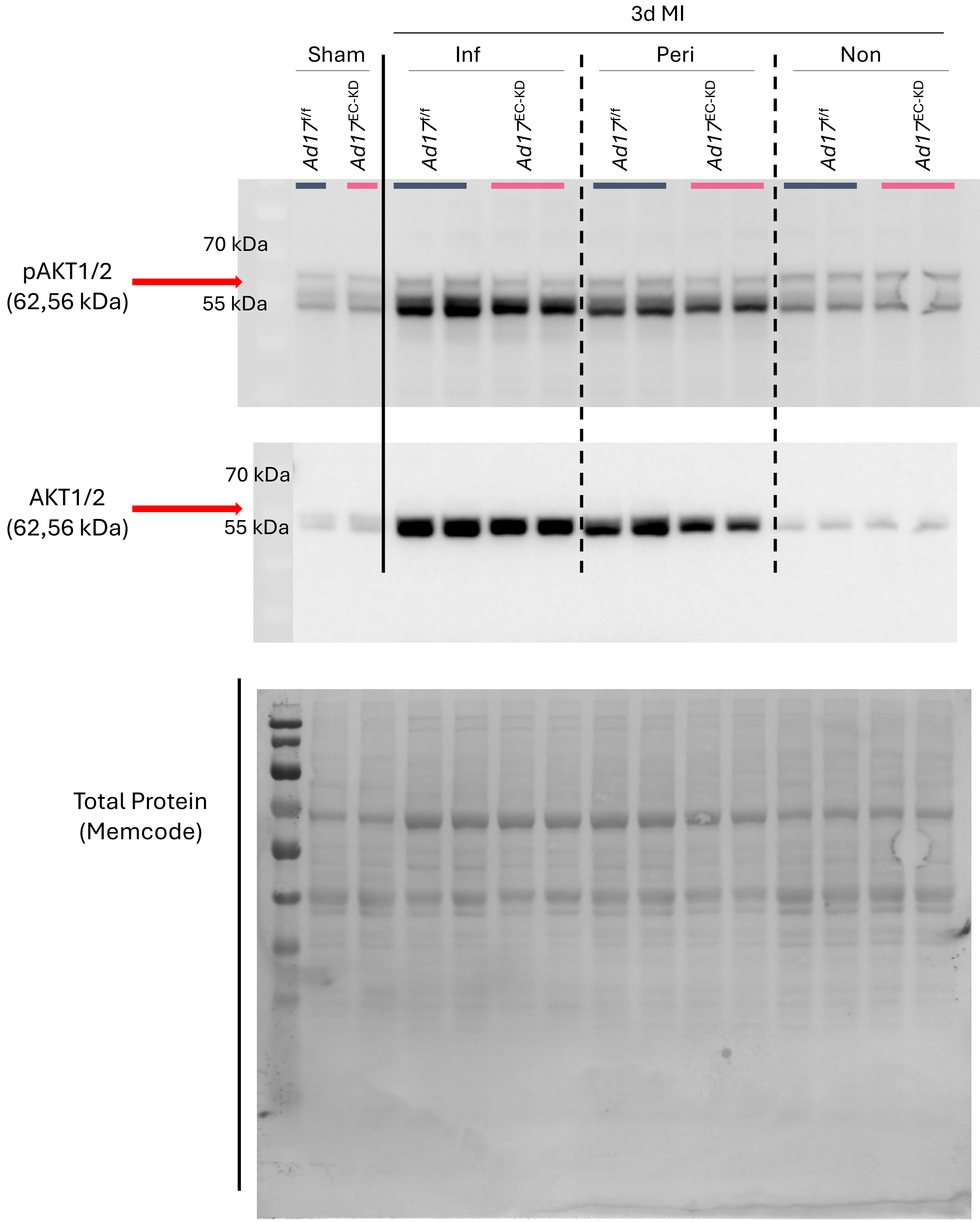

Figure 4E: pVEGFR2 + VEGFR2

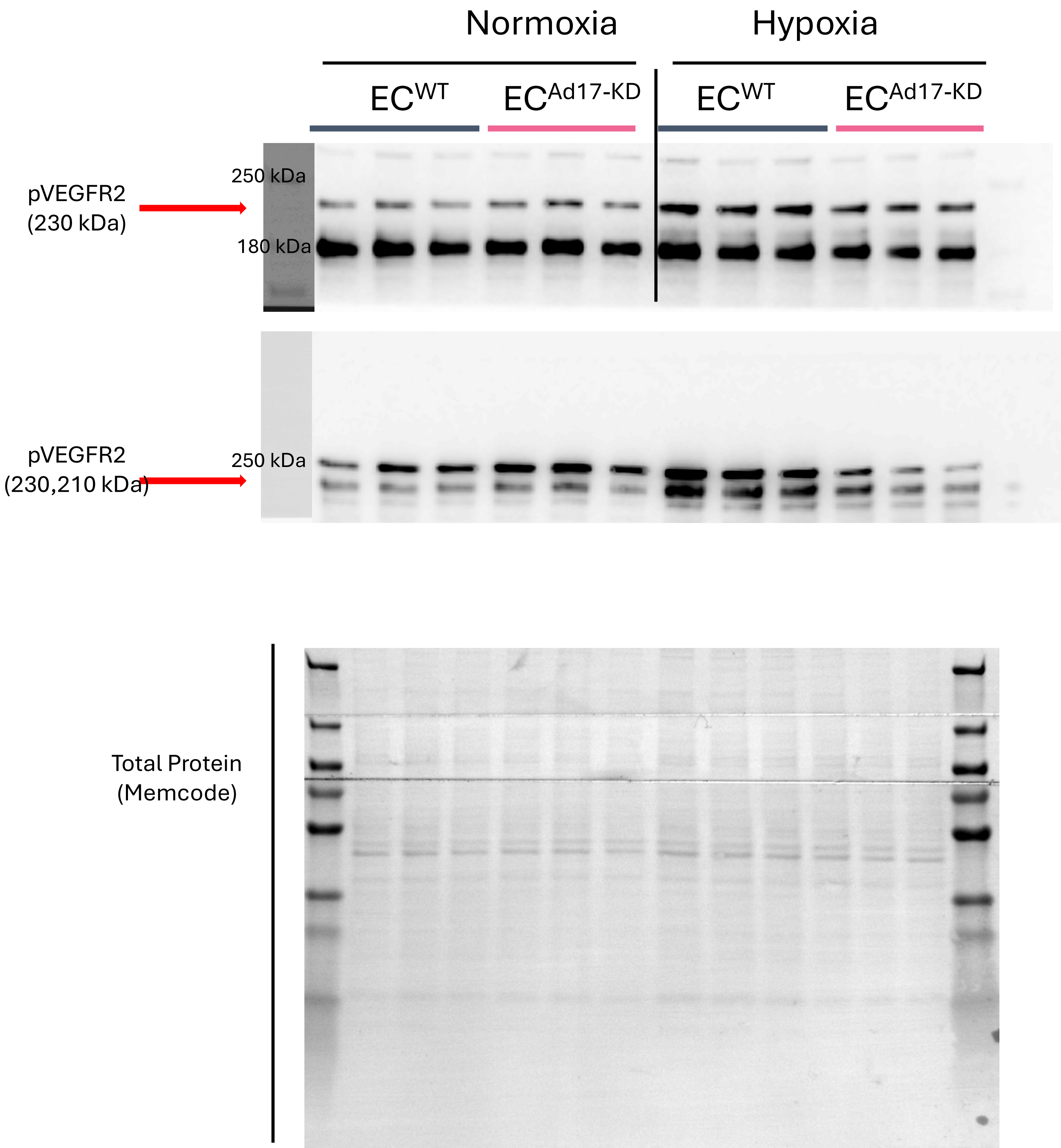

Figure 6B: Fibronectin

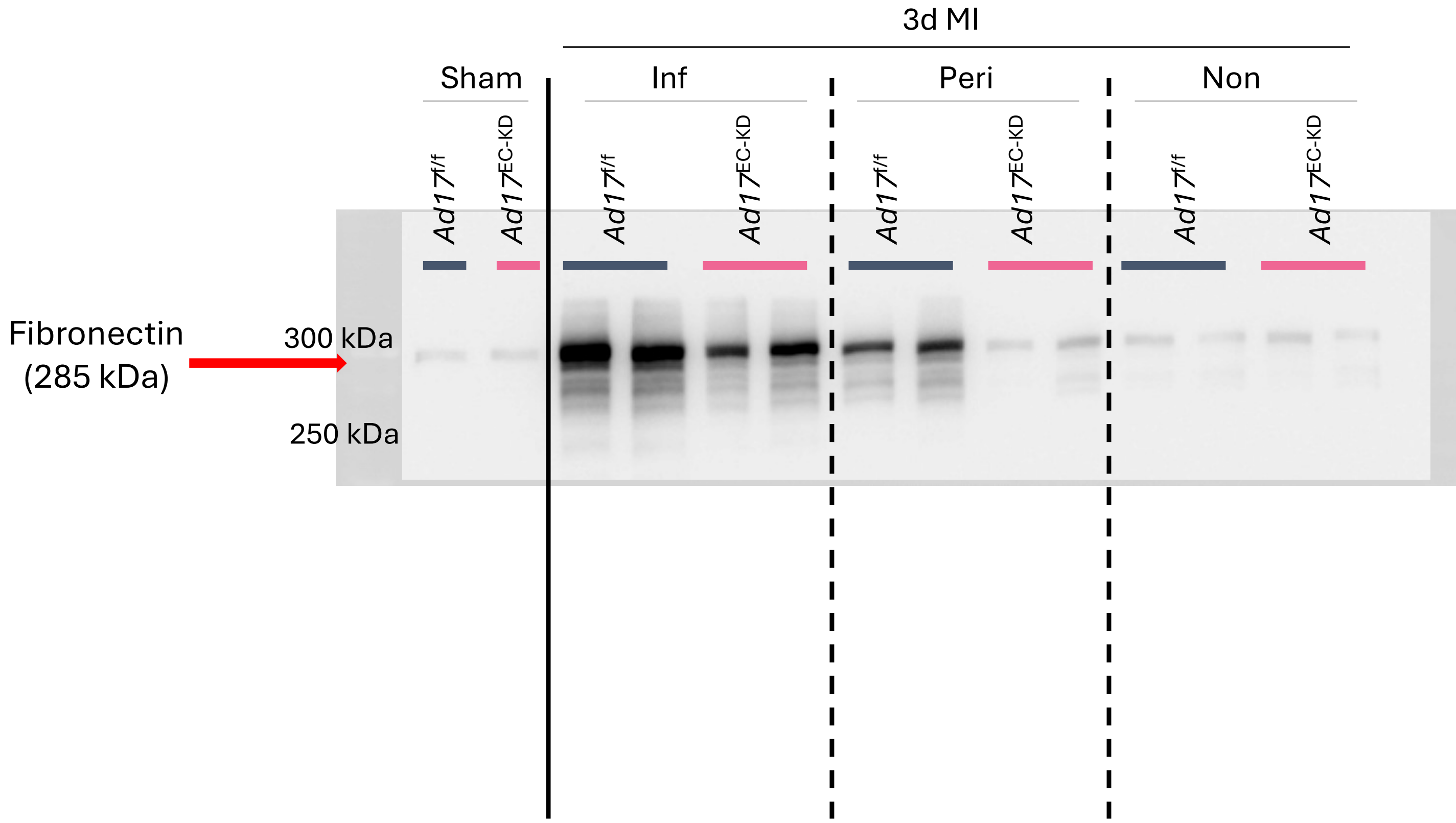

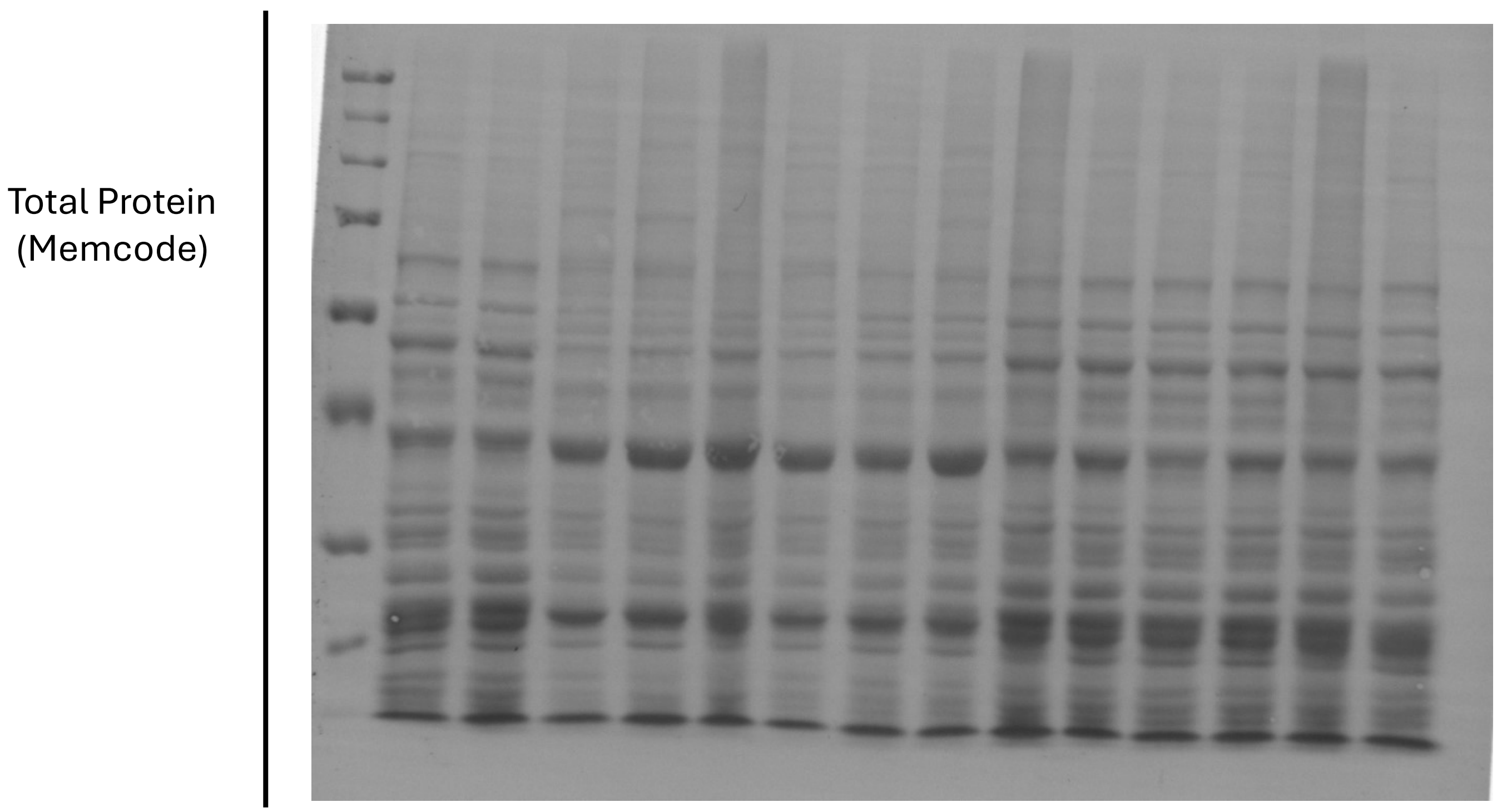

Figure 6B : pSMAD2/3 SMAD2/3

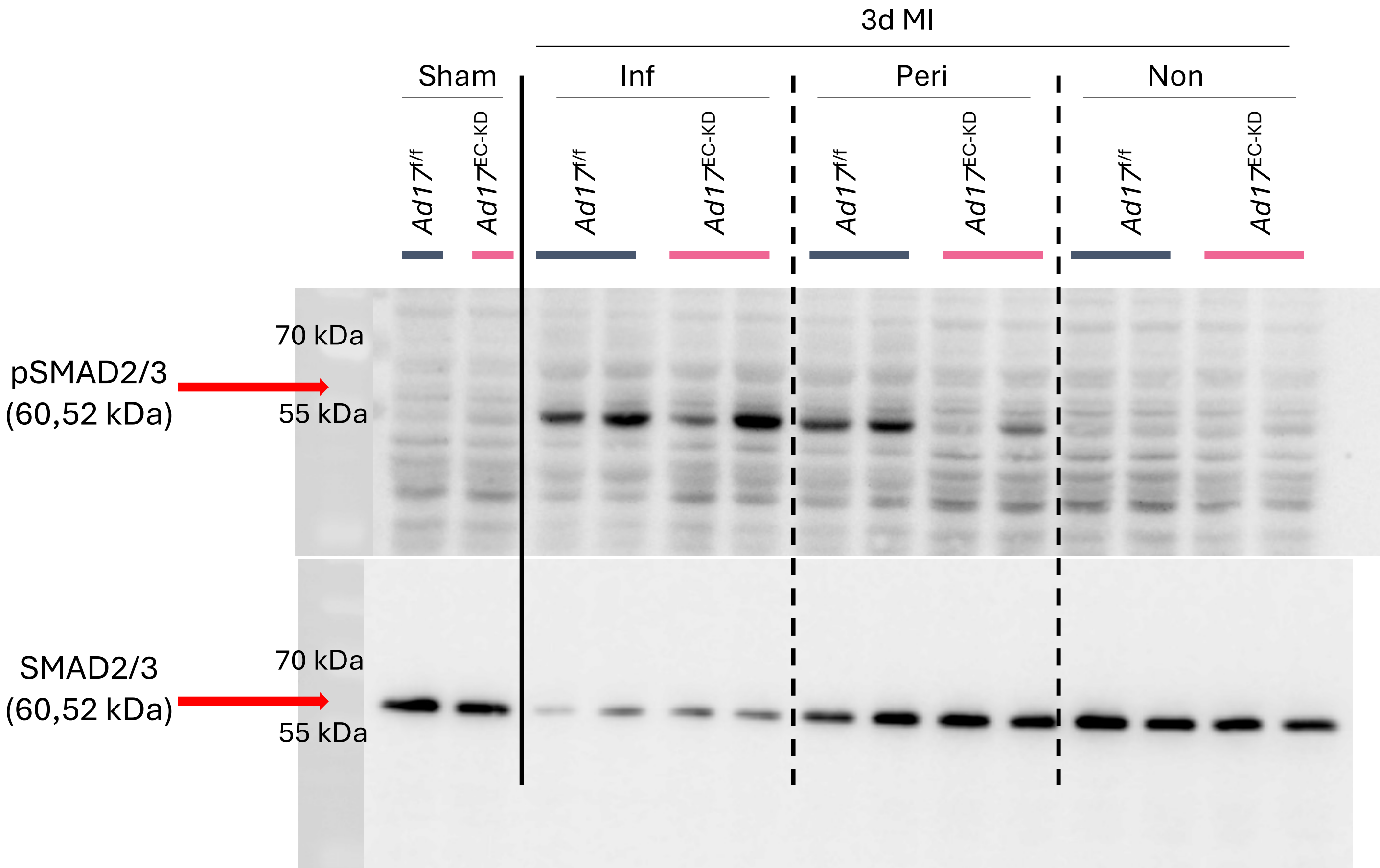

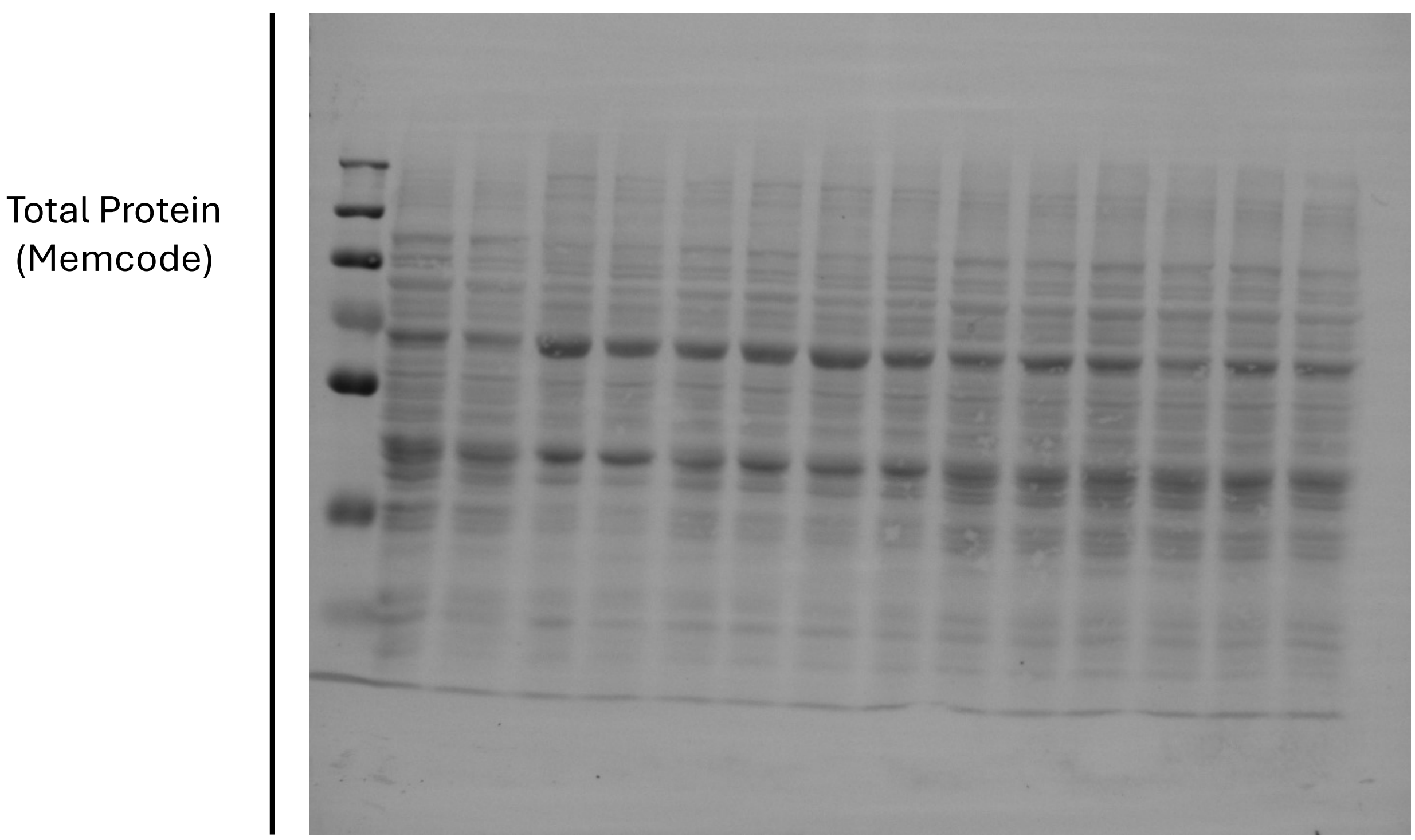

Figure 6B: Pro-LOX LOX

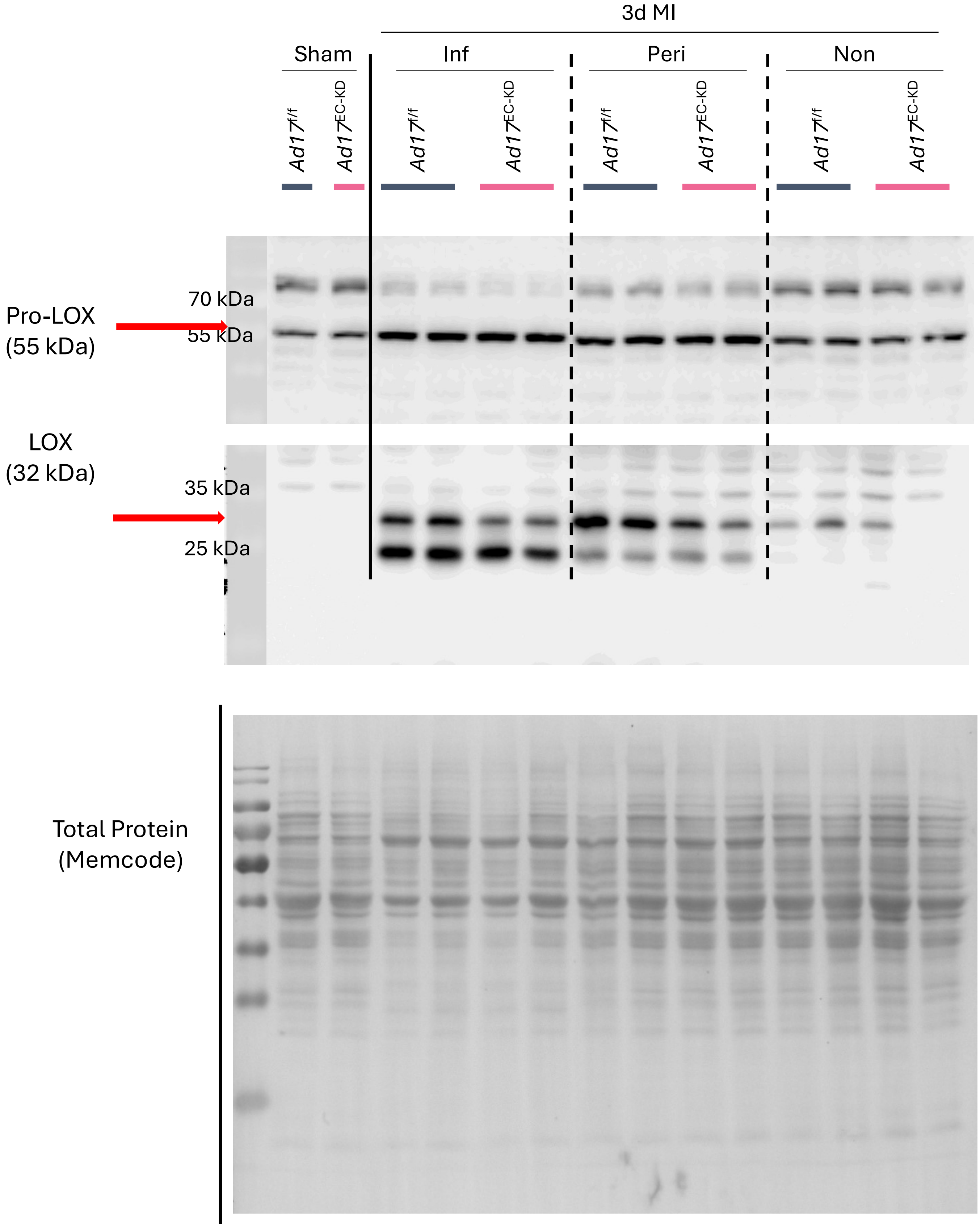

Figure 6C: pSMAD2/3 SMAD2/3

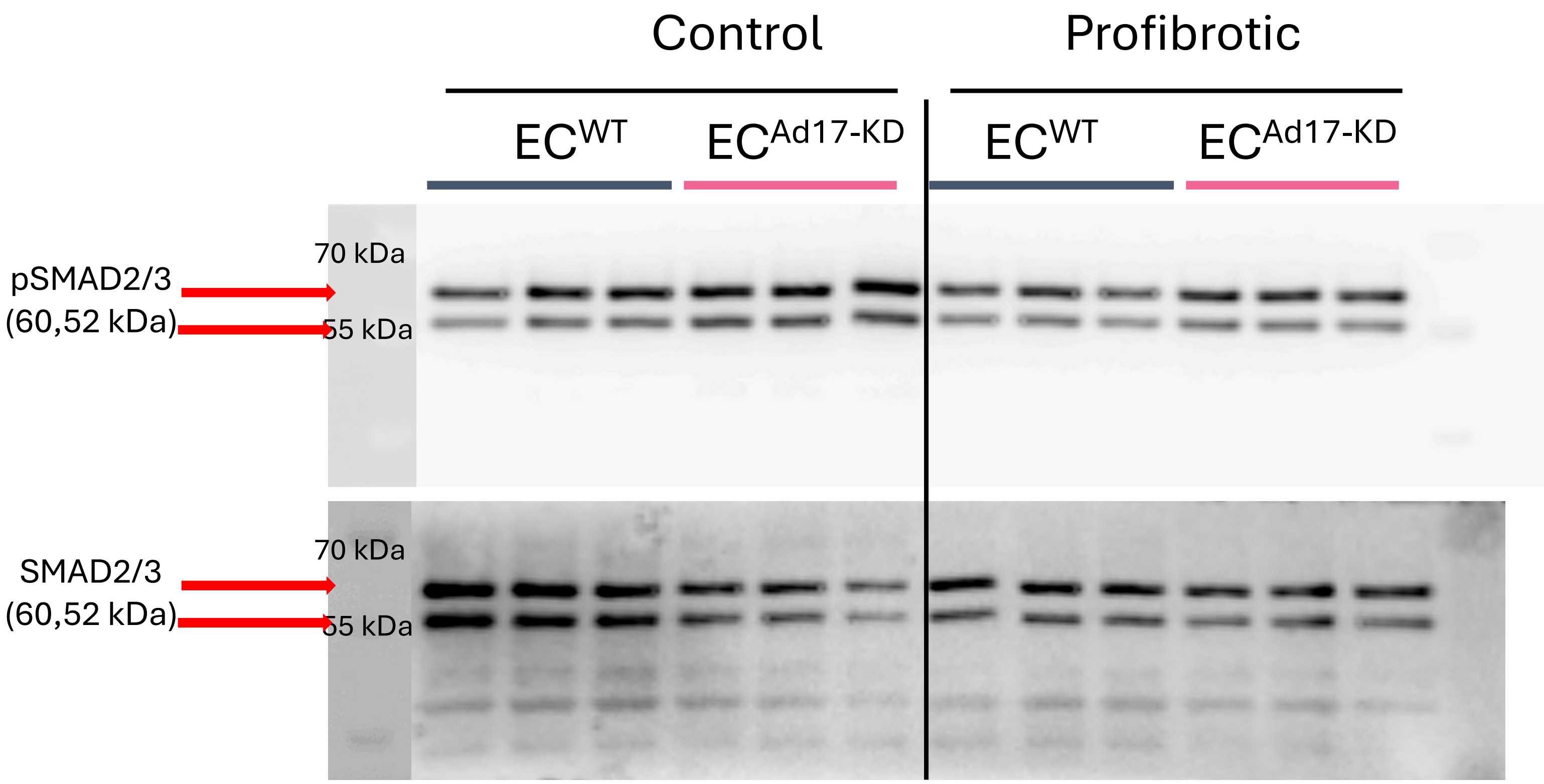

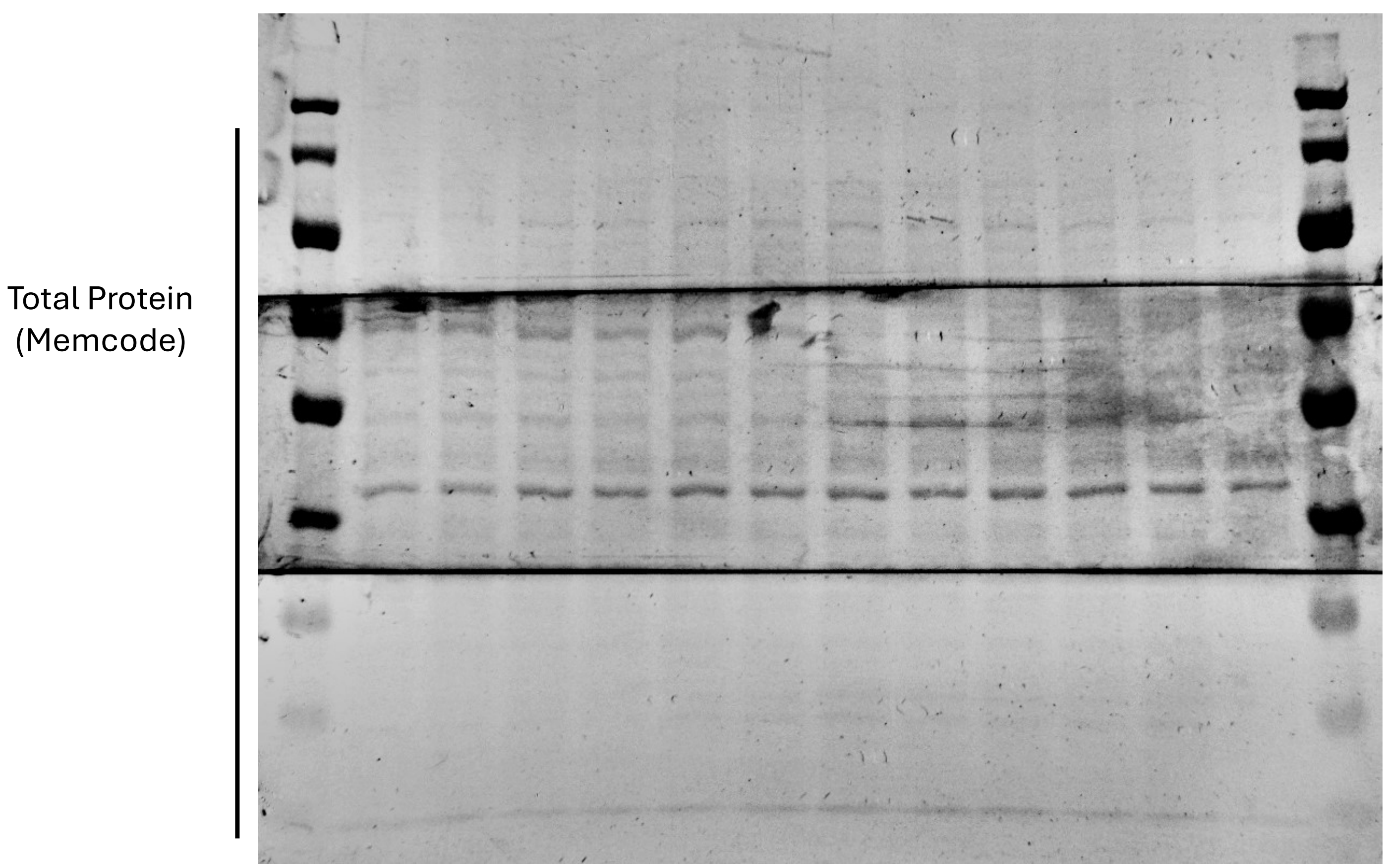

Figure 6C: SM22α

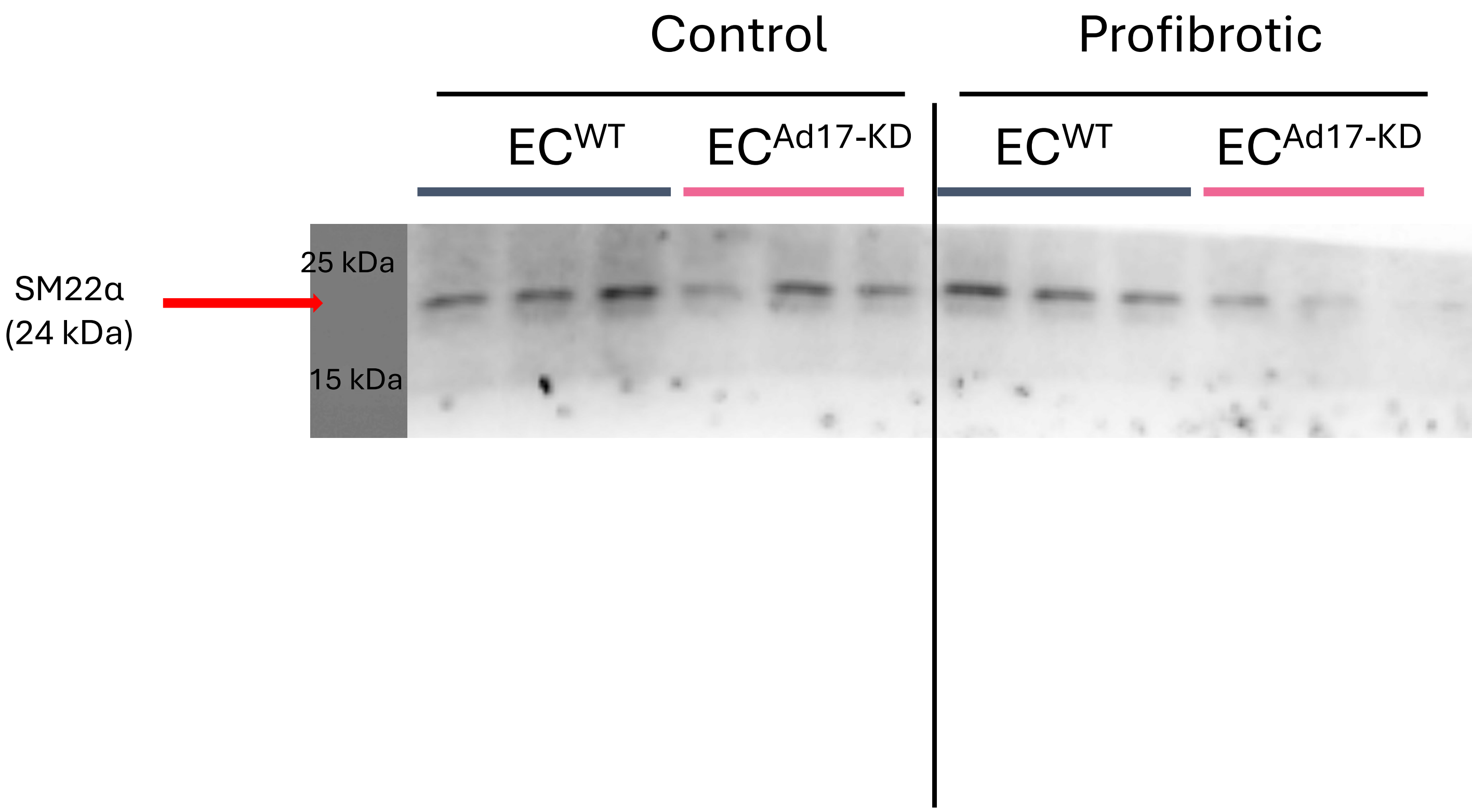

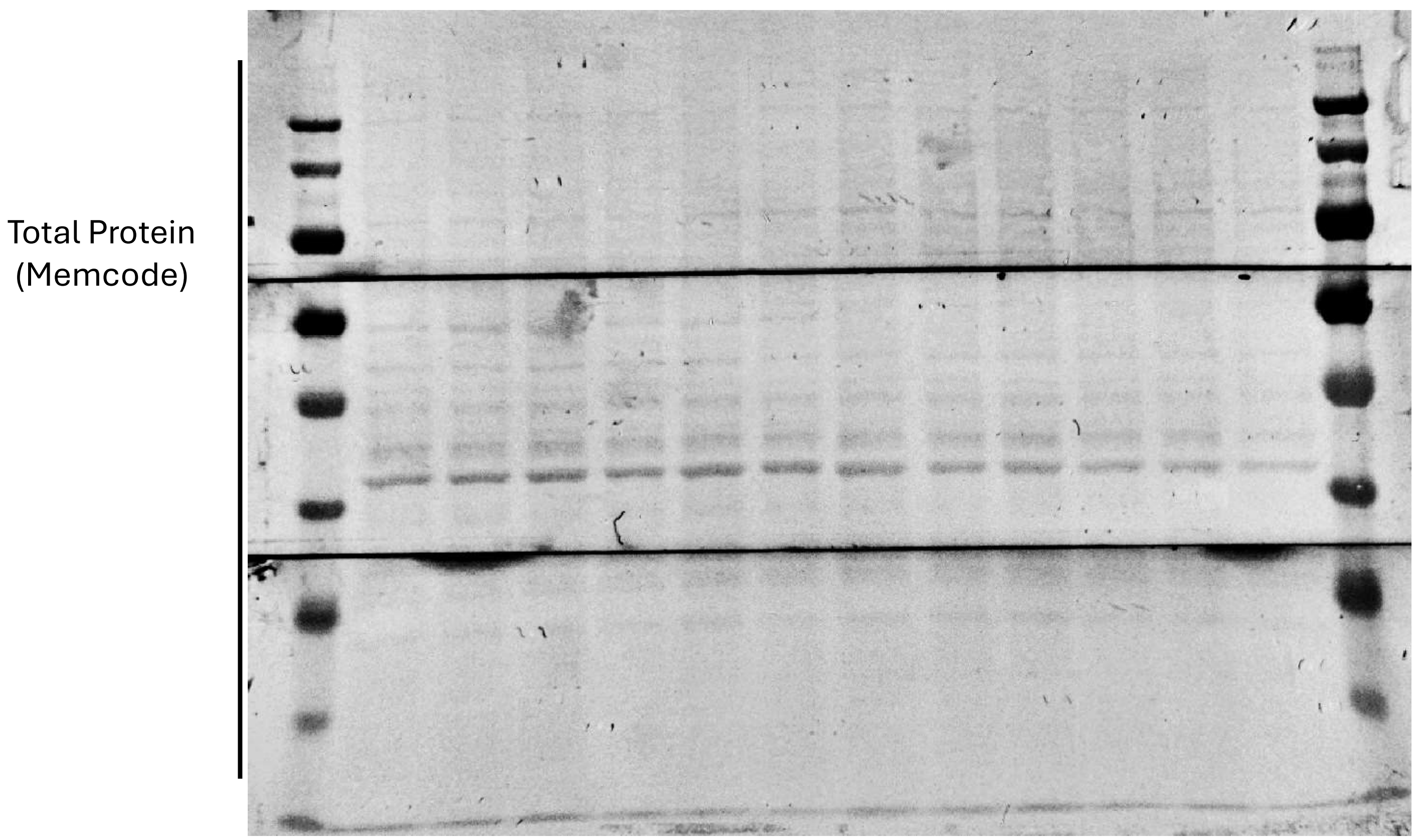

Figure 6C: αSMA

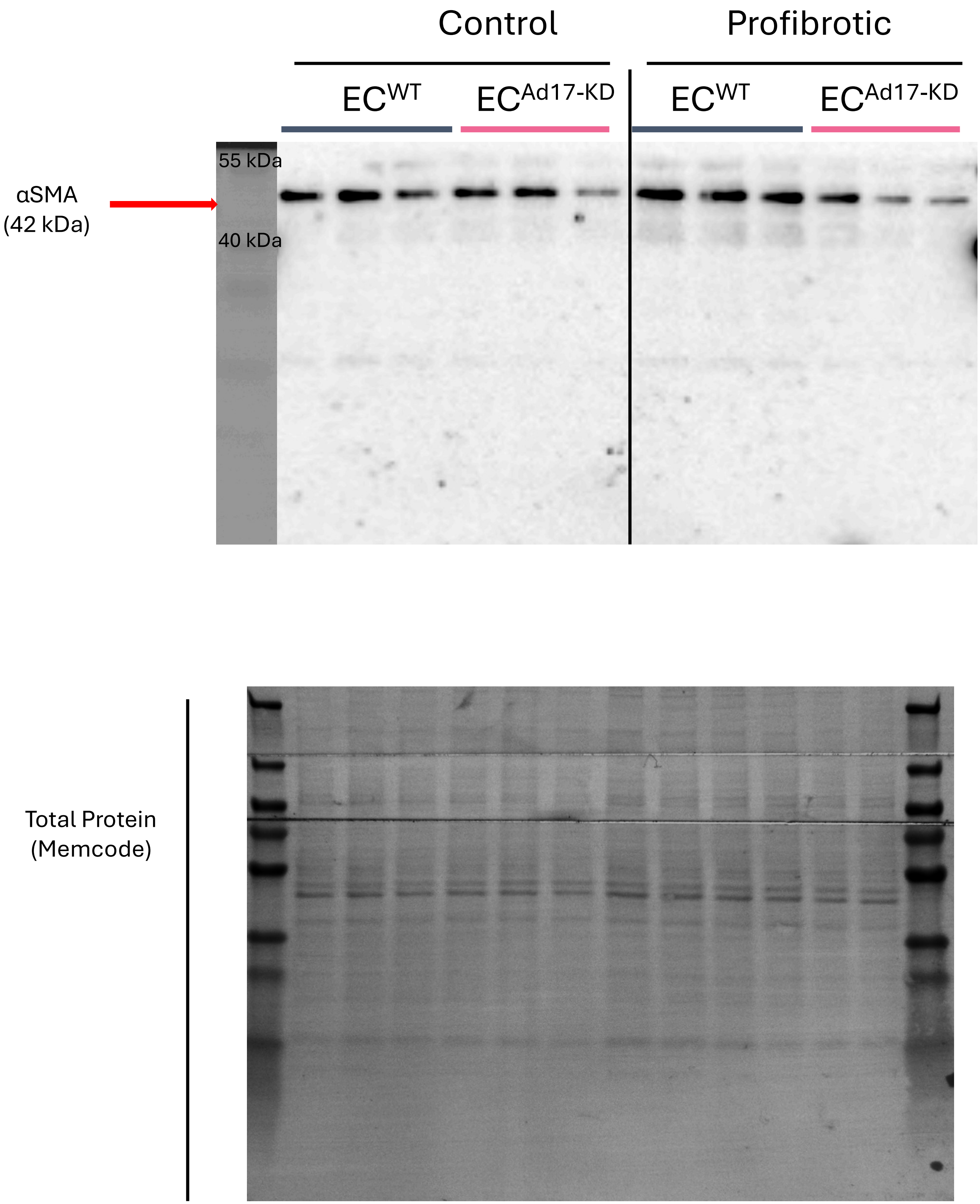

Fig 7B: Pro-Caspase 3 + caspase 3

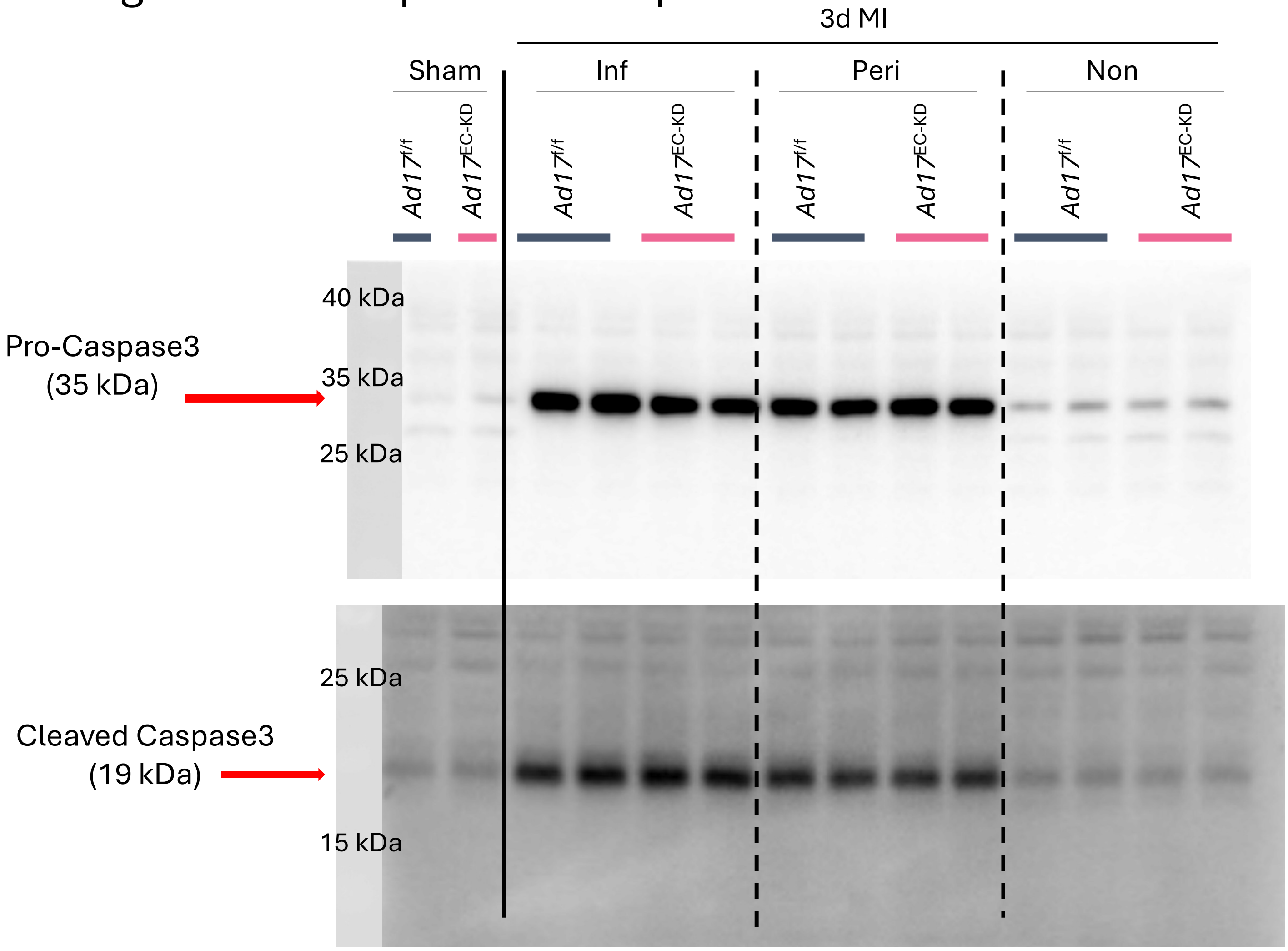

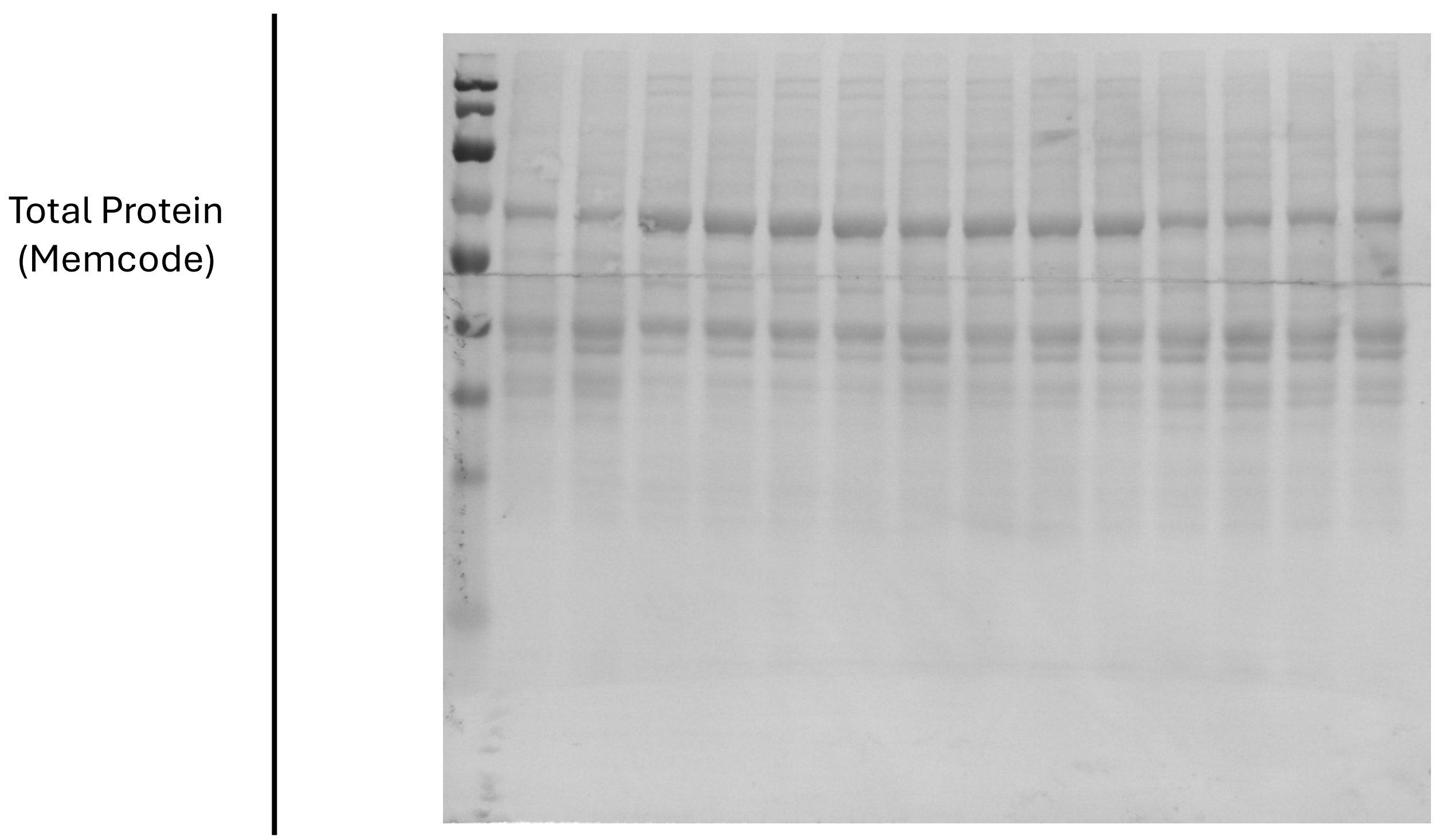

Fig 7B: Pro-Caspase 8 + caspase 8

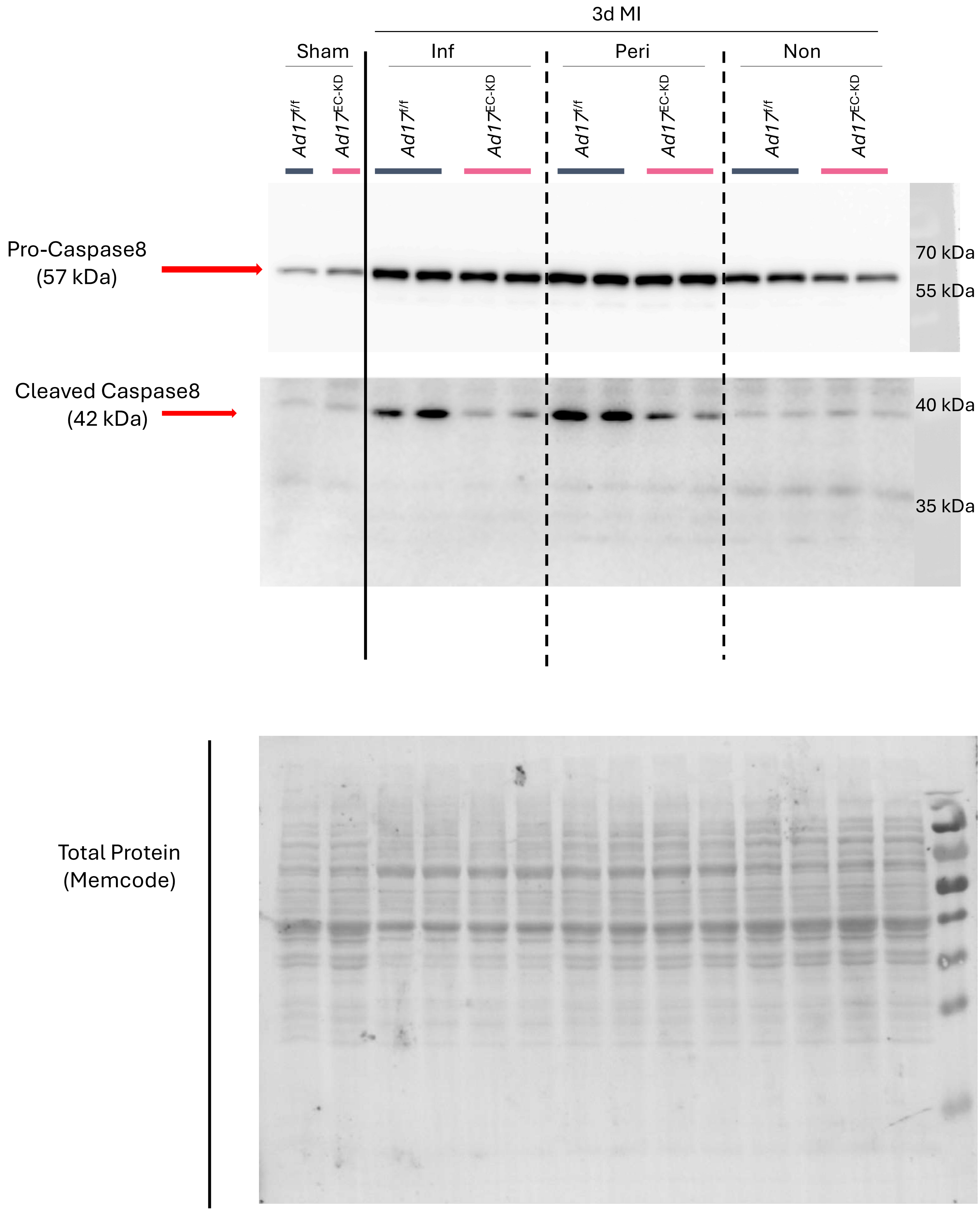

Fig 7C: pMLKL + MLKL

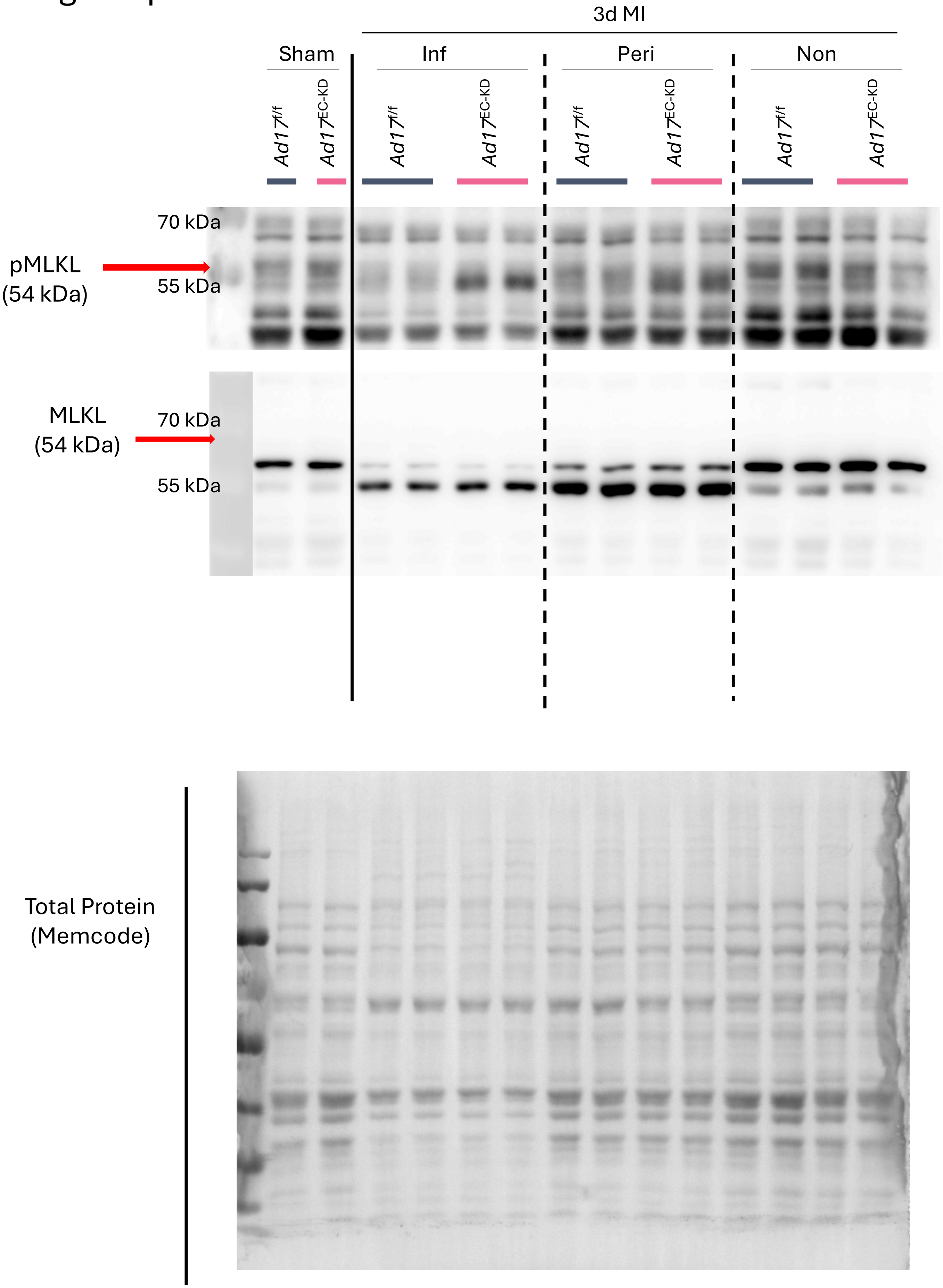

Fig 7C: RIPK3

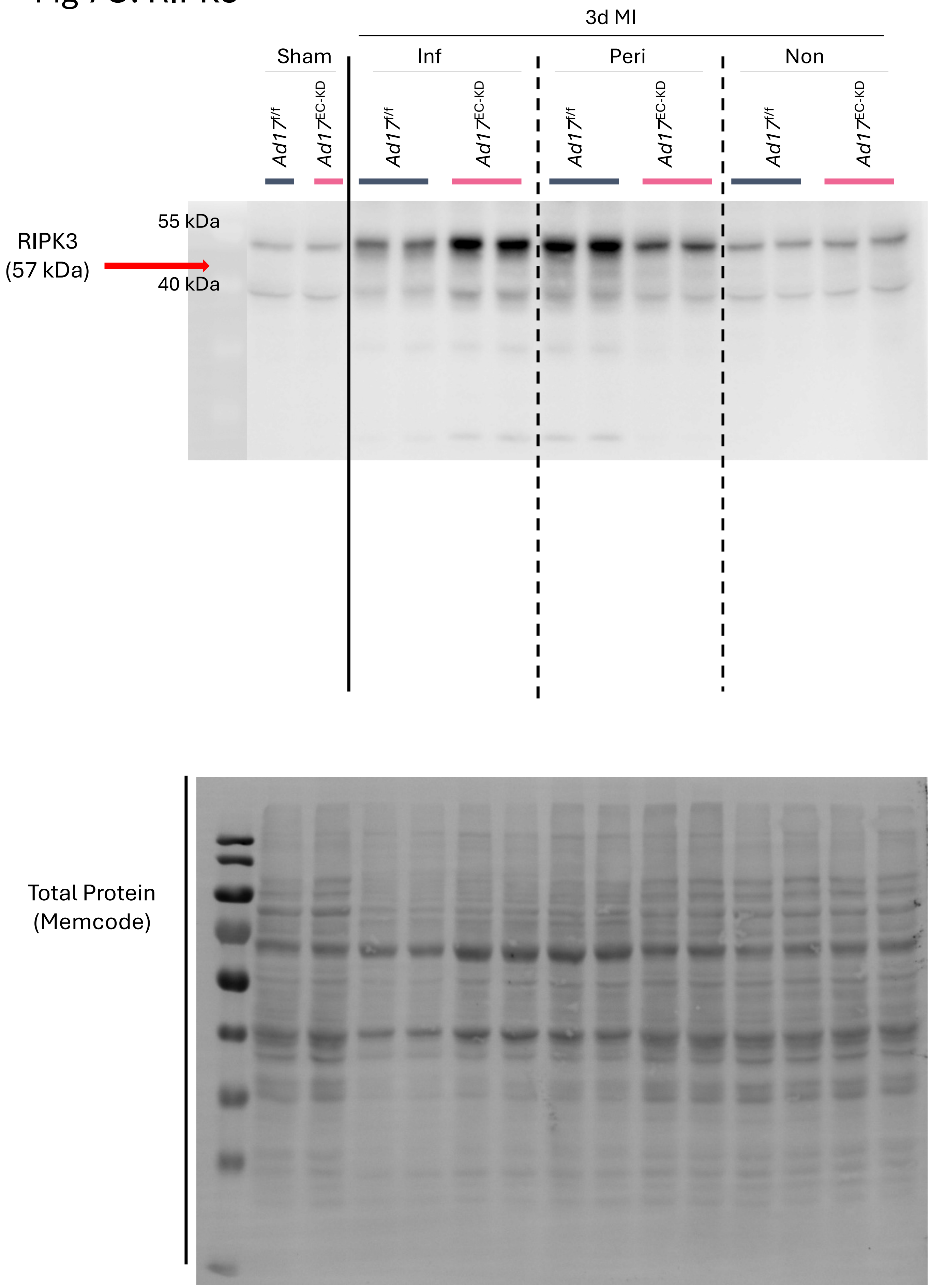

3d MI
Sham
Inf
Peri
Non
Ad17f/f
Ad17EC-KD
Ad17f/f
Ad17EC-KD
Ad17f/f
Ad17EC-KD
Ad17f/f
Ad17EC-KD
RIPK3
(57 kDa)
55 kDa
40 kDa
Total Protein
(Memcode)

Fig 7C: RIPK1

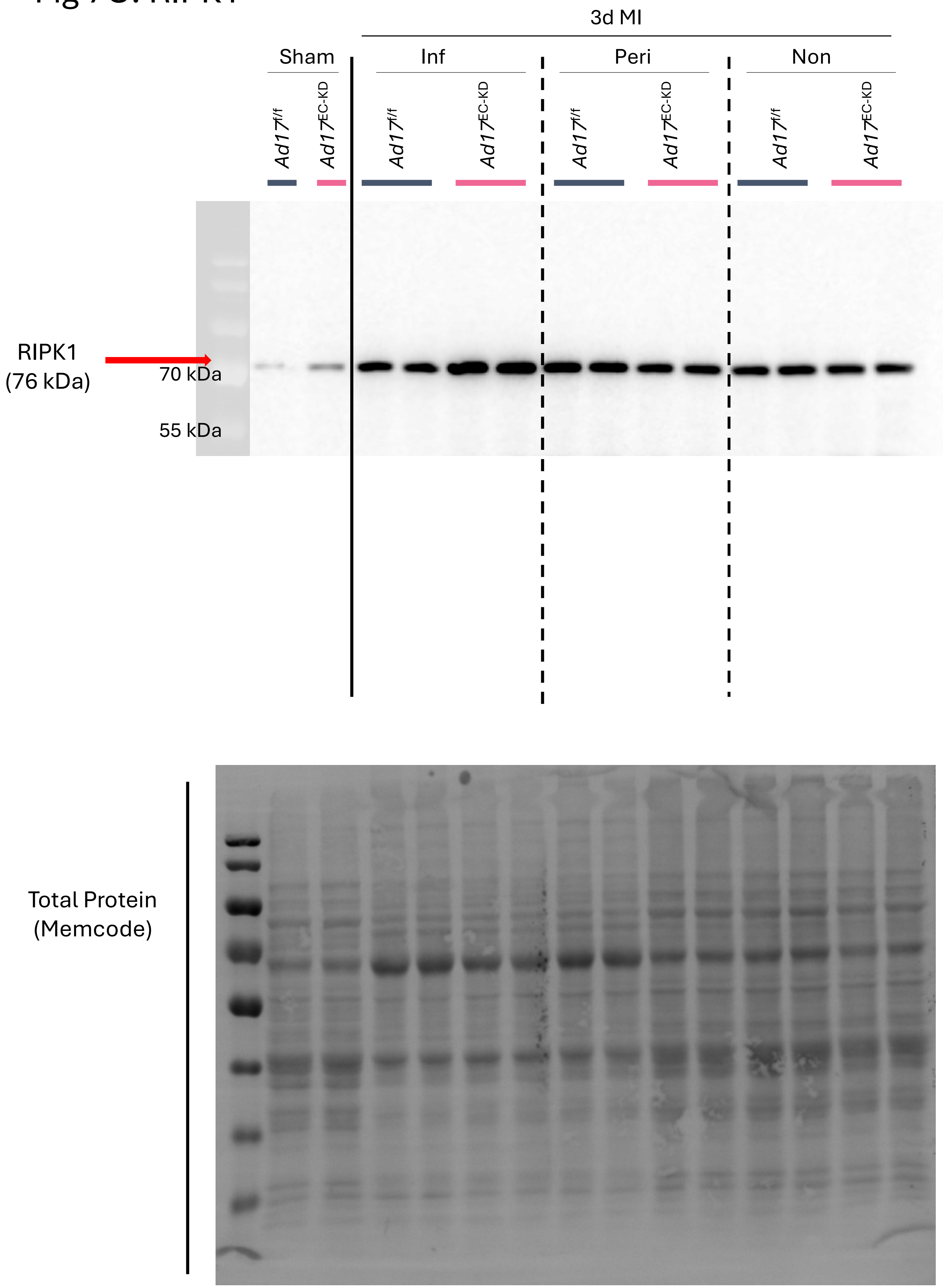

3d MI
Sham
Inf
Peri
Non
Ad17f/f
Ad17EC-KD
Ad17f/f
Ad17EC-KD
Ad17f/f
Ad17EC-KD
Ad17f/f
Ad17EC-KD
RIPK1
(76 kDa)
70 kDa
55 kDa
Total Protein
(Memcode)

Fig 7C: TNFR1

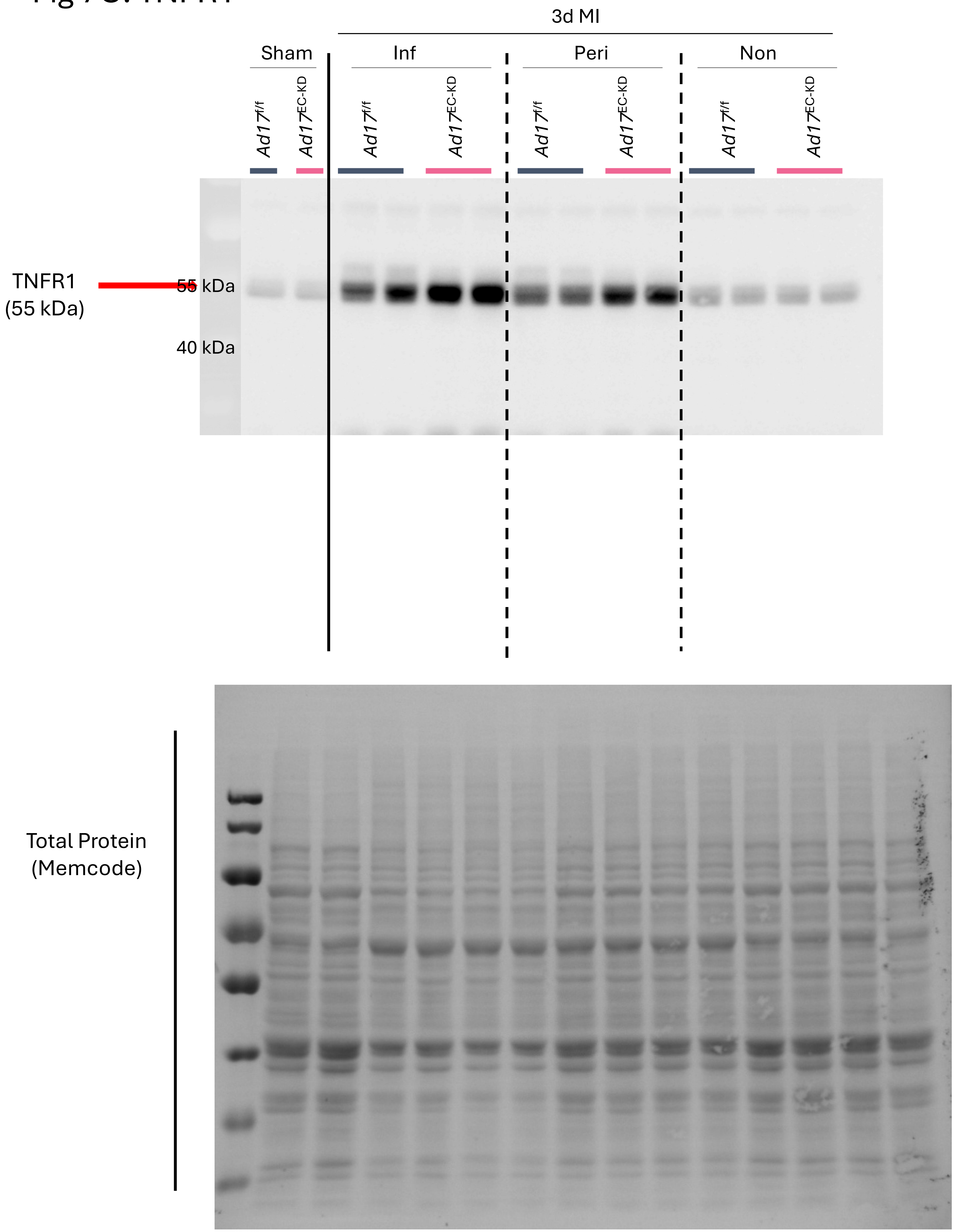
3d MI
Sham
Inf
Peri
Non
Ad17f/f
Ad17EC-KD
Ad17f/f
Ad17EC-KD
Ad17f/f
Ad17EC-KD
Ad17f/f
Ad17EC-KD
TNFR1
(55 kDa)
55 kDa
40 kDa
Total Protein
(Memcode)

Fig 7C: TNFR2

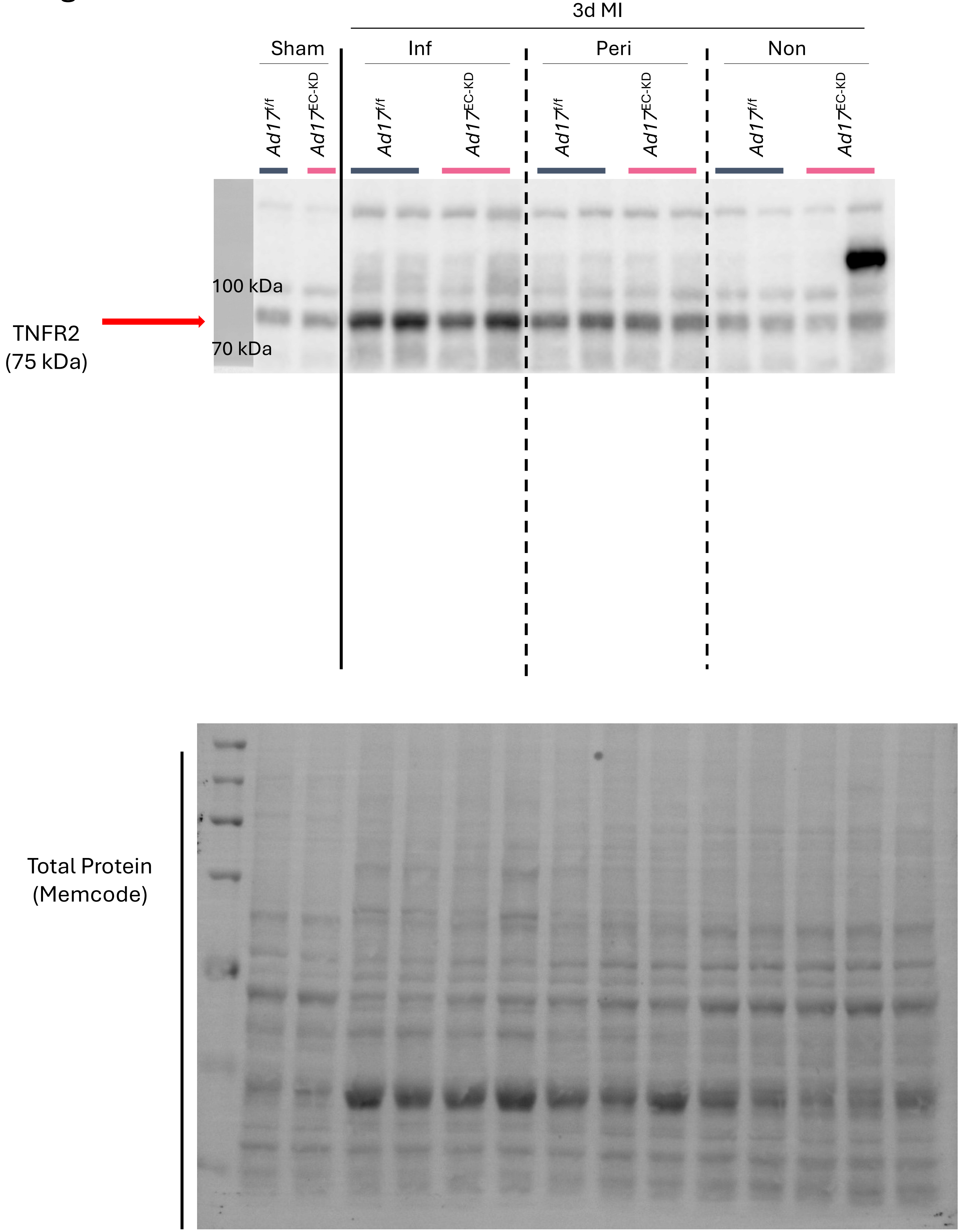
3d MI
Sham
Inf
Peri
Non
Ad17f/f
Ad17EC-KD
Ad17f/f
Ad17EC-KD
Ad17f/f
Ad17EC-KD
Ad17f/f
Ad17EC-KD
100 kDa
70 kDa
TNFR2
(75 kDa)
Total Protein
(Memcode)

Fig 7C: TNFα

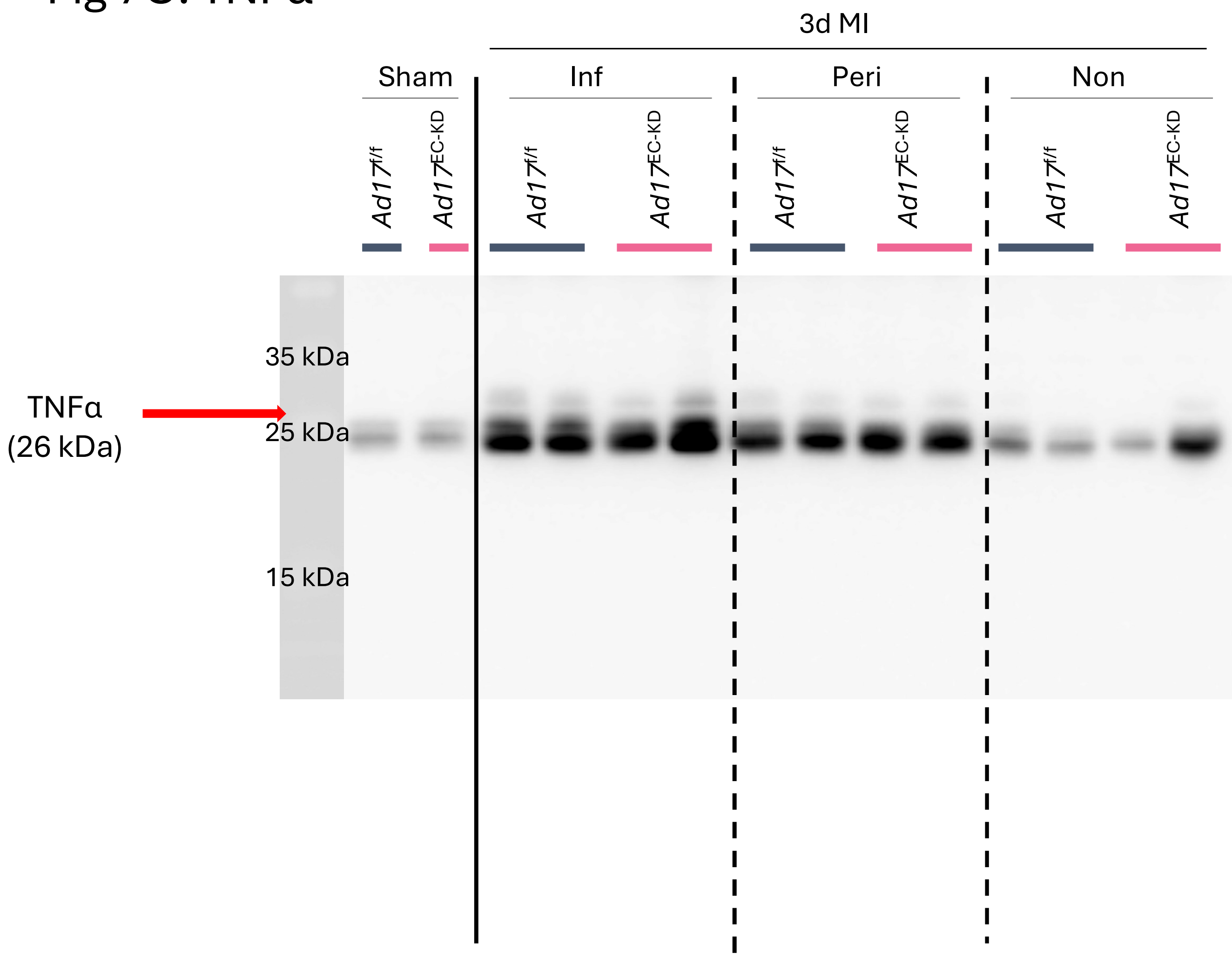
3d MI
Sham
Inf
Peri
Non
Ad17f/f
Ad17EC-KD
Ad17f/f
Ad17EC-KD
Ad17f/f
Ad17EC-KD
Ad17f/f
Ad17EC-KD
TNFα
(26 kDa)
35 kDa
25 kDa
15 kDa

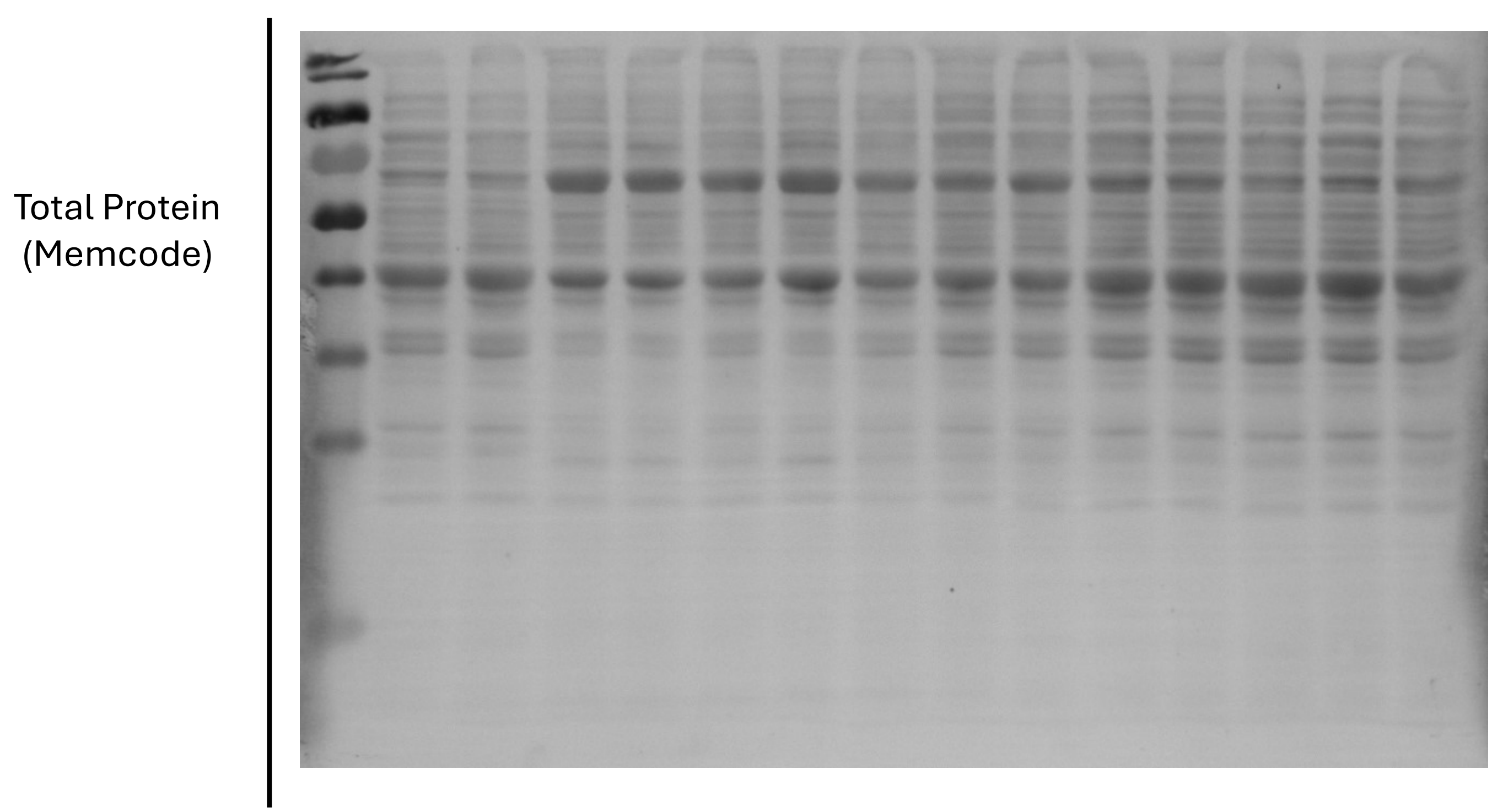
Total Protein
(Memcode)

Fig 8B: pMLKL & MLKL

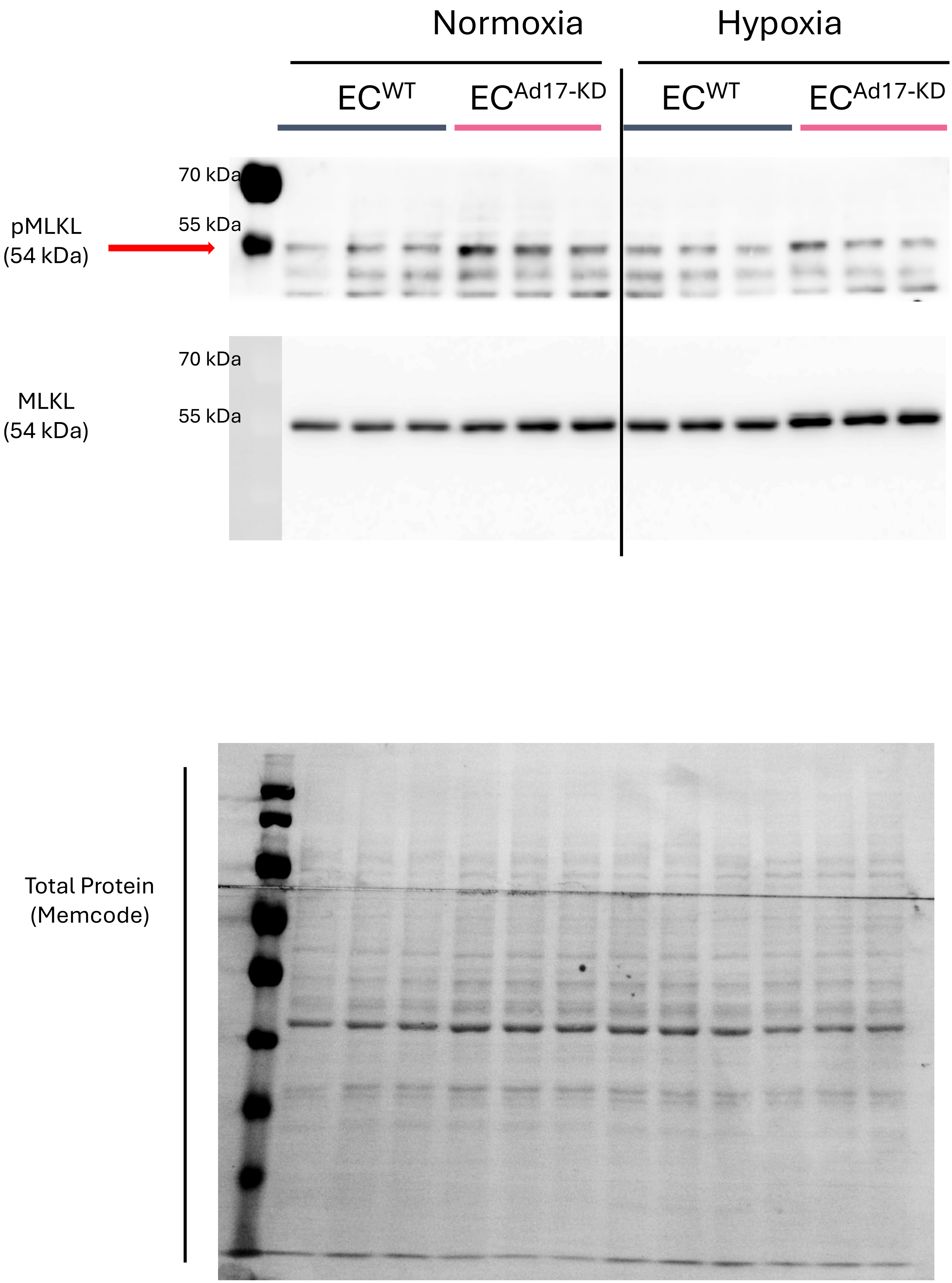

Fig 8B: TNFR1

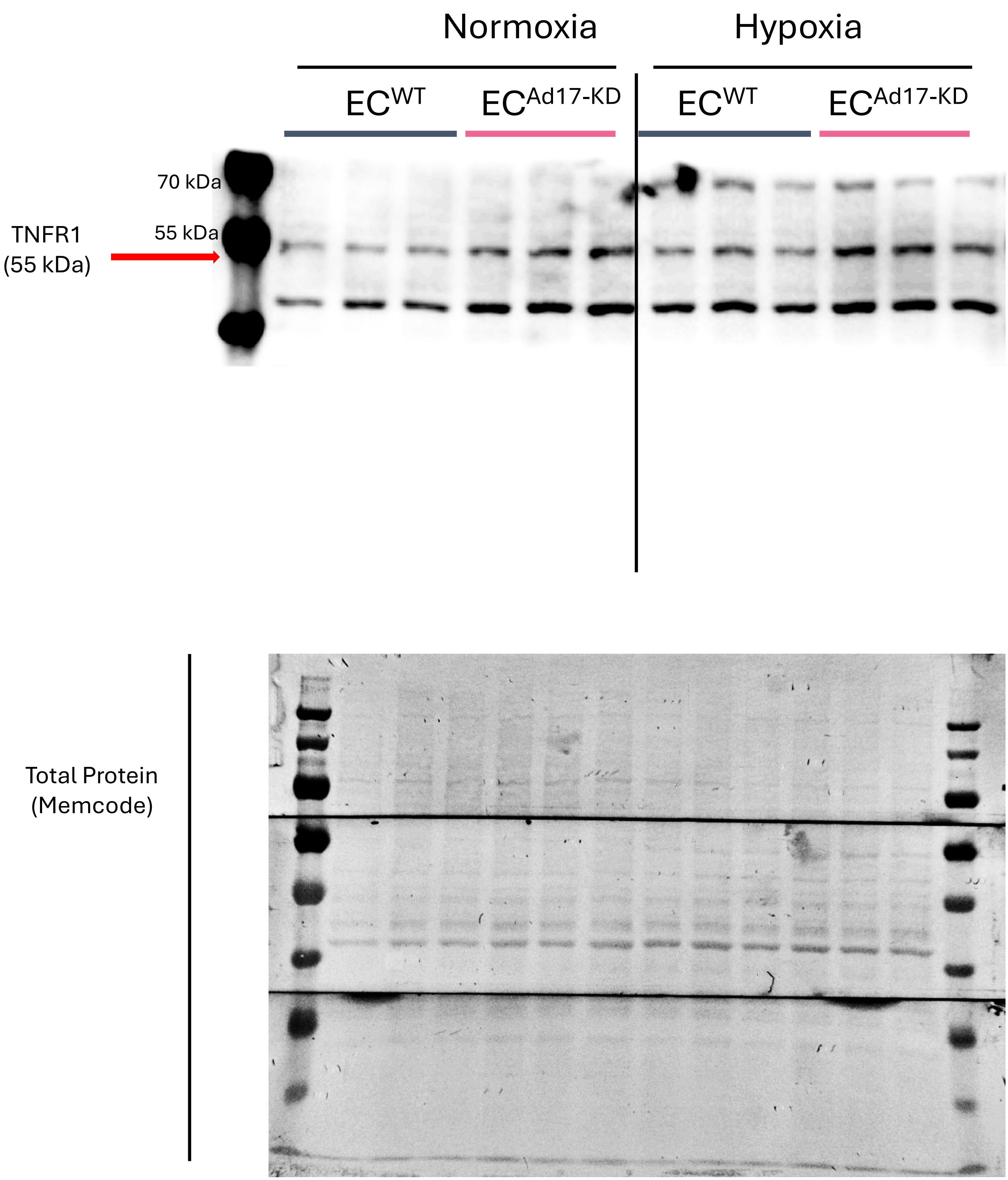

Normoxia
Hypoxia
EC^WT
EC^Ad17-KD
EC^WT
EC^Ad17-KD
70 kDa
55 kDa
TNFR1
(55 kDa)
Total Protein
(Memcode)

Fig 8B: sTNFR1

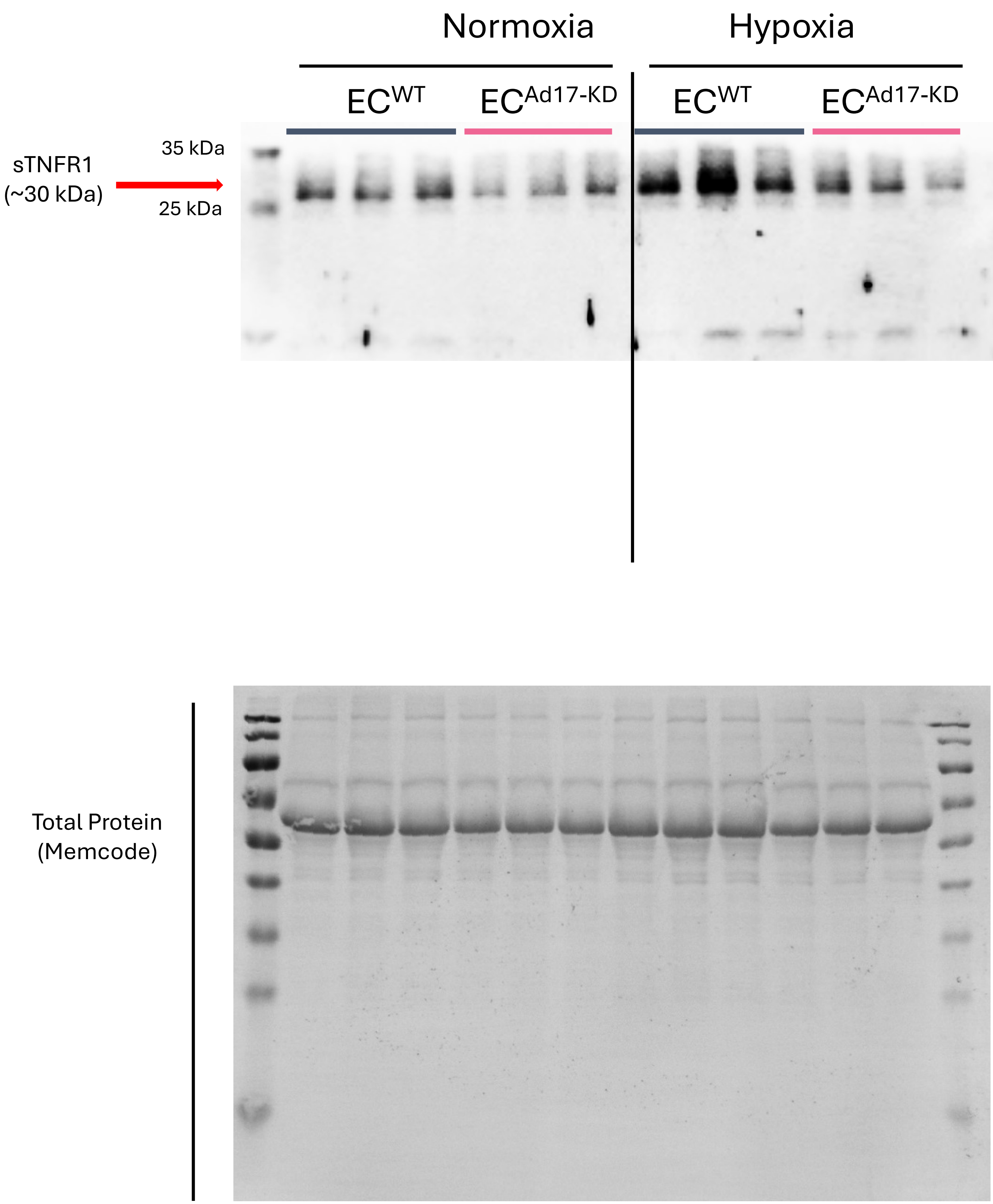

Fig_S2C: ADAM17

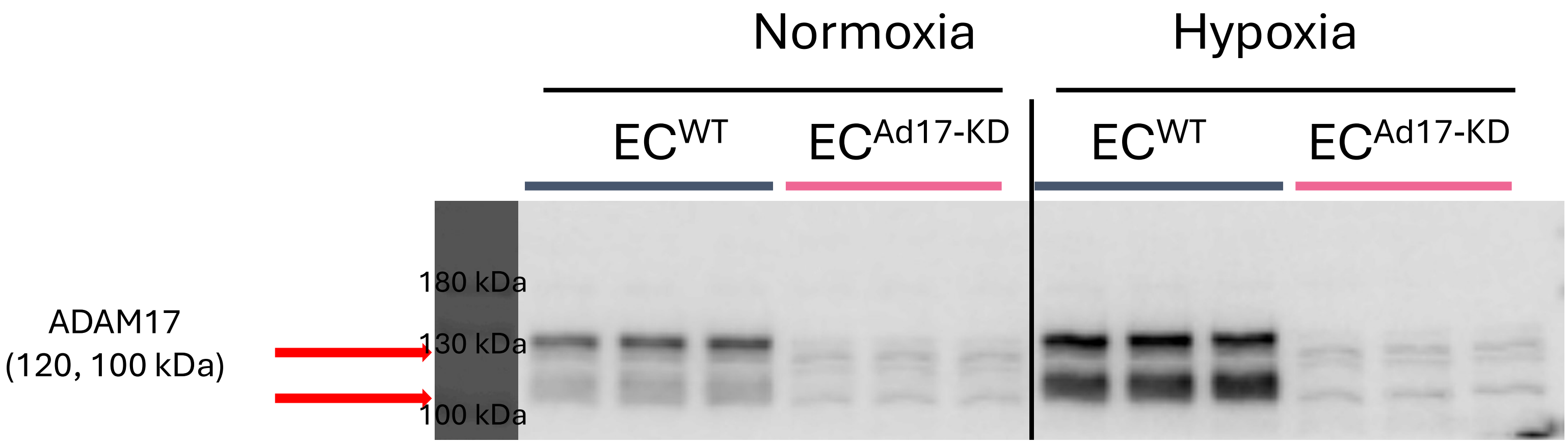

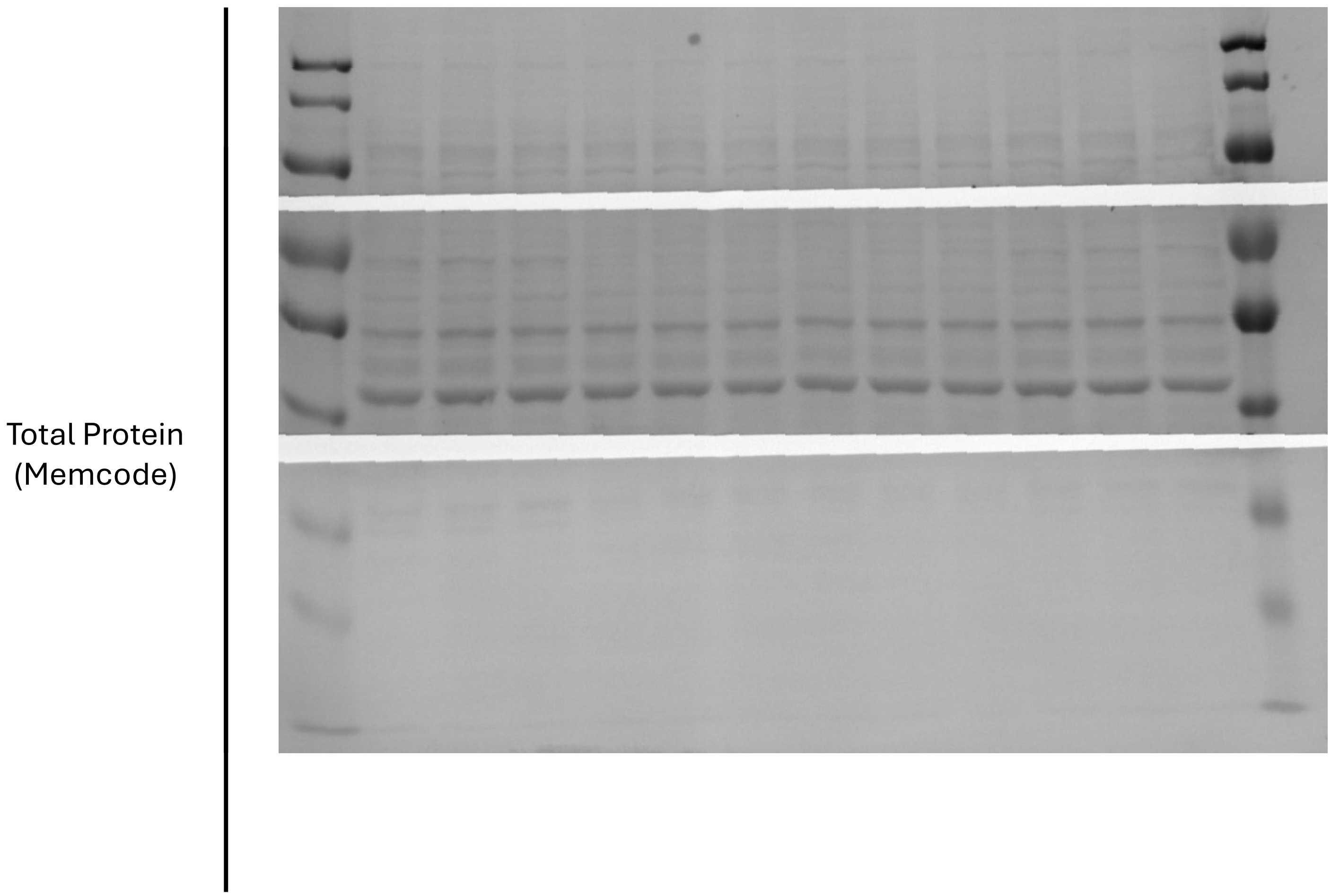

Fig_S6B: NOTCH1 NEXT NICD

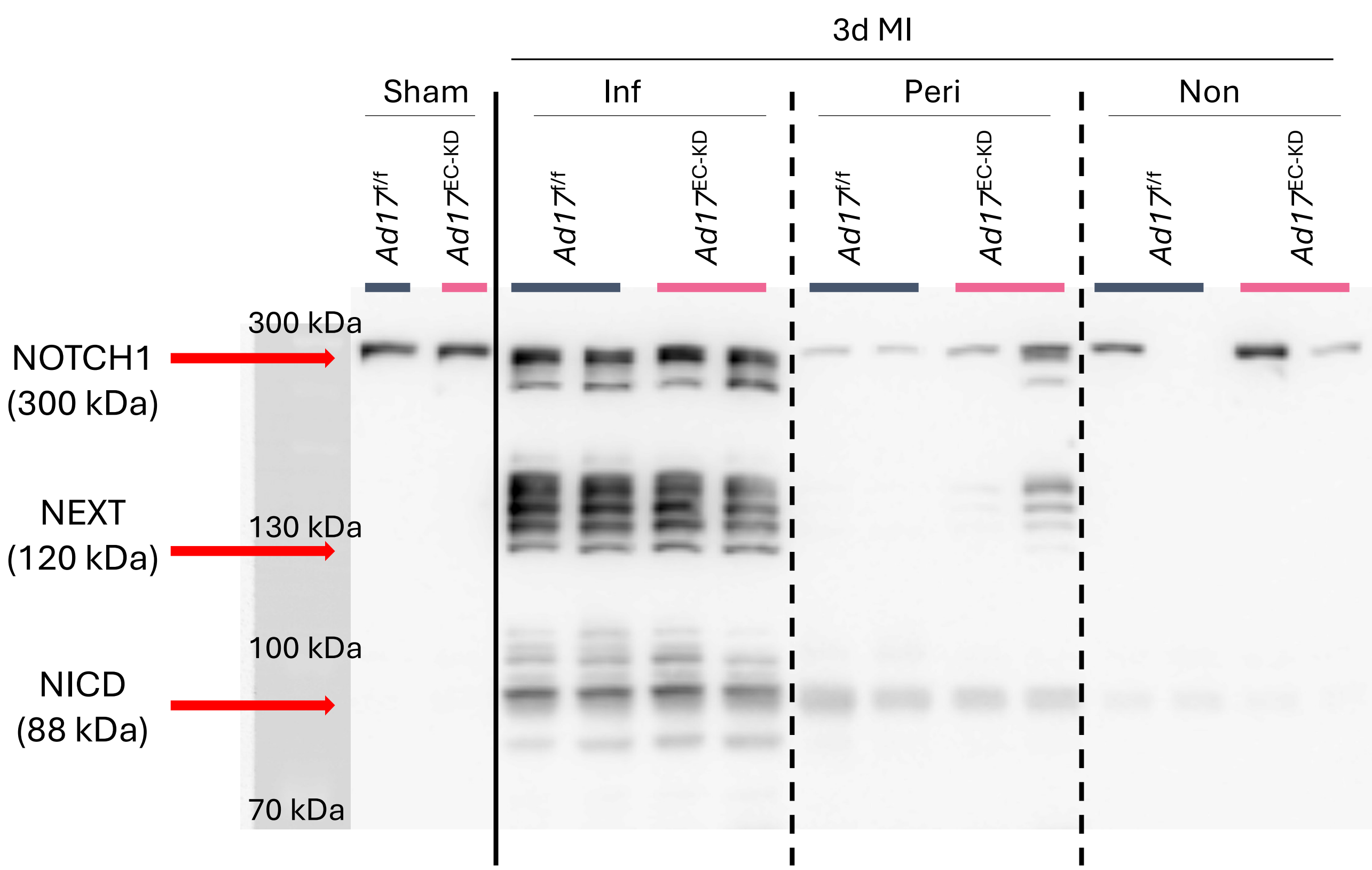

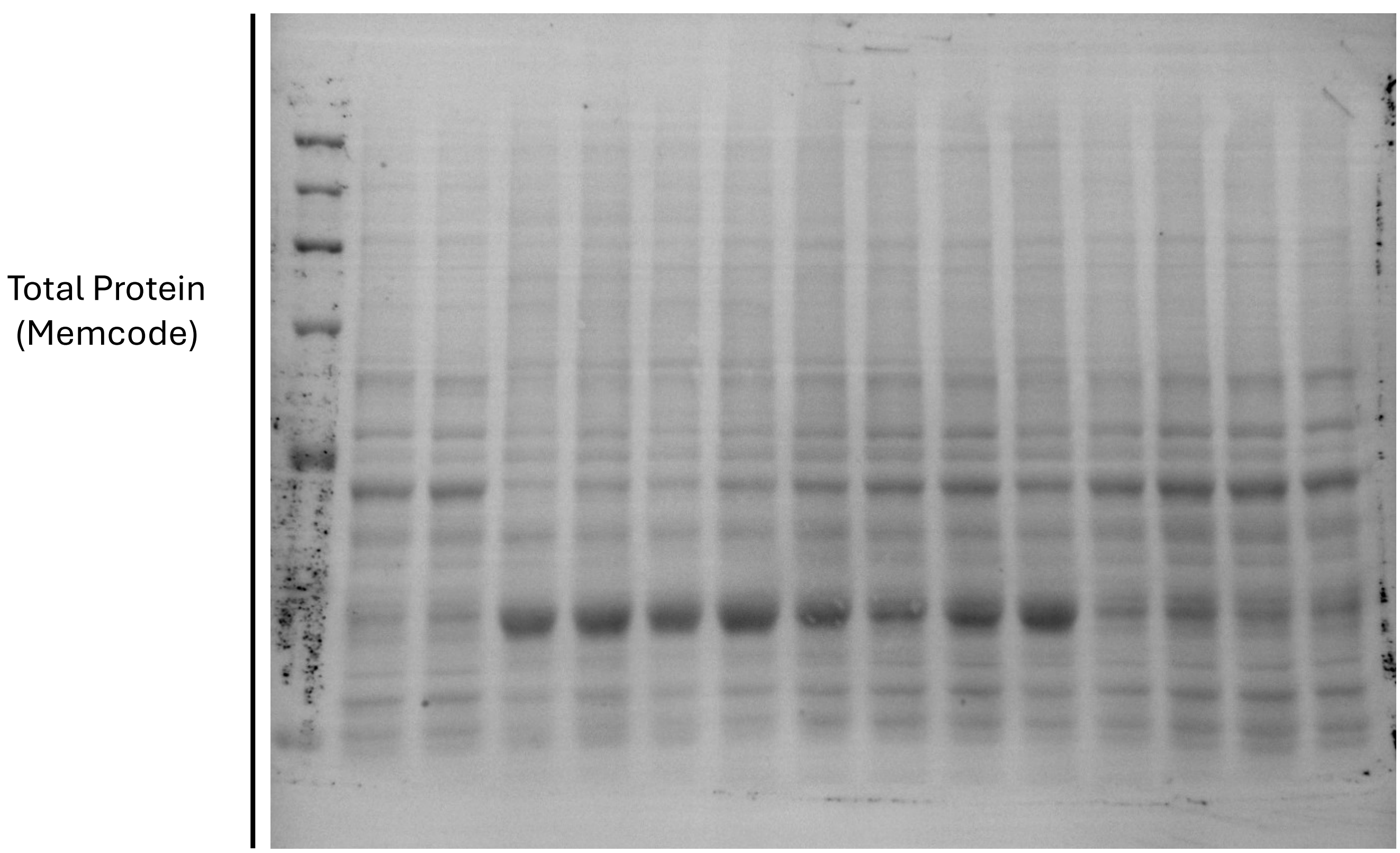

Fig_S6C: pVEGFR2 VEGFR2

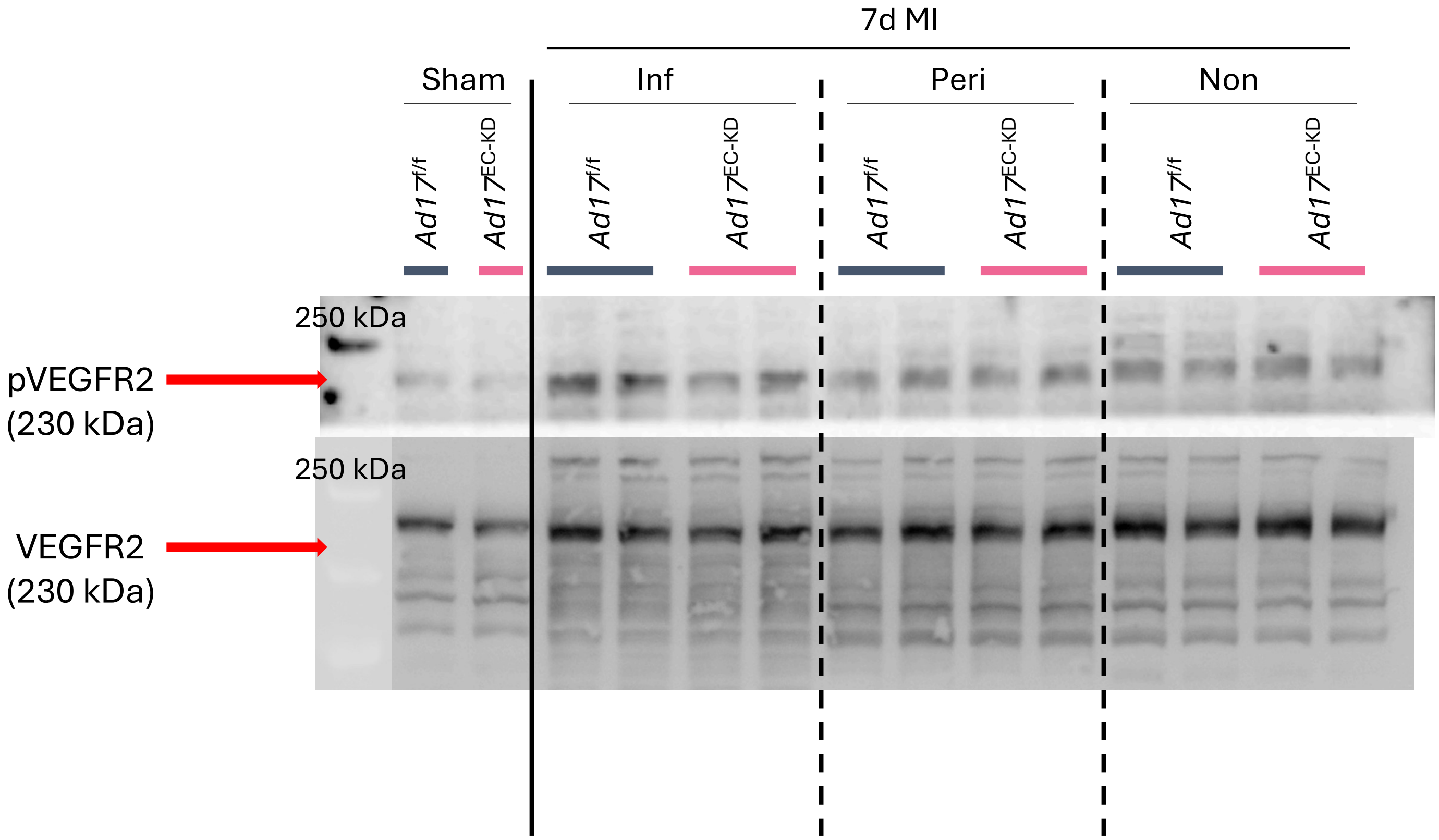

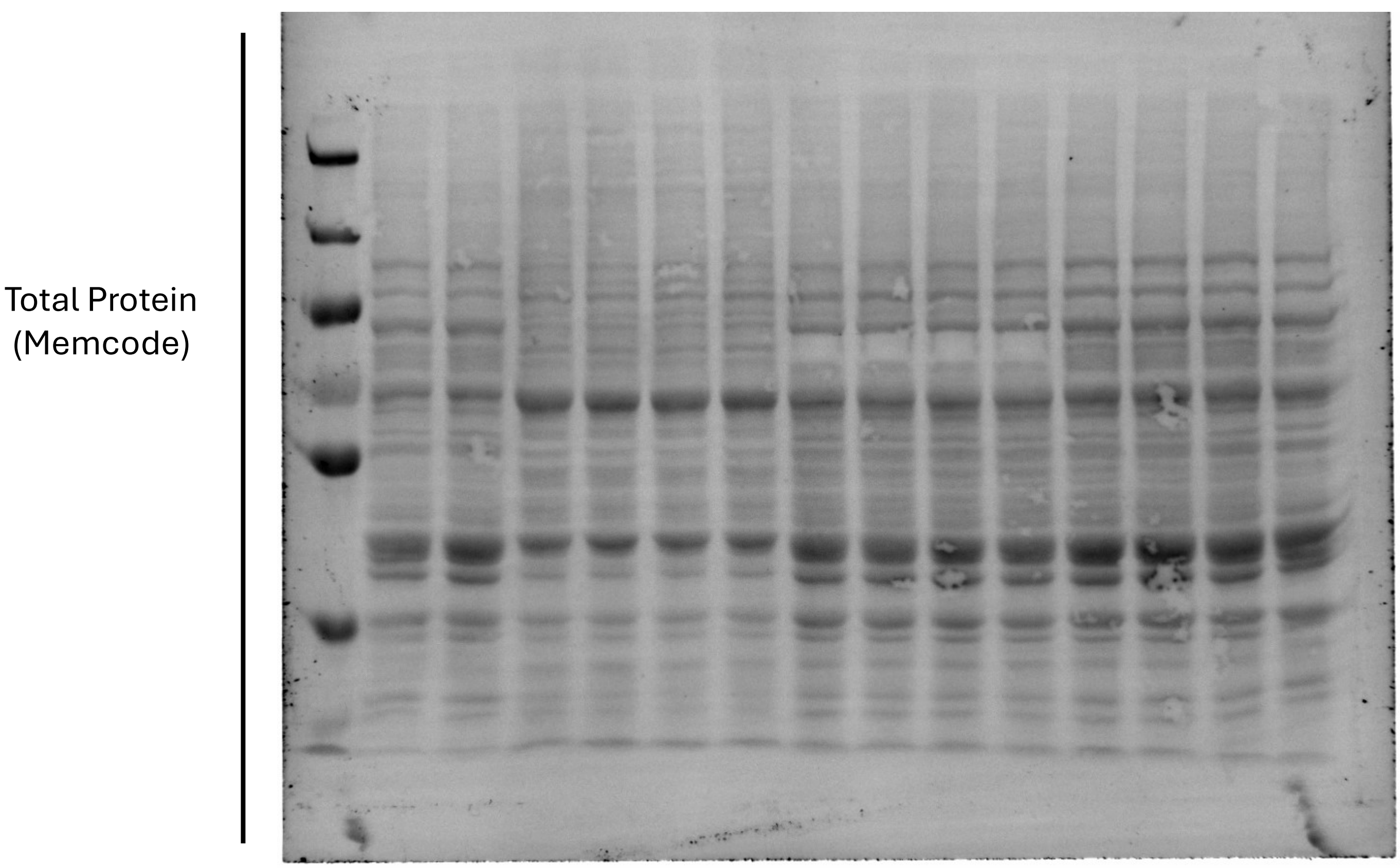

Fig_S6C: NOTCH1 NICD

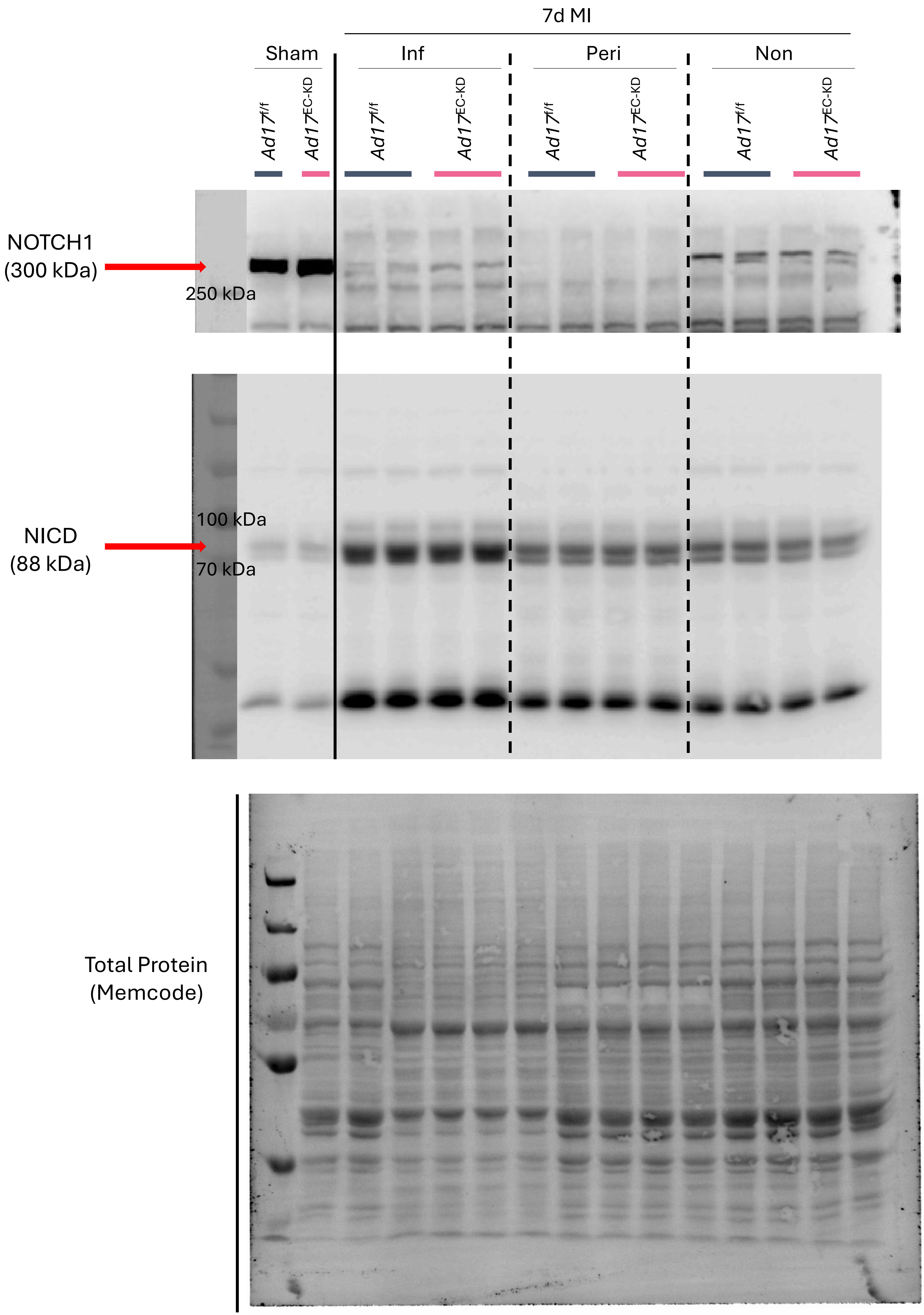

Fig_S8C: Fibronectin

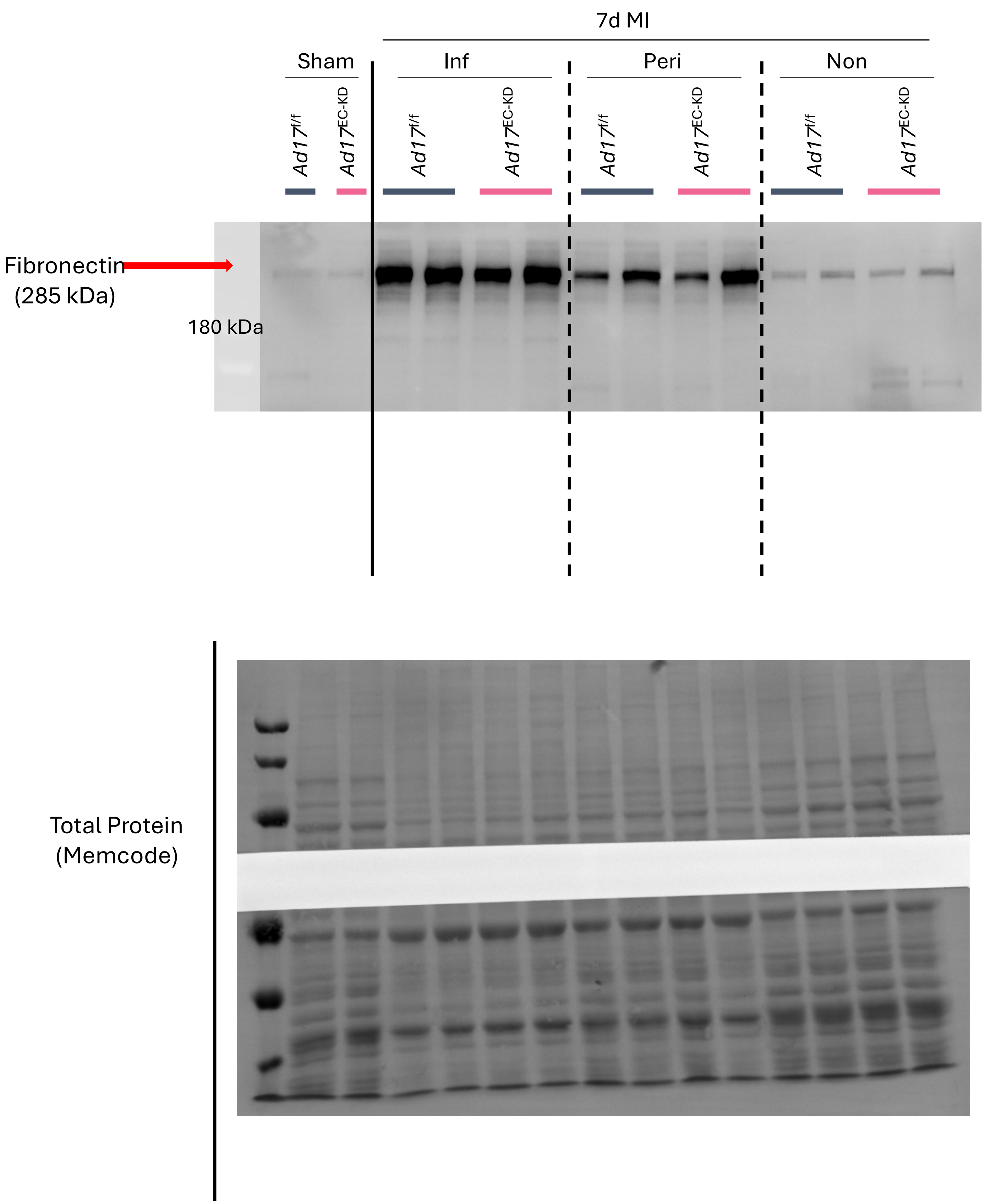

Fig_S8C: pSMAD2/3 SMAD2/3

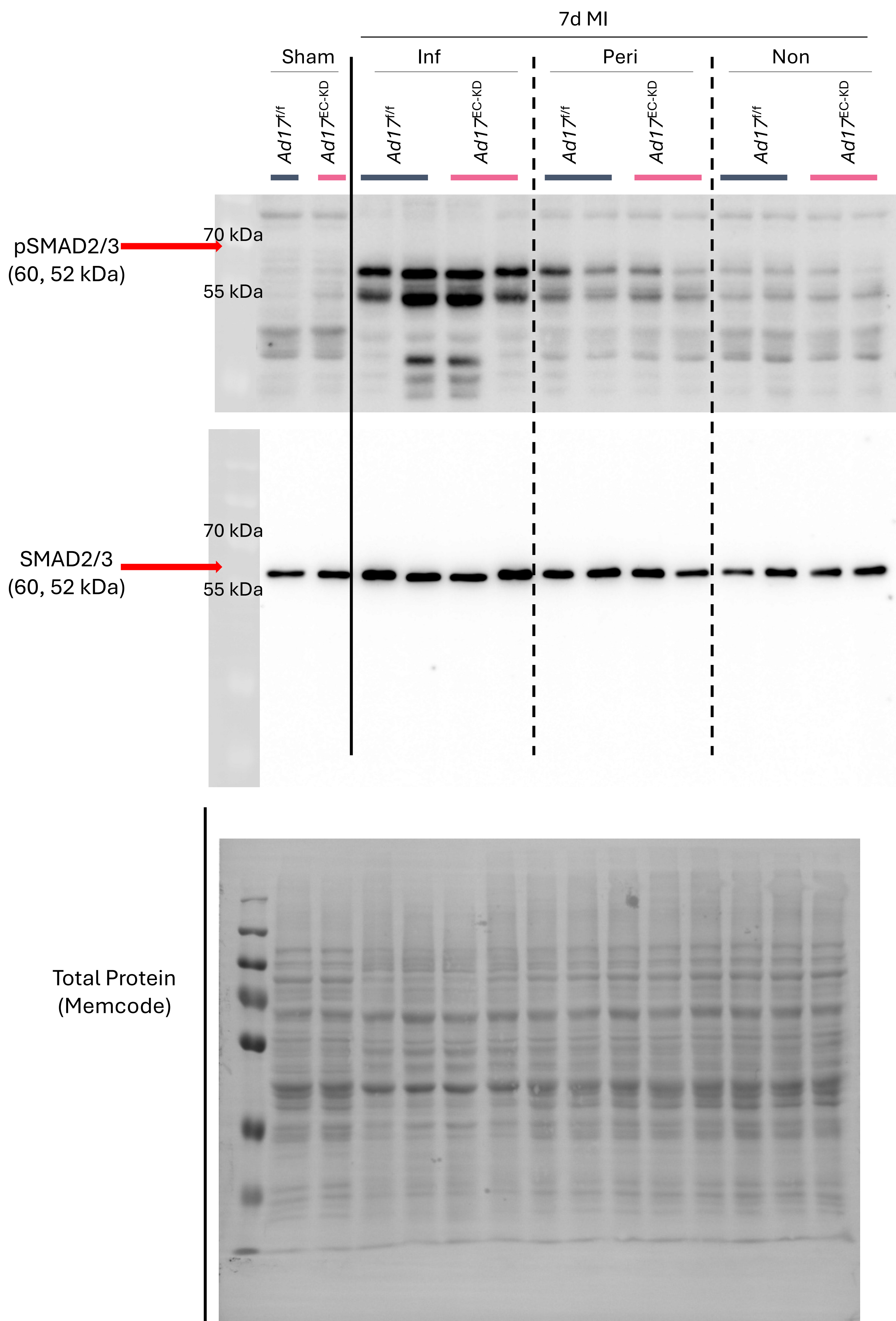

Fig_S11B (NEW): pMLKL MLKL

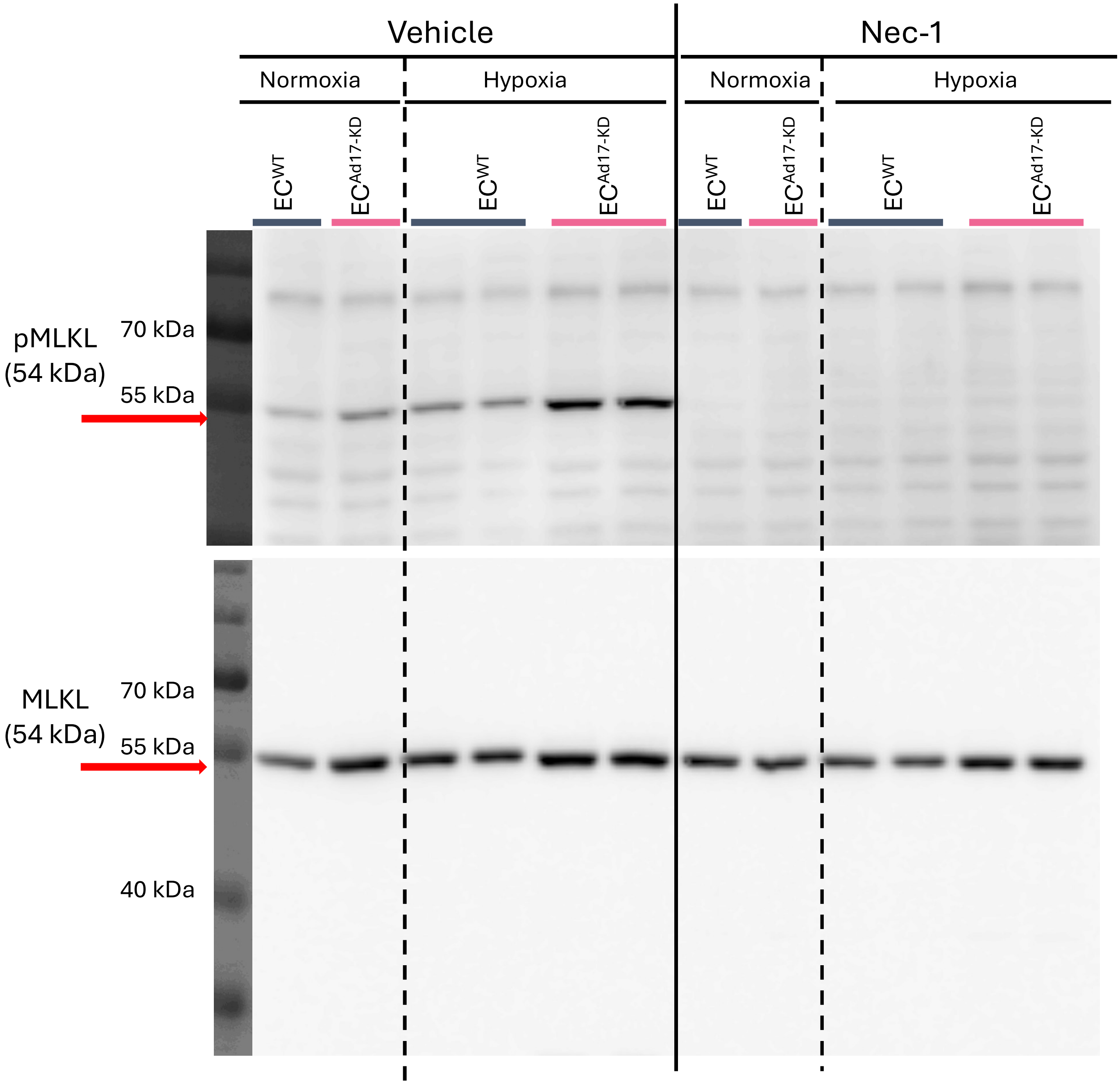

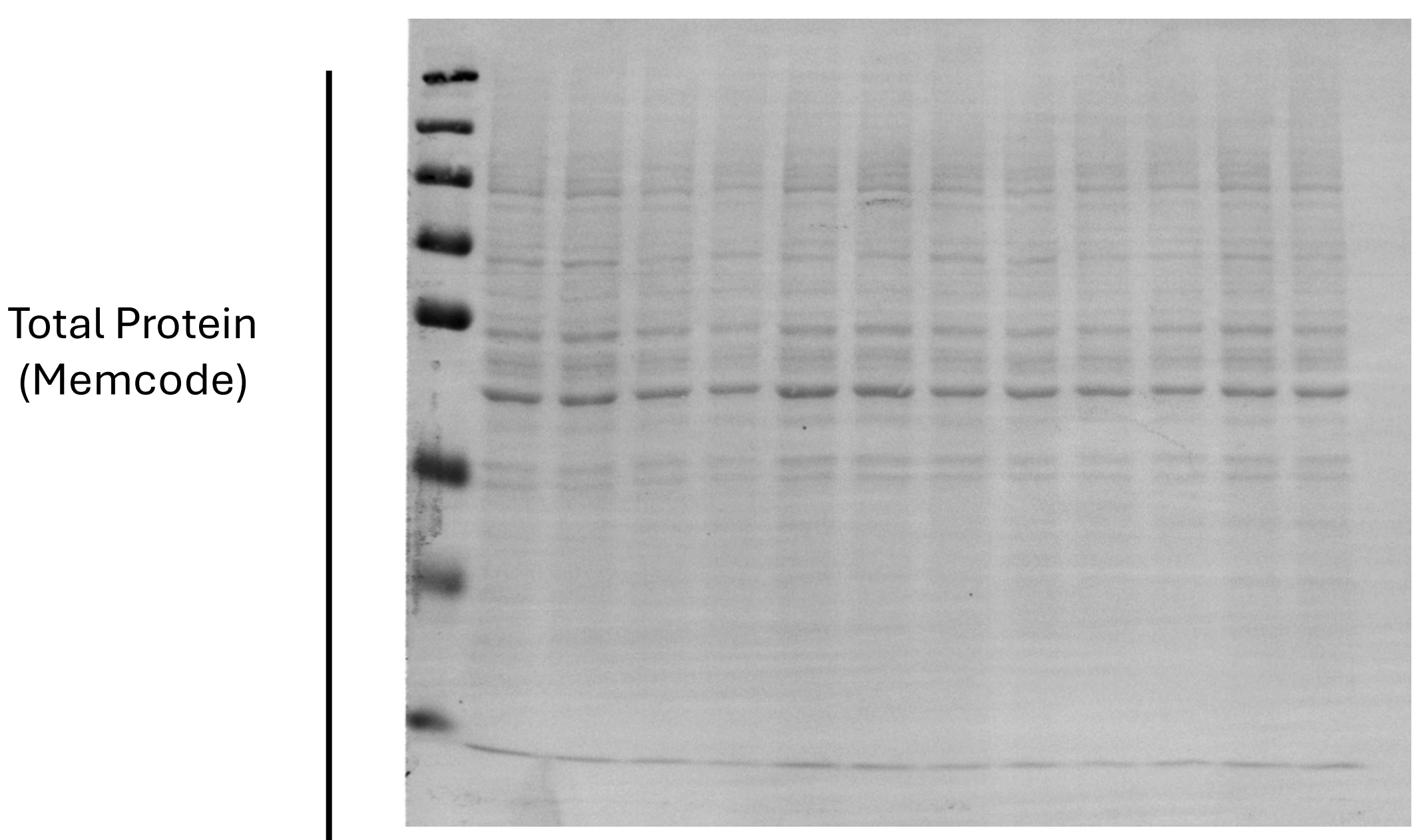

Fig_S12B (NEW): pSMAD2/3 SMAD2/3

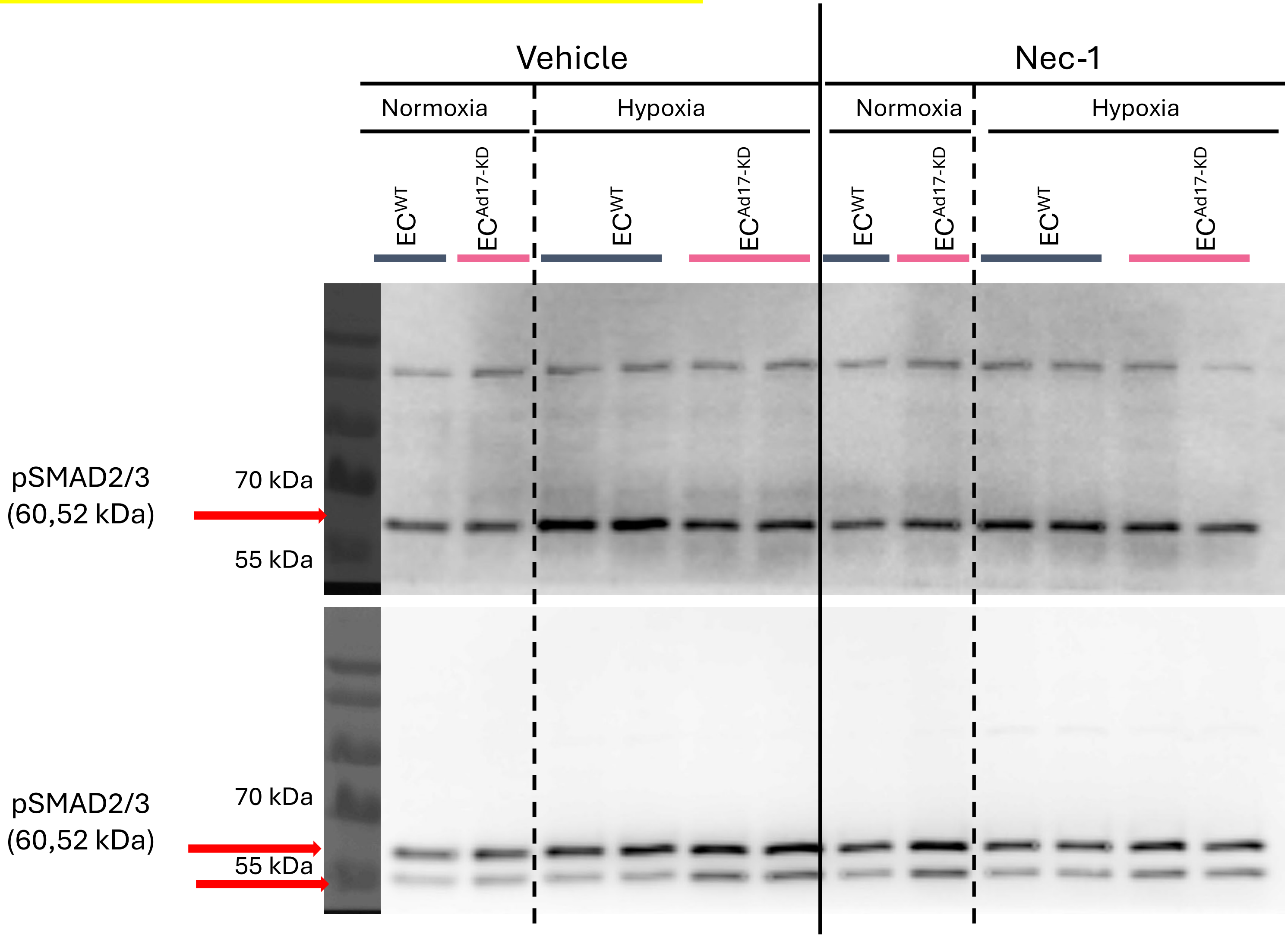

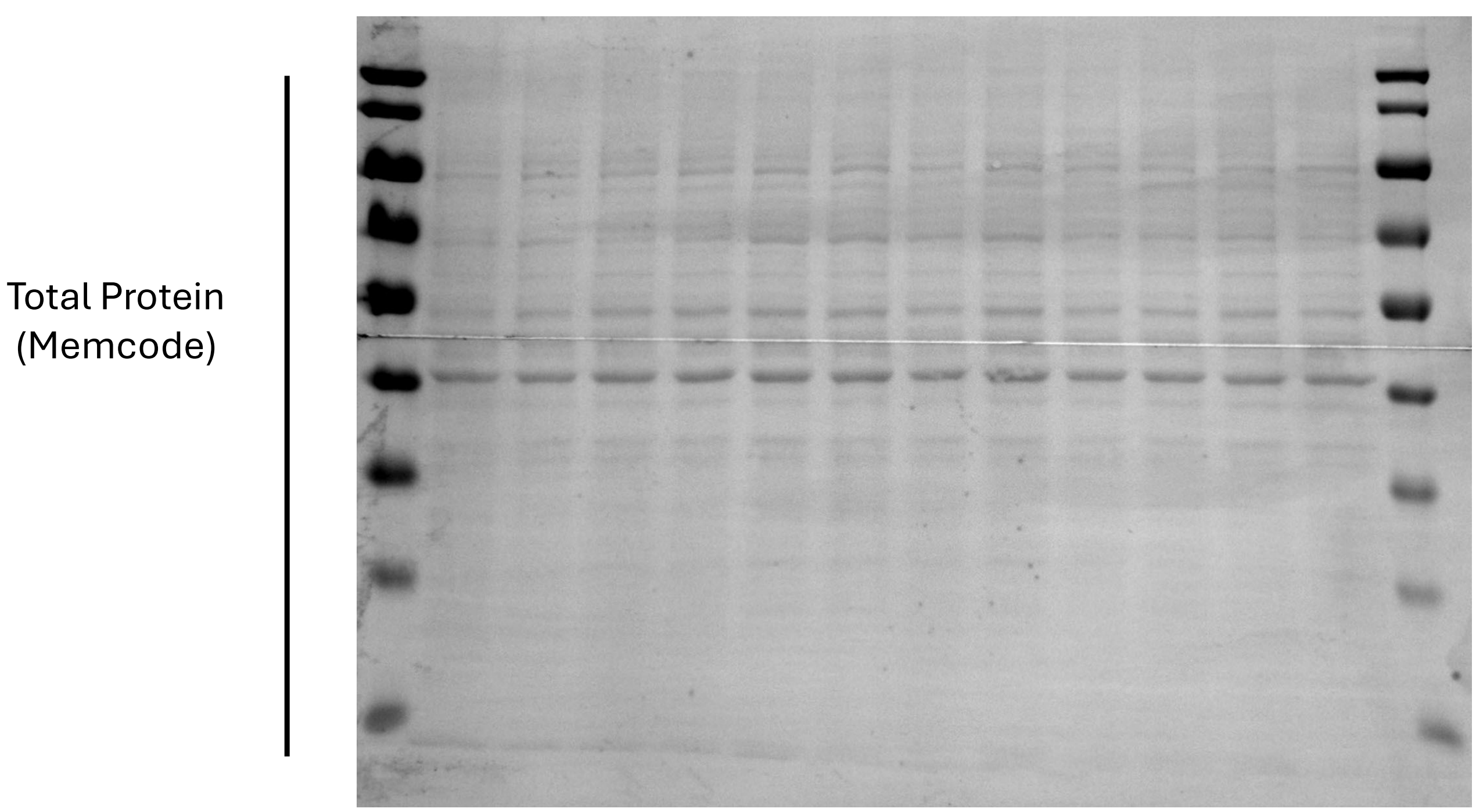

Fig_S12B (NEW): αSMA

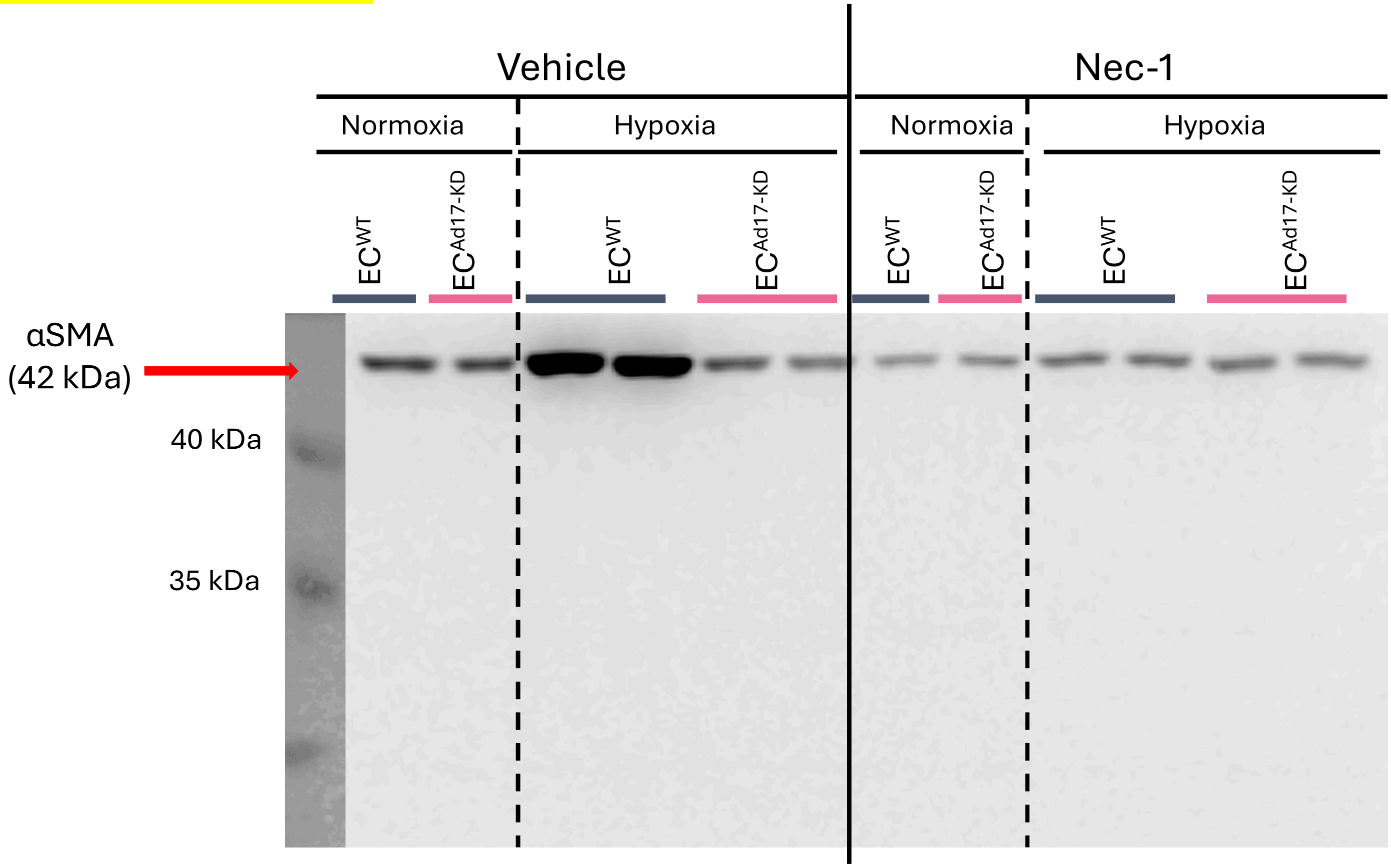

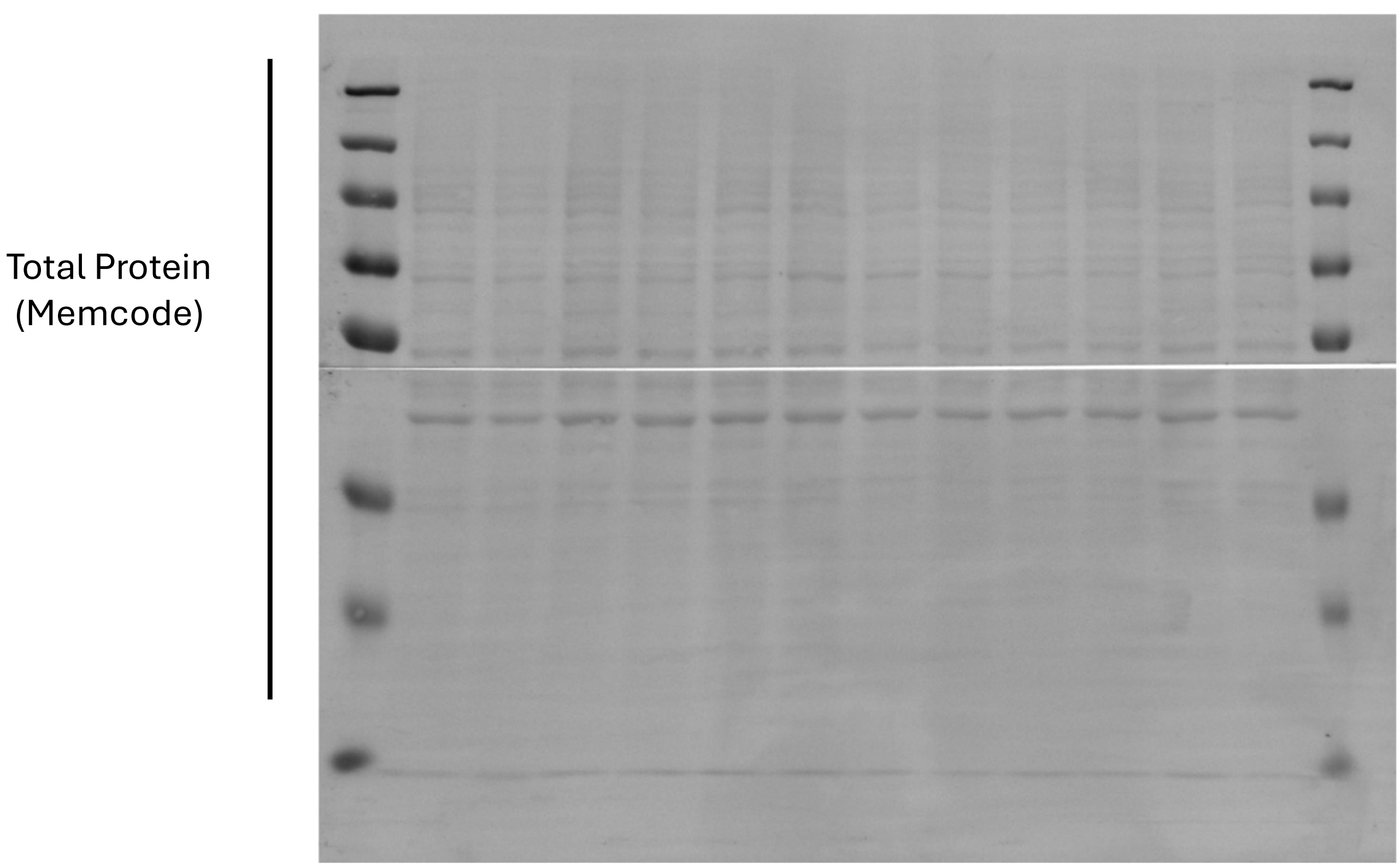

Fig_S12B (NEW): SM22α

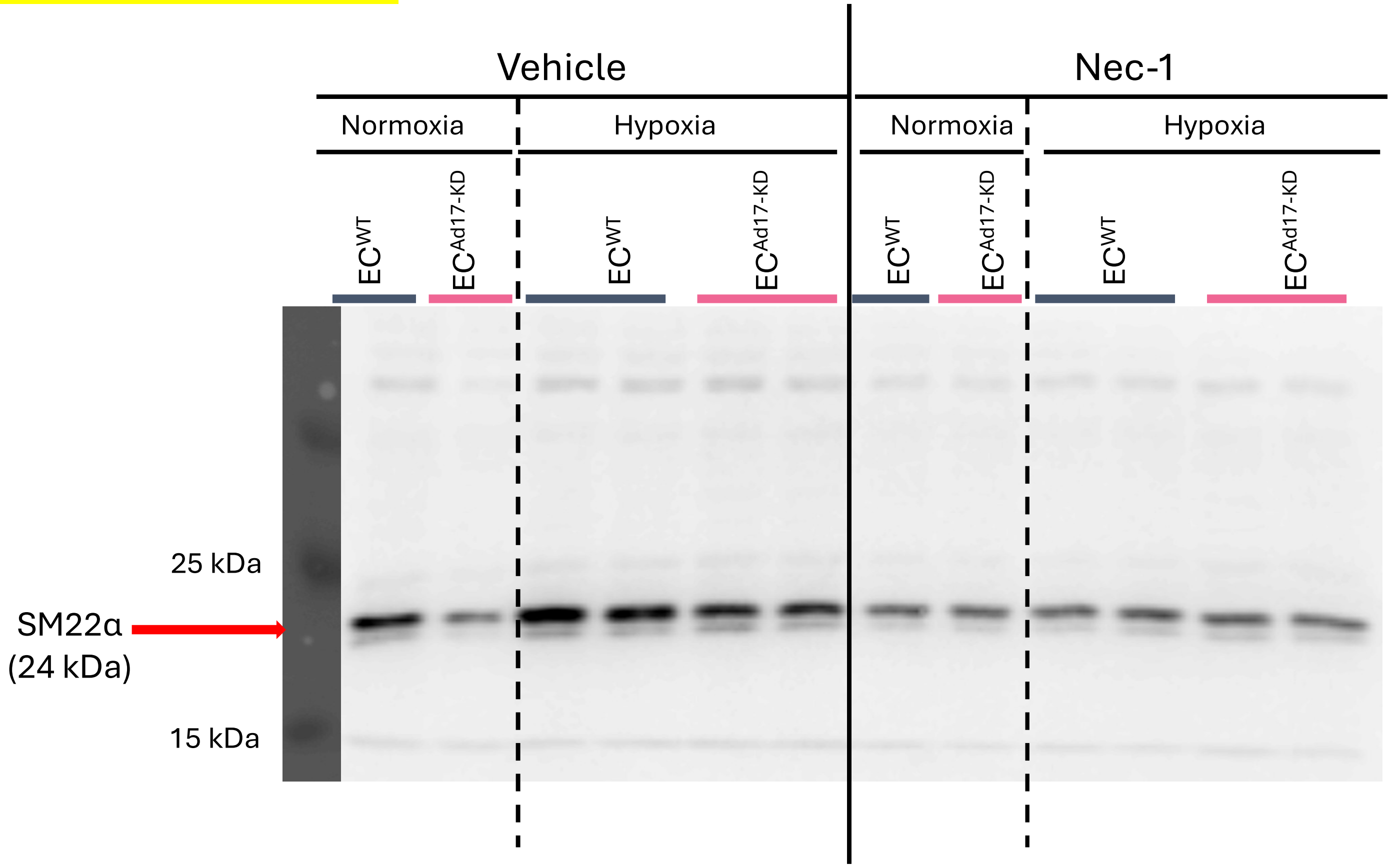

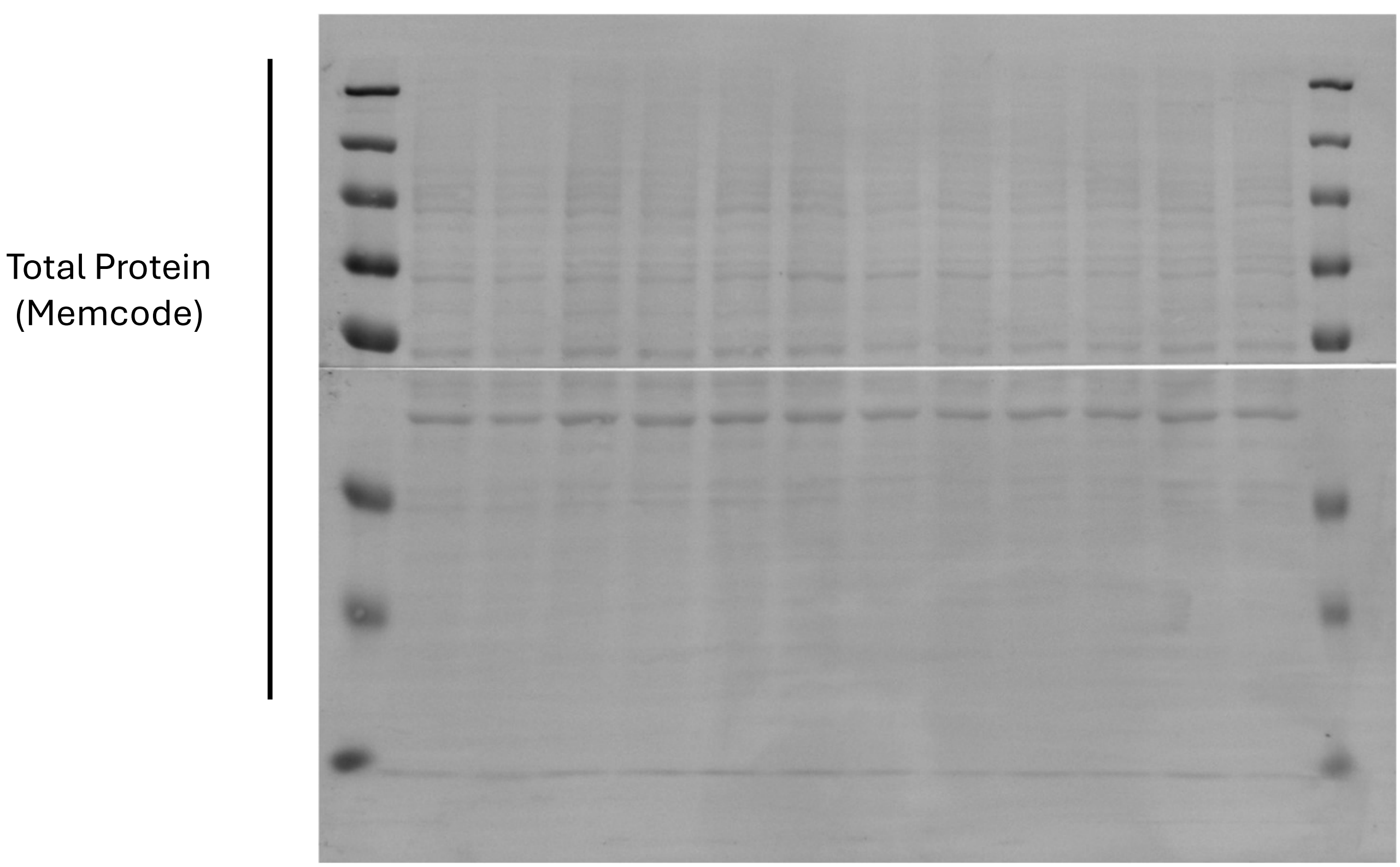

Supplement: Supplementary file 2 [file 41419_2026_8979_MOESM2_ESM.pdf]
